# Somatic Mutations Detected in Parkinson Disease Could Affect Genes With a Role in Synaptic and Neuronal Processes

**DOI:** 10.3389/fragi.2022.851039

**Published:** 2022-04-28

**Authors:** Irene Lobon, Manuel Solís-Moruno, David Juan, Ashraf Muhaisen, Federico Abascal, Paula Esteller-Cucala, Raquel García-Pérez, Maria Josep Martí, Eduardo Tolosa, Jesús Ávila, Raheleh Rahbari, Tomas Marques-Bonet, Ferran Casals, Eduardo Soriano

**Affiliations:** ^1^ Institute of Evolutionary Biology (UPF-CSIC), Barcelona, Spain; ^2^ Genomics Core Facility, Departament de Ciències Experimentals i de la Salut, Universitat Pompeu Fabra, Barcelona, Spain; ^3^ Department of Cell Biology, Physiology and Immunology and Institute of Neurosciences, Universitat de Barcelona (UB), Barcelona, Spain; ^4^ Centre for Networked Biomedical Research on Neurodegenerative Diseases (CIBERNED), Madrid, Spain; ^5^ Cancer, Ageing, and Somatic Mutation (CASM), Wellcome Sanger Institute, Cambridge, United Kingdom; ^6^ Department of Neurology, Hospital Clínic de Barcelona, Institut d’Investigacions Biomédiques August Pi i Sunyer (IDIBAPS), University of Barcelona (UB), Barcelona, Spain; ^7^ Centro de Biología Molecular Severo Ochoa, Madrid, Spain; ^8^ Catalan Institution of Research and Advanced Studies (ICREA), Barcelona, Spain; ^9^ CNAG-CRG, Centre for Genomic Regulation (CRG), Barcelona Institute of Science and Technology (BIST), Barcelona, Spain; ^10^ Institut Català de Paleontologia Miquel Crusafont, Universitat Autònoma de Barcelona, Barcelona, Spain; ^11^ Departament de Genètica, Microbiologia i Estadística, Facultat de Biologia, Universitat de Barcelona, Barcelona, Spain

**Keywords:** Parkinson disease, somatic mutations, brain mosaicism, neurodegenaration, somatic genome alteration

## Abstract

The role of somatic mutations in complex diseases, including neurodevelopmental and neurodegenerative disorders, is becoming increasingly clear. However, to date, no study has shown their relation to Parkinson disease’s phenotype. To explore the relevance of embryonic somatic mutations in sporadic Parkinson disease, we performed whole-exome sequencing in blood and four brain regions of ten patients. We identified 59 candidate somatic single nucleotide variants (sSNVs) through sensitive calling and a careful filtering strategy (COSMOS). We validated 27 of them with amplicon-based ultra-deep sequencing, with a 70% validation rate for the highest-confidence variants. The identified sSNVs are in genes with synaptic functions that are co-expressed with genes previously associated with Parkinson disease. Most of the sSNVs were only called in blood but were also found in the brain tissues with ultra-deep amplicon sequencing, demonstrating the strength of multi-tissue sampling designs.

## Introduction

Somatic mutations appear during development and tissue maintenance, making every individual a mosaic of cells with slightly different genomes. Early mutations occurring before gastrulation are shared by tissues of different germ layer origin (Lodato et al., 2015; Bae et al., 2018) and can cause disease (Kluge et al., 2019; Mensa-Vilaró et al., 2019). Mutations occurring later in development or during adult tissue maintenance that confer a proliferative advantage can produce clonal expansions of the cells carrying them, limited by each tissue’s growth dynamics (Lee-Six et al., 2019; Moore et al., 2020). Notably, this type of somatic mutations are the cause of cancer (Nowell, 1976) but have also been found in healthy skin (Martincorena et al., 2015; Abyzov et al., 2017), esophagus (Martincorena et al., 2018), colon (Lee-Six et al., 2019), liver (Brunner et al., 2019), endometrium (Moore et al., 2020) or lung (Yoshida et al., 2020). Further, organ-exclusive somatic mutations in the MTOR pathway are involved not only in cancer (Guertin and Sabatini, 2007) but also in neurodevelopmental disorders such as hemimegaloencephaly (Poduri et al., 2012) and focal cortical dysplasia (Lim et al., 2015). Other studies linking somatic mutations to disease did not determine whether the variants were of early or late origin, including a case of congenital arrhythmia in which the causal mutation was only tested in cardiomyocytes and lymphocytes and found in both cell types (Priest et al., 2016).

It has been recently described that adult neurons accumulate over 17 somatic single nucleotide variants (sSNVs) per year, in a process independent of cell division (Abascal et al., 2021). Even before birth, each human neuron carries a few hundred sSNVs (Bae et al., 2018) and those with a frequency higher than ∼2% can also be detected in tissues that originate from a different germ layer (Lodato et al., 2015), suggesting that brain development does not heavily rely on clonal expansions. Other types of somatic variation have also been found in neurons: retrotransposon mobilization is common and disproportionately impacts protein-coding loci (Baillie et al., 2011) and over three copy number variants (CNVs), mainly losses, can be found in each cell (Cai et al., 2014). While the number of CNVs decreases in brain with age (Chronister et al., 2019), sSNV load increases (Hoang et al., 2016; Lodato et al., 2018; Abascal et al., 2021), posing questions about their relevance in neuronal diversification, plasticity, and dysfunction.

The somatic variant hypothesis for neurodegenerative diseases states that unexplained sporadic cases could be caused by somatic mutations, presumably in the same genes affected in familial cases (Pamphlett, 2004). Supporting this theory, age-related sSNVs have been proposed to accumulate faster in neurodegeneration (Lodato et al., 2018), although more research is needed (Abascal et al., 2021). Besides these later-acquired mutations, earlier somatic mutations may also contribute to phenotypes and diseases. This is suggested by the fact that individuals with autism spectrum disorder have a higher burden of somatic mutations than their unaffected siblings, measured in blood (Dou et al., 2017). In Alzheimer, targeted sequencing of blood samples showed that somatic variants in autosomal dominant genes (such as *APP*) could explain ∼2% of cases (Nicolas et al., 2018). Other studies on blood and hippocampus exomes from Alzheimer patients showed numerous sSNVs (Parcerisas et al., 2014) and that over a fourth carried somatic mutations affecting pathways known to contribute to tau hyperphosphorylation (Park et al., 2019), demonstrating the power of exome analysis.

The link between Parkinson disease (PD) and somatic mutations is not as clear. Notably, only about 10% of cases can be attributed to monogenic forms (Lesage and Brice, 2009), and at most 30% of patients have an affected first-degree relative (Rocca et al., 2004). A study on 511 sporadic cases tested multiple brain regions with a sensitivity limit at 5% of variant allele frequency (VAF) and did not find any somatic variants in *SNCA*, the main causative gene in early-onset familial PD (Proukakis et al., 2014). On the other hand, patients showed high levels of heteroplasmic mitochondrial DNA deletions (Bender et al., 2006) and more *SNCA* somatic copy number gains in substantia nigra neurons compared to controls, which positively correlated with age of onset (Mokretar et al., 2018).

To explore the potential link between somatic SNVs and Parkinson disease, we sequenced the exome of five different tissues—substantia nigra, striatum, neocortex, cerebellum, and blood—from ten sporadic Parkinson patients to an average coverage of 60X. We developed and implemented a sSNV detection protocol based on both single tissue and joint information, COSMOS, which we used to identify 59 candidate sSNVs. Further amplicon-based deep sequencing confirmed 27 of them, with an average of 3 sSNVS per individual (range 0–6). Although our power was limited, the confirmed sSNVs were enriched in synaptic and axonal processes and patients with more sSNVs had a weak tendency to die earlier, suggesting a potential role of these variants in the disease. Interestingly, over 76% of the variants validated in multiple brain tissues were only called in blood in the exome data, demonstrating that the study of more accessible and unaffected tissues may well serve to identify variants with lower frequency in diseased organs.

## Results

### Dataset

**FIGURE 1:**
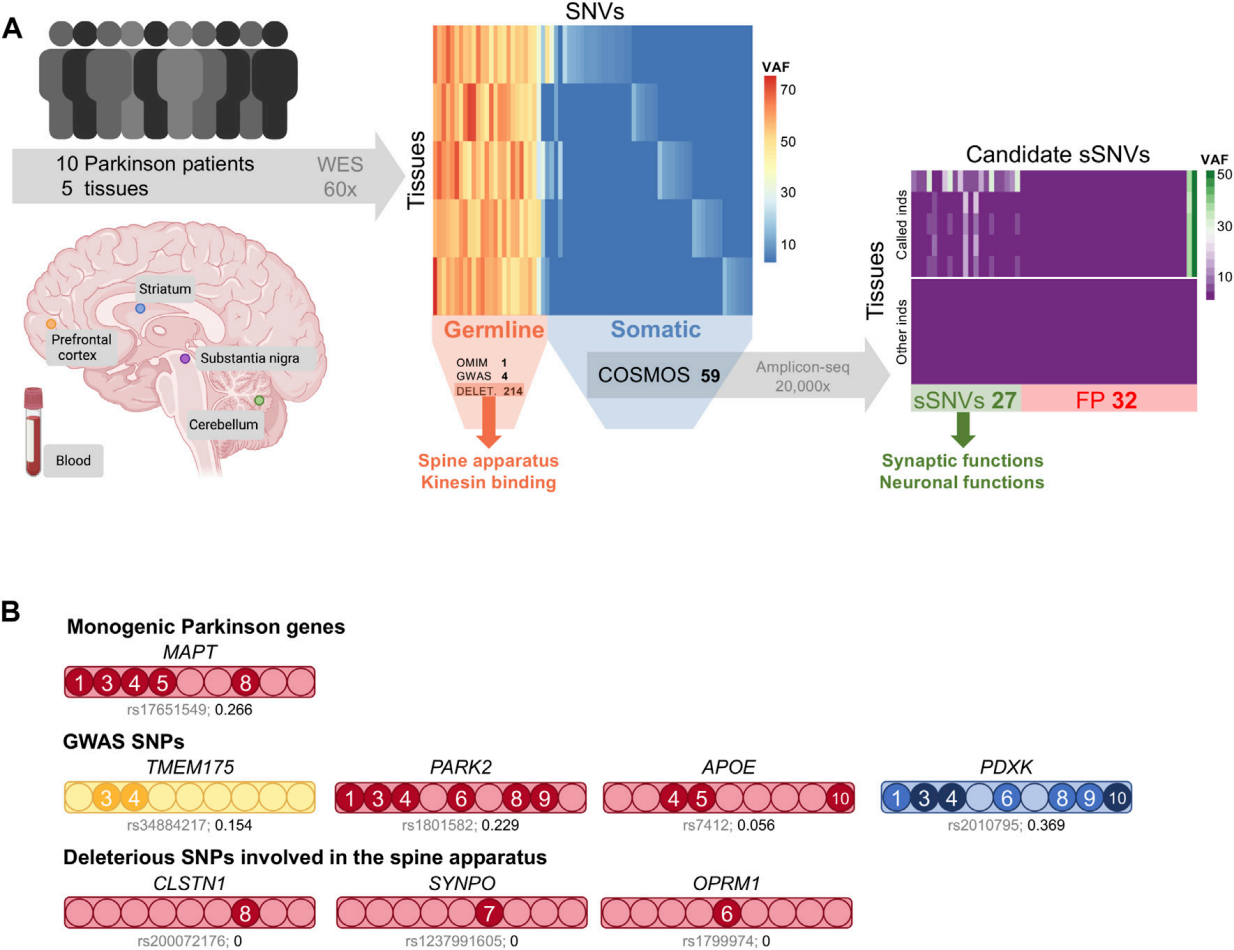
**(A)** Study overview. We sequenced the whole exome of blood and four brain regions from ten sporadic Parkinson disease patients. In this cohort, we found 1 germline SNP associated with Parkinson in OMIM and 4 *via* GWAS. The deleterious germline SNVs were enriched in spine apparatus and kinesin binding genes. Candidate somatic variants were carefully annotated and filtered using COSMOS, resulting in 59 candidate sSNVs. We used deep amplicon sequencing to validate them in all samples. The 27 validated sSNVs are enriched in synaptic and neuronal functions. VAF: variant allele frequency, FP: false positives. **(B)**. Germline SNVs related to Parkinson. A bar is shown for each germline variant, with its color indicating the type of variant: missense (red), splice acceptor (yellow), or intronic (blue). Filled circles indicate individuals carrying the variant (DV2 is not included) and darker colors indicate homozygous SNVs. The affected gene is indicated on top and the dbSNP identifier and the frequency of the alternative allele in the IBS population are shown below each bar.

We sequenced the whole exome of five different tissues from ten sporadic Parkinson patients to an average coverage of 60X. Blood was obtained from stored vials while the cerebellum, neocortex, substantia nigra, and striatum samples were collected during autopsies (Figure 1A). Patients’ median age was 81 at the time of death, they had varying ages at disease onset, and both sexes were represented (Supplementary Table S1). As expected, germline single nucleotide variant (SNV) calling resulted in clustering of tissue samples by individual except for two samples from individual DV2, which appear to be contaminated (Supplementary Figure S1). Accordingly, this individual was excluded from subsequent analyses but included in the sample set used to estimate background noise.

### Germline Variants are Associated With Parkinson

All patients were diagnosed with sporadic Parkinson because no affected first-degree relatives were known. This assessment did not include genetic analyses, so we evaluated their germline variants before considering their somatic mutational landscape. We used three different strategies to prioritize germline SNVs. First, we identified germline SNVs in genes linked to Parkinson in OMIM (www.omim.org) that were deleterious as indicated by a CADD score > 15 (Rentzsch et al., 2019). Only one variant met these criteria, rs17651549, a missense mutation in the *MAPT* gene, which encodes the tau protein, predicted as deleterious by multiple methods and at a highly conserved position in vertebrates (Supplementary Table S2). This variant, which was heterozygous in over half the patients, has been previously linked to PD by different means: multivariate family-based association tests (Wang et al., 2010), pathway analysis (Song and Lee, 2013), and targeted resequencing (Spataro et al., 2015). However, in a contradictory haplotype association analysis, it provided a reduced risk for PD (Li et al., 2018). This SNP is not rare in Europe; particularly, the frequency of the alternative allele in the 1000GP IBS population (Iberian populations in Spain) is 0.27, and similarly, it was 0.28 among the 10 Spanish Alzheimer patients included in this study (Figure 1B).

We made use of the greater statistical power of previous GWAS studies to identify Parkinson-associated germline variants (41 variants, Supplementary Table S3) (Tan et al., 2010; Do et al., 2011; International Parkinson Disease Genomics Consortium et al., 2011; International Parkinson Disease Genomics Consortium and Wellcome Trust Case Control Consortium 2, 2011; Lill et al., 2012; Nalls et al., 2014; Chen et al., 2016) and found four in our cohort (Figure 1B). We found a splice acceptor variant in *TMEM175*, rs34884217, in DV3 and DV4. This variant, predicted to affect nonsense-mediated decay, has been associated with Parkinson (Nalls et al., 2014; Heckman et al., 2017) and *TMEM175* deficiency is linked to the increase of α-synuclein aggregation (Jinn et al., 2017), suggesting a possible causal link. A missense mutation of *PARK2* showed the highest odds ratio for Parkinson disease in Europeans in a meta-analysis (Ramakrishnan et al., 2016). The other two SNVs have been associated with the disease (Huang et al., 2004; Elstner et al., 2009; Williams-Gray et al., 2009), but conflicting results have also been reported (Guella et al., 2010; Federoff et al., 2012).

In addition, all germline SNVs were filtered by deleteriousness (CADD > 15 and SIFT prediction) and their frequency in the 1000GP European population (<0.1). The resulting 214 variants affected 207 genes, with each patient carrying a median of 27 deleterious and rare variants (range 20–32). An overrepresentation enrichment analysis (Zhang et al., 2005) detected seven molecular function and cellular component terms significantly enriched in this gene set (FDR ≤ 0.05, Supplementary Figure S2). Remarkably, the term with the highest enrichment ratio was *spine apparatus* (Figure 1B), a derivate of the smooth endoplasmic reticulum generally present in dendritic spines that seems to participate in spine remodeling in Parkinson disease models (Smith et al., 2009). Kinesing binding and motor activity were also among the significantly enriched terms, with seven out of the nine individuals carrying a deleterious mutation in the genes driving these associations. These genes have been previously linked to PD in different studies. As an example, *CLSTN1* overlapped a significantly hypomethylated CpG (Chuang et al., 2017), was differentially expressed (Kong et al., 2018), and carried a missense mutation (YemniAl et al., 2019) in PD cases. Several dynein and kinesin proteins also appear to be relevant in PD (Pellegrini et al., 2016). All of this highlights the complexity of Parkinson disease, in which common and rare variants affect multiple pathways that seem to contribute to the phenotype. Abundant data is needed to uncover these associations and the evaluation of somatic mutations has the potential to contribute to this effort.

### Somatic Variant Calling

Somatic variant calling was first developed for cancer, and standard approaches are based on paired tumor and normal samples comparison (Krøigård et al., 2016; Xu, 2018). Growing evidence shows that low frequency embryonic somatic mutations are present in multiple tissues, even from different germ layers (Lodato et al., 2015; Bae et al., 2018), so paired comparisons are inadequate in scenarios with no or limited clonal expansions. Requiring a minimum of two supporting reads in our 60X data would limit our sensitivity to a mean VAF over 3%. Variants with that frequency could have originated before gastrulation, be present in multiple tissues and require joint sample calling. On the other hand, blood clonal expansion, especially prevalent in old age (Jaiswal et al., 2014), will result in variants private to this tissue, requiring single sample calling. In standard paired samples calling, shared calls are essentially discarded, which filters out germline variants and recurrent artifacts. The main obstacle unpaired single or joint sample calling have is precisely differentiating these confounding factors from true somatic variants.

Germline callers are optimized to discard variants that do not fit with set VAF expectations. Low frequency variants, such as those we are interested in, are considered sequencing noise or contamination, and discarded. For this reason, we called variants in all samples using HaplotypeCaller with the ploidy parameter set to 10, which increases its sensitivity to lower frequency variants, as previously shown (Wang et al., 2021). We also used VarScan 2 with lax parameters (-min-coverage 1-min-reads2 1 -p-value 1 -min-var-freq 0.000001). The resulting call set is mostly composed of germline heterozygous SNVs, calls within CNVs, recurrent sequencing errors, and other artifacts besides the somatic SNVs. Hence, we developed a filtering strategy to identify high confidence somatic SNVs, COSMOS (Combined Or Single sample MOSaicism detection, available in https://github.com/ilobon/COSMOS). COSMOS can be used to annotate the relevant information needed to identify reliable somatic candidates on standard VCF files and to filter calls. Detection of reliable variants can be performed with a single sample approach, a joint filtering approach (using multiple samples from the same individual), or both.

### COSMOS

Manual inspection of the calls obtained from HaplotypeCaller and VarScan 2 allowed the identification of multiple sources of artefacts (some of which have been addressed by other studies (Chen et al., 2017; Kim et al., 2019; Park et al., 2019; Solís-Moruno et al., 2021; Wang et al., 2021)), for which we devised filtering approaches. The rationale was that even if our patients had the same disease and could carry mutations in the same pathways or even genes, the probability that they would bear somatic mutations in the exact same position was negligible in just 10 individuals. Hence, variants present in multiple individuals were presumed to be artefacts.

We discarded all off-target calls because they have higher strand imbalances (69.4% of off-target calls fail the Poisson test *vs*. 39.5% of on-target calls) and read pair imbalances (21.8 *vs*. 4.4% failing the read pair ratio filter). The most frequent confounding factors for on-target calls were germline heterozygous SNVs, CNV regions, unresolved regions of the genome and regions that are more difficult to align (indels, homopolymers).

**FIGURE 2:**
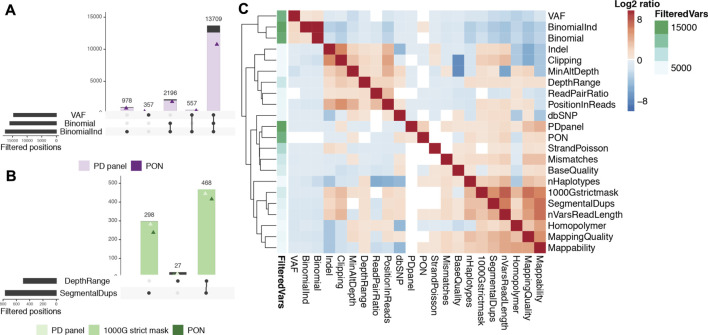
Relevance of COSMOS filters in our Parkinson exome data. **(A)**. Germline SNVs. Intersection of the main criteria identifying germline heterozygous point mutations: VAF (high variant allele frequency), Binomial (non-significant binomial test for allele depths) and BinomialInd (non-significant binomial test in at least one sample from the individual). For each intersection, the number of variants also found in the other individuals of our dataset (PD panel, filled in lavender) and an external panel (PON, purple triangle) are shown. **(B)**. CNV regions. Intersection of DepthRange (most extreme depth variants) and SegmentalDups (segmental duplications WGAC track) with PD panel and PON (light and dark green triangles, respectively) and variants present in the 1000GP strict mask filled in green. **(C)**. Relationship between criteria. Log2 ratio between the number of variants failing both the row and the column criteria and those failing the row criterium only. Higher log2 ratios (red) denote higher co-occurrence of criteria failures. The annotation column (green gradient) indicates the total number of positions failing each of the row criteria.

Germline heterozygous calls comprised at least 93.1% of our on-target calls. They can be easily distinguished when their VAF is close to or higher than 50%. However, germline calls’ VAFs are more over dispersed when the coverage is low (Supplementary Figure S3) and VAFs as low as 18% can result from germline variants sequenced at 60X (and even as low as VAF = 10% for 20X, our lower bound depth). We used a binomial test (discarding 83.2% of on-target calls) and a VAF upper limit (76.5% of on-target calls had VAF > 40%) to identify the most obvious germline variants, with a large overlap between the two filters (Figure 2A). These variants are common to all tissues from the same individual, so when multiple samples are available, requiring that all pass the binomial test greatly increases the detection power, following an exponential distribution (Supplementary Figure S4). We discarded an additional 5.5% of variants using this approach (first bar in Figure 2A). In addition, most of these calls can also be flagged by the number of called individuals. We generated a population and batch specific panel for each patient using the 45 samples from the other 9 PD individuals, and found it identified 92.1% of the germline variants previously discarded. Alternatively, an external panel of normals (PON) consisting of 428 whole genomes sequenced at the Sanger Institute identified 82.6% of them (Figure 2A).

Regions with CNVs can create artefactual somatic variants when reads are collapsed, mapped into the single copy in the reference genome. CNV callers can be used to identify the highest confidence copy number alterations, but more sensitive approaches are needed to detect noisy regions and obtain high-confidence somatic variant calls. We excluded regions with extremely low or high coverage (keeping 75% of the distribution) or overlapping the WGAC segmental duplications track, which filtered out 60.4% of the remaining on-target non-germline calls. We found 97.5% of these calls overlapped the 1000GP strict mask, which identifies regions of the genome with recurrently higher/lower coverage or lower mapping quality, showing its suitability as an alternative. Most calls failing our CNV filters were called in multiple individuals (94.1% in our PD panel and 84% in the independent PON) (Figure 2B). Other features useful to identify these artifacts are a high number of variants within read length distance, an imbalance in the number of mismatches in reads carrying each allele or in the proportion of clipped reads, and a low mappability (Supplementary Figure S5).

Unresolved regions of the genome or highly variable duplications can result in collapsed mapping with no significant increment of coverage because only reads spanning the homologous region and carrying few variants will be mapped. These are easily identified by a biased position of the alternative allele in the reads, clustered at read ends. We defined a position-in-reads bias score (PIR) to address this issue (see Methods). Other features targeting these artifacts are allele clipping imbalances, a high number of variants in the vicinity, more than three haplotypes found, allele imbalances for mapping quality, and read pair imbalances (Figure 2C). Local alignment around indels and homopolymers is challenging, and technical and biological noise are difficult to distinguish, so we discarded close-by variants to increase our call set confidence (see Methods). Finally, variants with too few reads supporting the alternative allele cannot be distinguished from random noise, and their imbalances cannot be evaluated, so a hard cut-off was applied (details in Methods).

The recommended best practices for calling somatic mutations in unpaired samples or when variants are expected in all samples consists of a non-stringent variant calling followed by several filters (Wang et al., 2021). Many of the features we identified have been previously targeted by manual filters in similar contexts (Chen et al., 2017; Kim et al., 2019; Park et al., 2019; Solís-Moruno et al., 2021; Wang et al., 2021). To facilitate their implementation, we developed COSMOS, a pipeline that can be used to consistently annotate and filter the described read features on VCF files (https://github.com/ilobon/COSMOS). Its main advantages are that it automates the process and that it provides the option to filter variants that pass all required filters in (1) single samples; (2) a minimum number of samples, which can be different sample sets for each filter; or (3) in either single of multiple samples.

### Most Somatic Mutations Appear to Have a Pre Gastrulation Origin

**FIGURE 3:**
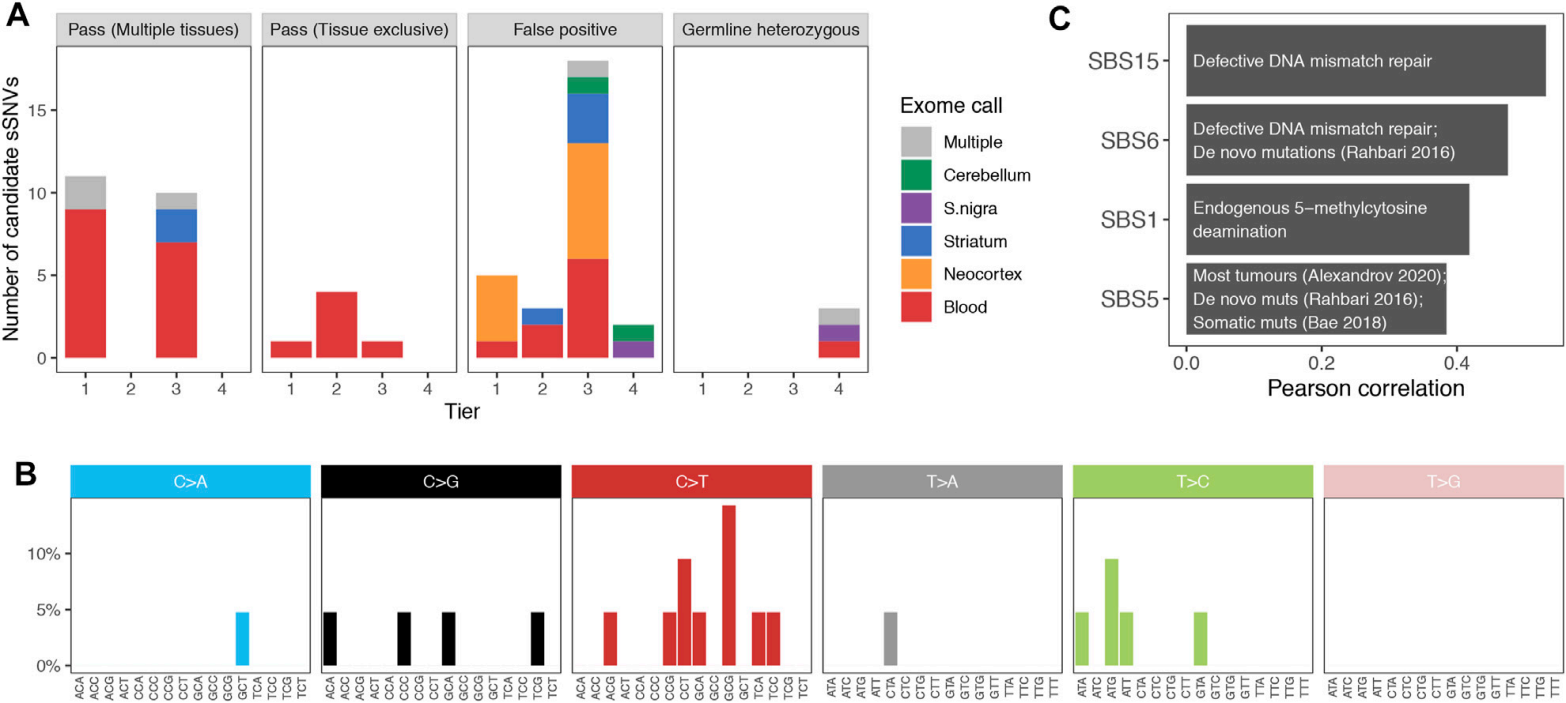
**(A)**. Validation of candidate variants. Number of mutations validated (Pass) in multiple or a single tissue or found to be false positives or germline heterozygous variants with amplicon sequencing data. Variants are distributed by calling-confidence tier, and colors indicate the tissue in which the variant was originally called in the exome data. **(B)**. Mutational spectrum of somatic SNVs in Parkinson brains. Variants present in the brain of Parkinson patients, segregated by substitution and trinucleotide context. **(C)**. Correlation with COSMIC signatures. Moderate Pearson correlations (r > 0.3) between the spectrum of brain somatic variants and the single base signatures (SBS) from COSMIC. Text describes the etiology or studies relevant for each signature.

A total of 59 variants passed COSMOS filtering across samples from the ten Parkinson patients. 7 of which were called exclusively by VarScan 2. After manual inspection, we classified variants in four tiers of confidence based on the presence of mismatches in the region and the features of the mutant supporting reads (including other variants in phase, strand, orientation, region covered; examples in Supplementary Figures S6, S7). Tier 1 had 17 high confidence variants, tier 2 consisted of the 7 VarScan calls, tier 3 contained 29 lower confidence candidate sSNVs, and tier 4 included 5 putative false positives (Supplementary Table S4 and Methods). A median of 7 candidates were called in each individual (range 1–11), and perhaps unsurprisingly, given the limited coverage, 91.5% of all the candidate variants were called in just one tissue, mostly blood (61.1% of single tissue calls) (Figure 3A).

We evaluated 58 out of the 59 variants with the rhAmpSeq targeted amplicon sequencing system, which allows the multiplexed amplification of multiple genomic positions. This way, we sequenced data from all positions and all patients at a mean coverage of over 18,000X so that non-called individuals could be used as negative controls. Four of the samples could not be amplified enough and had low coverages (mean ≤ 2,300X, Supplementary Figure S8). Variants were considered as validated when the support for the alternative allele was higher than in other patient’s samples and if their VAF suggested they were not germline variants (see Methods).

A total of 27 somatic SNVs were validated using these criteria. Validation rates were 70.6% for tier 1 (12 out of 17), 57.1% for tier 2 (4 out of 7), 37.9% for tier 3 (11 out of 29) and 0% for tier 4 (0 out of the 5 negative control variants). Per individual, a median of 4 variants were validated, and the median validation rate was 50% (mean of 40.6%, ranging from 0 to 83%). All calls from 3 individuals (DV4, DV5, and DV9) were false positives. DV9 had only one candidate sSNV call, but DV4 and DV5 had 7 and 6, respectively, demonstrating moderate interindividual variability. Only 6 of the 27 variants were present in a single tissue, which was always blood (Figure 3A, second panel). Interestingly, 76.2% (16 out of 21) of variants validated in multiple tissues had been called exclusively in blood in the exome data (Figure 3A, first panel), explained by their higher frequency in this tissue (mean difference of 10.8%, mean VAF in blood 11.5 vs. 0.7% in the other tissues). This was not a consequence of our calling method, as 68.8% of these variants (11 out of 16) had no read supporting the alternative allele in any brain sample but were then validated in at least 2 brain regions. This probably results from their random amplification in tissue maintenance of blood but could also be a consequence of depletion in the central nervous system.

### The Spectrum of Somatic SNVs in Parkinson

The discovery that different mutagenic agents–such as UV light, carcinogens or intrinsic cell processes–produce distinct substitution patterns in a context-dependent manner led to the development of mutational signature analysis (Alexandrov et al., 2013). To obtain the mutational spectrum of Parkinson disease, we combined the 21 sSNVs validated in at least one brain sample and classified them by substitution and trinucleotide context (Figure 3B). The number of variants was insufficient for mutational signature deconvolution, so we calculated its Pearson correlation with the COSMIC single base signatures. Since most of the identified variants are present in multiple tissues and are therefore of early origin, we expected to find a high similarity to signatures SBS1 and SBS5, as recently found in the mutational spectrum of early embryonic SNVs (Coorens et al., 2021). Indeed, we found both among the four moderately correlated signatures (Pearson’s r > 0.3, Figure 3C). SBS1 is a ubiquitous signature that results from the spontaneous deamination of methylated cytosines. SBS5 can be detected not only in most cancer samples (Alexandrov et al., 2020) but also in *de novo* mutations (Rahbari et al., 2016), somatic mutations (Bae et al., 2018), and in population level variants (Mathieson and Reich, 2017). In spite of our limited power in this analysis, signature SBS6 was also found to be highly correlated with the *de novo* mutational spectrum (Rahbari et al., 2016).

### Variant Allele Frequency of Somatic SNVs Reconstructs Tissue Relationships

**FIGURE 4:**
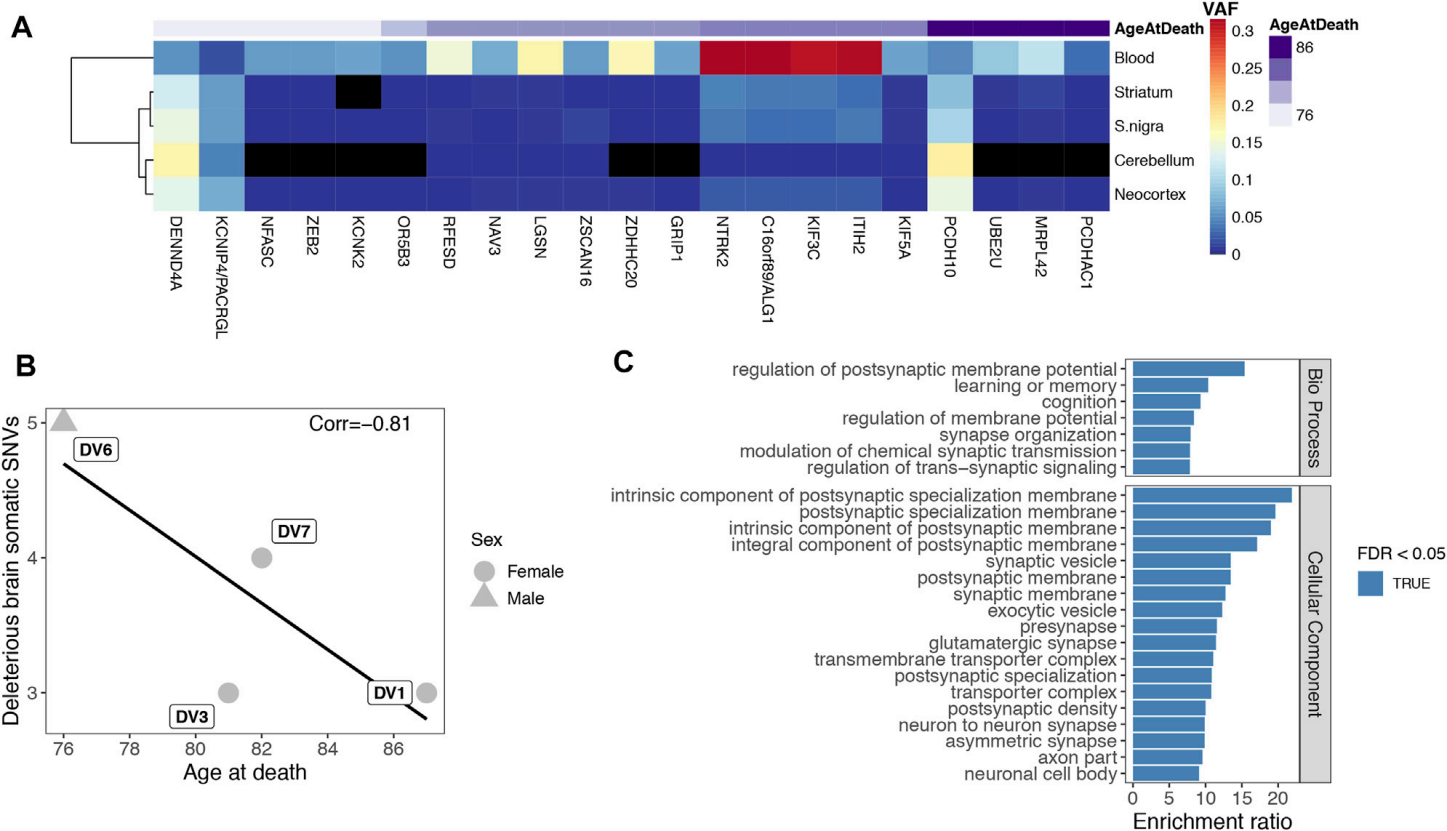
**(A)**. Clustering of tissues by VAF. Frequency of the 21 somatic variants found in brain was used to cluster the tissues. Genes are shown and variants are ordered by individual, with age at death shown on top. Black tiles indicate the variant did not pass all validation criteria in amplicon sequencing but could still have support in the tissue. **(B)**. Age correlation. Correlation between each patient’s number of potentially deleterious variants (nonsynonymous, in splicing consensus sites or in UTRs) found in brain and age at death. The line shows a non-significant fitted linear model (p-value = 0.19). **(C)**. Functional enrichments of extended gene set. Enrichment ratio of the top 25 terms by FDR of an overrepresentation enrichment analysis of the co-expression network extended gene set (*n* = 177 genes). The considered databases were Gene Ontology Biological Process, Cellular Component, and Molecular Function, Human Phenotype Ontology and disease GLAD4U.

We validated sSNVs in 6 out of the 9 subject individuals, ranging from 1 to 6 variants per patient, a modest yet sensible number for an exome analysis. All of DV10’s sSNVs were only detected in blood, but the other 21 variants (from DV1, DV3, DV6, DV7 and DV8) were detected in at least two brain tissues. We clustered the tissues based on their VAFs at these sSNVs. Because each individual has just a few variants, we pooled them together to analyze general tissue dynamics (Figure 4A). As expected from its clonal expansion, blood is the most distant tissue, with brain tissues being more closely clustered. Remarkably, striatum and substantia nigra, both affected in Parkinson disease (Bernheimer et al., 1973), cluster together, which could be caused by their closer developmental origin and/or more similar physiology. The presence of 3 variants with higher frequencies in all brain tissues compared to blood indicates that our findings are not the result of blood contamination in the other tissues. Importantly, blood was not called in the exome sequencing data for these variants, and the amplicon sequencing confirmed the VAF distribution in the different tissues.

### The Possible Impact of Deleterious sSNVs in Brain

We then tested the relationship between the number of potentially deleterious brain variants and individuals’ age at death. We excluded synonymous sSNVs (4 variants, including the only sSNV validated in the brain samples from patient DV8, Supplementary Table S4) and intronic variants not affecting donor or acceptor sites (2 variants), leaving 11 nonsynonymous sSNVs, 3 variants affecting splicing consensus sites, and 1 variant in a UTR region, as these are more probable to affect age at death. The age of onset was unknown for one of the four individuals included in this analysis, DV1. As age of onset is highly correlated with age at death (Pearson’s r = 0.9), we used it to evaluate the correlation between age and the number of sSNVs. Although counts of deleterious somatic variants identified in brain were negatively correlated with age at death (Figure 4B, Pearson’s r = −0.81), the trend was not significant (*p*-value = 0.19), unsurprisingly given the small data set.

### Genes Carrying sSNVs in Parkinson are Involved in Synaptic Processes Functions

Out of the 21 sSNVs found in brain, 4 nonsynonymous variants were predicted to be deleterious by SIFT. Remarkably, three of the genes carrying these variants are related to processes relevant in the brain. *GRIP1* is involved in transmission across chemical synapses and the regulation of neuron projection arborization (Geiger et al., 2014). *KCNK2* (or TREK-1) encodes a voltage-independent potassium channel essential in securing saltatory conduction at high frequency on myelinated afferent nerves (Kanda et al., 2019). *UBE2U*, a ubiquitination enzyme, is a candidate regulator of chromatin responses at double strand breaks (Guo et al., 2017), which are of fundamental relevance for gene expression in the brain (Madabhushi et al., 2015). Furthermore, ubiquitination dysfunction has been linked to Parkinson (Geisler et al., 2014) and Alzheimer (Gómez-Ramos et al., 2015). The other variant is in *DENND4A*, a secondary guanine nucleotide exchange factor that activates Rab-10, participating in the insulin-regulated glucose transporter GLUT4 translocation to cell membranes (Sano et al., 2011).

We explored the biological pathways potentially affected by the identified somatic mutations with an overrepresentation enrichment analysis. For this analysis, we only included the 15 sSNVs that result in nonsynonymous changes, could affect splicing consensus sites or in UTR regions and were validated in a brain tissue. Top enriched terms included “glial cell projection”, “axonogenesis” and similar processes (Supplementary Figure S9). However, as expected from such a small set of genes, none of the enrichments were significant (FDR ≤ 0.05). To gain power and retrieve the GO terms that better describe the functional context of the validated sSNVs, we expanded the gene set by adding genes with significantly high co-expression scores (above 900) as reported in the STRING database (Szklarczyk et al., 2019), resulting in a total of 177 genes. Performing an enrichment analysis with the expanded gene set, we found significant results including multiple synaptic terms (top 25 in Figure 4C), showing that our validated sSNVs affect genes tightly connected to the protein networks associated with these functions. Besides the Gene Ontology databases, to test a more direct relationship of our expanded gene list with phenotypes and diseases, we also included the Human Phenotype Ontology and the GALD4U disease databases. Chorea, dyskinesia and tremor were among the significantly enriched phenotypes and neurodegenerative diseases, and Parkinson disease among the GLAD4U database enriched terms (Supplementary Table S5).

To further investigate the tissue specificity of the genes harboring the 15 validated sSNVs, we performed a tissue enrichment using TissueEnrich (Jain and Tuteja, 2019). In accordance with the functional enrichment, cerebral cortex was the only significantly enriched tissue (adjusted *p*-value = 0.003 and Supplementary Figure S10).

To explore the consequences of the validated variants, we examined their effect on protein structures and found two interesting cases. All tissues from patient DV7 carried a mutation (p.Gly998Glu) in *KIF5A* (Uniprot Q12840), a kinesin heavy chain protein. Using Genome3D (Lewis et al., 2015), we found a DomSerf (Buchan et al., 2013) structural prediction for the region containing this amino acid (913-1032, confidence 100). The affected residue is in the surface of this small globular C-terminal domain (Supplementary Figure S11A). This region interacts with kinesin adaptor proteins such as TRAK1 and TRAK2, which mediate cargo binding (Hirokawa and Noda, 2008), suggesting the possible relevance of this sSNV. In addition, all DV1’s tissues but cerebellum carried a substitution in UBE2U (Q5VVX9), p. Pro96Ala, which is included in a DomSerf structure of residues 2-157 (confidence 100) and appears to be close to the active site. Although it seems improbable that the structure of the active site changes as a consequence of this mutation (Supplementary Figure S11B), its flexibility or orientation may be affected.

## Discussion

In this study, we explored the presence of somatic mutations in a cohort of ten Parkinson disease patients and identified and validated 27 somatic SNVs whose functions are enriched in synaptic processes, suggesting a potential role of somatic variants in Parkinson disease. Massive parallel sequencing makes it possible to characterize somatic genetic variation and study its role genetic disorders. These techniques have overcome the main limitation of first-generation sequencing technologies and are potentially capable of detecting somatic variants at very low frequencies if sufficient sequencing depth is generated. Thanks to this, the role of somatic mutations in a variety of diseases is becoming increasingly clear (Gleeson et al., 2000; Messiaen et al., 2011; Poduri et al., 2012; Priest et al., 2016; Bar et al., 2017; Nicolas et al., 2018; Mensa-Vilaró et al., 2019), including neurodegenerative (Park et al., 2019) and neurodevelopmental disorders (Dou et al., 2017).

However, somatic SNV calling from sequencing data outside the framework of paired samples still poses a challenge. Single cell and single-cell-derived colony sequencing suffer from amplification and *in vitro* growth artifacts, respectively (Abascal et al., 2021), and the single-molecule approaches necessary to avoid them are still expensive (Abascal et al., 2021). Somatic detection calling in unpaired bulk sequencing samples requires identifying bona fide mutations in a haystack of artefacts. Multiple studies have addressed false positives and used filters similar to those described here (Park et al., 2019; Wang et al., 2021; Solís-Moruno et al., 2021; Kim et al., 2019), but it can still be challenging to implement them in a consistent manner and apply them in a joint calling scenario. Here we present COSMOS (https://github.com/ilobon/COSMOS), a computational approach to accurately identify somatic mutations from bulk sequencing data that is highly customizable to each experiment’s requirements. We highlight the relevance of including a panel of individuals to help identify germline variants and recurrent artifacts, which are far more common than somatic mutations at the sensitivity a reasonable sequencing coverage provides. We also demonstrated that leveraging multiple samples from the same individual can help increase the accuracy and sensitivity to identify sSNVs, as previously suggested (Kim et al., 2019). Besides technical considerations, somatic mutations causing neurodegeneration could be depleted in the affected organs, so using samples from unaffected tissues might be key for their discovery. Indeed, some of the somatic variants we validated did not have enough support in the brain tissues’ exome data, and it was only the information from blood that allowed their detection. This could result from their random amplification in tissue maintenance of blood, especially in older aged individuals who show higher levels of clonal hematopoiesis (Watson et al., 2020). Including a wider variety of unaffected tissues in future studies will be important to clarify this scenario.

A possible confounding factor in this type of analysis is blood contamination, which would result in the presence of the same somatic mutations at lower frequencies in other tissues. Our results show that three of the validated variants have consistently higher VAFs in the brain tissues than in blood, demonstrating their presence in brain cells is more frequent than in blood. Besides their synaptic functions, the mutational spectrum, although underpowered, was similar to that of embryonic somatic mutations. Again, it would be useful to explore other tissues, such as epithelium, which shares the advantage of the random amplification and absence of the putative depletion without this risk of contamination. Nonetheless, positive selection of sSNVs has been identified in normal skin (Martincorena et al., 2015) and the extent to which this can be detrimental for their use as an outgroup tissue remains unknown.

The main caveats of our study are the reduced number of subject samples used and the lack of control individuals, for which paired tissue samples of similar quality are difficult to obtain. This prevented us from establishing that the uncovered load of somatic variants related to neuronal functions is linked to the disease. As we focused on brain tissues, translation coupled damage could explain the enrichment in the genes that are expressed in this organ, but all validated sSNVs were present in blood. Extending this experiment to more tissues and individuals will be key establish the role of somatic mutations in Parkinson disease. In addition to somatic mutations, we found that our cohort carried germline variants linked to the disease. However, these were sporadic cases, suggesting that germline mutations are not enough to cause the disease, and that the presence of somatic mutations might contribute as an additive factor that increases an individual’s susceptibility to the disease. Because of these complex interactions, and as these somatic variants could be also found in healthy controls, hundreds or thousands of individuals would be needed to perform association studies (a GWAS-like approach). Furthermore, somatic mutations increase with age, so an age-matched cohort would be essential to control for this effect.

We discarded variants when multiple individuals had reads supporting the same allele. This is because the probability of recurrent errors is much higher than the somatic mutation rate, especially given the number of divisions we can disentangle with bulk medium-coverage sequencing data. Also, this strategy assumes that the disease can be caused by a variety of mutations, instead of a few recurrent events, as it is a common disease with a low heritability component (Keller et al., 2012). However, germline variants are recurrent because they appear in more fragile or tolerated regions of the genome. Somatic mutations will surely be subject to the same processes when evaluated at a population level. These variants might then be selected for or against in different tissues, as shown for epithelia (Martincorena et al., 2015; Martincorena et al., 2018; Lee-Six et al., 2019; Moore et al., 2020; Yoshida et al., 2020), changing our expectations to find a given somatic variant in a particular tissue. Using sensitive and accurate sequencing techniques, such as NanoSeq (Abascal et al., 2021), will be fundamental to discover such somatic recurrent events. Finally, exome sequencing data is limited to a narrow set of regions of the genome. This limited our power to observe different cell lineages and their presence in each tissue or perform mutational signature deconvolution. Although exome variants can be more easily interpreted, the decreasing cost of sequencing and recent advances in contact maps (Lu et al., 2020) make finding and interpreting non-coding variants more accessible.

The somatic mutations we identified in Parkinson patients are promising candidates to contribute to the disease, as they affect genes involved in neuronal and axonal pathways and interact with genes associated with the disease. Together with previous studies, our results suggest an exciting new research path for the study of disease, especially complex disorders such as neurodegenerative diseases. This new evidence supports that not only germline point mutations, copy number variants, mitochondrial variants, and the environment are relevant, but also somatic mutations could contribute to Parkinson disease, probably by affecting the same key pathways. The study of somatic mutations in larger cohorts can help to identify the relevant molecular routes, helping us understand the disease and finding potential therapeutic targets.

## Methods

### Exome Sequencing

Tissue samples from cerebellum, neocortex, striatum and substantia nigra and blood were sourced from the HCB-IDIBAPS biobank. Blood samples were obtained from stored vials while brain samples were collected at autopsies, 6–18 h after death, and kept frozen at -80 °C. DNA extractions were carried out with the Qiagen DNeasy Blood & Tissue Kit. Genomic DNA samples were randomly fragmented into 150–200 bp length sequences. Adapters were ligated and the resulting templates were purified with AgencourtAMPure SPRI beads. Libraries were amplified by ligation-mediated polymerase chain reaction (LM-PCR). The Exon Focus SureSelect kit from Agilent was used to capture the exome and paired-end 100 bp sequencing was performed on an Illumina Hiseq2000 platform.

### Sequencing Data Processing

The resulting FASTQ files were mapped with BWA v0.7.8 mem (Li, 2013) to the human hs37d5 assembly. Lane-specific read groups were added with Picard Tools v1.95 (Broad Institute, 2013) AddOrReplaceReadGroups and bams were merged by sample with samtools v1.9 (Li et al., 2009). Read duplicates were removed with Picard Tools v1.95 MarkDuplicates REMOVE_DUPLICATES = true. Base quality score recalibration and indel realignment were applied following GATK’s best practices (DePristo et al., 2011) with GATK v3.6 (McKenna et al., 2010). Secondary alignments were also excluded with samtools view -F 256. Cram files including unmapped reads are available at ENA (European Nucleotide Archive) under the study accession number PRJEB43918. All the code used to process and generate data are available in https://github.com/ilobon/ParkinsonSomatic

### Germline Variants

Germline variants were called with GATK v3.6. First, GVCFs were obtained for each sample independently with -T HaplotypeCaller–emitRefConfidence GVCF. Then, all samples were genotyped together with -T GenotypeGVCFs and a standard hard filter was applied with -T VariantFiltration--filterExpression “QD < 2.0 || FS > 60.0 || MQ < 40.0 || MQRankSum < -12.5 || ReadPosRankSum < -8.0”. A PCA of the hard-filtered genotypes was performed with EIGENSOFT v7.2.1 (Price et al., 2006). Information on the called variants was annotated with SnpEff 4.3t and SnpSift 4.3t (Cingolani et al., 2012) and dbNSFP was used to add population frequencies, effect prediction and conservation scores. Overrepresentation enrichment analysis was performed with WebGestalt (Zhang et al., 2005) using the Gene Ontology (GO) database (Carbon et al., 2009) for molecular functions and genome protein-coding genes as background.

### Somatic Variant Calling

Somatic variants were called using HaplotypeCaller and VarScan 2 with lax parameters. Then, an extensive filtering strategy was applied to recover high confidence somatic SNVs. For HaplotypeCaller somatic variant calling, GVCFs per sample were obtained with GATK -T HaplotypeCaller -ploidy 10 -A StrandAlleleCountsBySample -- emitRefConfidence GVCF. Then, all GVCFs were genotyped together per chromosome with -T GenotypeGVCFs -L chr -ploidy 10 -A StrandAlleleCountsBySample to obtain somatic SNV and indel calls. For VarScan 2 (Koboldt et al., 2012) somatic variant calling, mpileup files were obtained with samtools mpileup per individual. Then, single nucleotide variants were called with VarScan v2.3.2 mpileup2snp with lax parameters: -min-coverage 1 -min-reads2 1 -p-value 1 -min-var-freq 0.000001 -output-vcf. Indels were called with mpileup2indel with the same parameters.

Depth of coverage files were obtained with GATK v3.6 DepthOfCoverage and GC content per target was calculated with GCContentByInterval. Then, CNVs were called jointly for all samples with XHMM v1.0 (Fromer et al., 2012) with standard parameters following its recommended best practices. Short tandem repeats in hs37d5 were determined with Tandem Repeats Finder v.4.09 (Benson, 1999) with parameters 2 7 7 80 10 12 500 -h. Homopolymers were then extracted based on their homogeneous repeat motif. The 1000GP strict mask FASTA files were obtained from the 1000GP FTP (ftp://ftp.1000genomes.ebi.ac.uk/vol1/ftp/release/20130502/supporting/accessible_genome_masks/) and were transformed into a BED file. BED files for WGAC segmental duplications, common dbSNP SNPs and mappability for 100mers for hg19—which shares coordinates with hs37d5—were obtained from the UCSC table browser (Karolchik et al., 2004). This information was annotated in the VCF files with BCFtools (Li et al., 2009). A panel of normals (PON) containing allele counts at each genomic position along 428 individuals sequenced at the Sanger Institute was used to identify recurrent errors.

### Somatic Variant Filtering Using COSMOS

We first defined a non-callable set of positions, including off-target calls as well as those overlapping with the 1000GP strict mask, the WCAG track, mappability lower than 1, by homopolymers or within 5 base pairs of indels. Additional sources of artefacts were identified in the lax call sets by manual inspection of the raw data with IGV (Robinson et al., 2011), oftentimes evidenced by recurrent patterns in different individuals. To filter calls we wrote a python module, COSMOS (Combined Or Single sample MOSaicism detection), which is available in https://github.com/ilobon/COSMOS. The first step is to annotate all the information necessary to classify true and false calls. Then, variants are filtered according to the user indicated features and thresholds. Two different filtering approaches can be used. When multiple samples—tissues or replicates—from the same individual are available, their combined information can be used to filter calls more accurately. This is, since true and false variants have partially overlapping values in the determining features, even true somatic mutations can fail some tests. Hence, we can take advantage of the multiple samples, and assuming the variants are present in more than one of them, require than at least *n* samples pass each filter, allowing a different combination of passing samples at each feature (-combined TRUE-nSamplesPerInd *n*). COSMOS can also be used to filter each sample individually (-combined FALSE), or output the result of both approaches (-combined BOTH-nSamplesPerInd *n*).

To filter our Parkinson exome data, we used both approaches, requiring at each position either a single sample passing all filters or any combination of four out of the five tissues passing at every filter. The parameters we used were *-c BOTH -ns 4 -ad 2 -adss 3 -vaf 0.5 -dp1 20 -dp2 100 -sr 2 -pr 4 -sp 0.05 -b 0.05 -nrl 4 -hap 4 -cnv NO -pir 4,4 -vafq 0.4 -clip 0.9 -mq 0.05 -mm 0.05 -pon 0.05*. In short, this requires at least 2 reads supporting the alternative allele, or 3 for the single sample approach; a variant allele frequency lower than 0.5; a depth between 20 and 100; a strand count ratio <2; a pair count ratio <4; non-significant p-values for a Poisson test of strand counts; a significant binomial test of allele counts; at most 4 variants within read length; less than 4 haplotypes; absence of XHMM CNV call; PIR score of 4, meaning that position in reads of each allele is not biased; a variant allele frequency from direct bam read counts <0.4; an allele clipping ratio difference <0.9; non-significant Mann-Whitney tests for mapping quality an number of mismatches per allele and a significant beta-binomial test for allele counts compared to the PON. More features and different thresholds can be used for filtering depending on each dataset characteristics such as depth.

### Amplicon-Based Deep Sequencing

To validate the high confidence variants, we performed amplicon-based deep sequencing (ADS) by using rhAmpSeq technology (IDT, Coralville, United States). This is a multiplexed strategy that amplifies all selected positions in a unified reaction. We then sequenced the amplified material from each sample in a MiSeq v3 run obtaining paired end 300bp reads to a mean coverage ∼18,000X. Data were processed the same way as the exome files. To consider a variant as a validated sSNV in each sample, we required that (1) it was the second most common allele in that sample and (2) its VAF was higher than the mean + 2 standard deviations of all other individuals’ samples VAFs with sufficient coverage (defined as not lower than the mean–1 standard deviation). We additionally classified variants passing these filters in multiple tissues of an individual and with VAFs > 30% in all of them as germline heterozygous variants (Supplementary material).

## Data Availability

The datasets presented in this study can be found in online repositories. The names of the repository/repositories and accession number(s) can be found below: https://www.ebi.ac.uk/ena/browser/view/PRJEB43918.

## Abbreviations

CNV, Copy number variant; COSMOS, Combined Or Single sample MOSaicism detection (method); PD, Parkinson disease; PIR, Position-in-reads bias score; PON, ‘Panel of normals’; SBS, Single base signatures; sSNV, Somatic single nucleotide variant; VAF, Variant allele frequency.
