# Supplementary material for: Somatic Mutations Detected in Parkinson Disease Could Affect Genes With a Role in Synaptic and Neuronal Processes
Source: Front Aging. 2022 Apr 28;3:851039. doi: 10.3389/fragi.2022.851039 (PMC9261316; doi:10.3389/fragi.2022.851039)
Supplement: Supplementary file 1 [file DataSheet2.pdf]

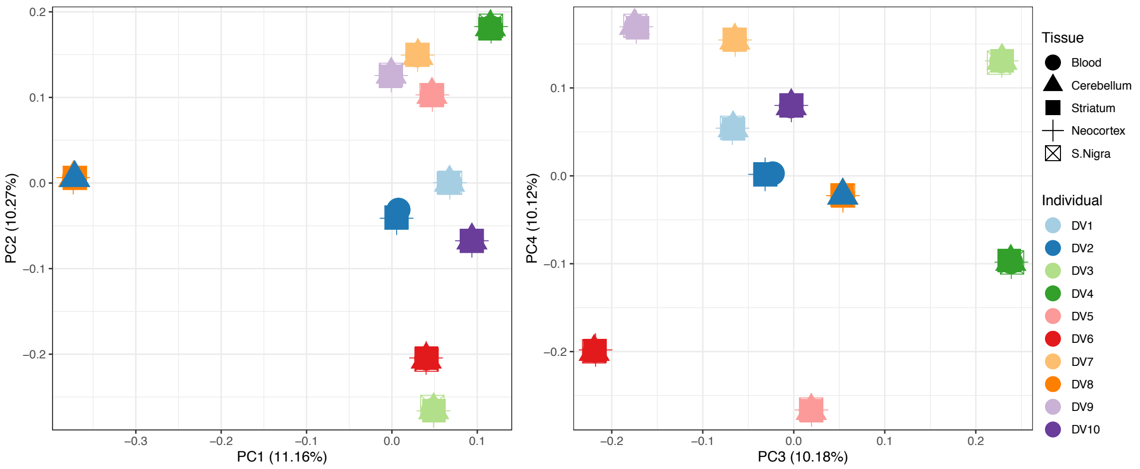


**Supplementary figure 1. Germline variants PCA.** First four principal components (PC) with the amount of variance explained by each of them in parenthesis. DV2's cerebellum clusters with DV8's tissues and its blood show lower affinity to the other tissues than in the other individuals.

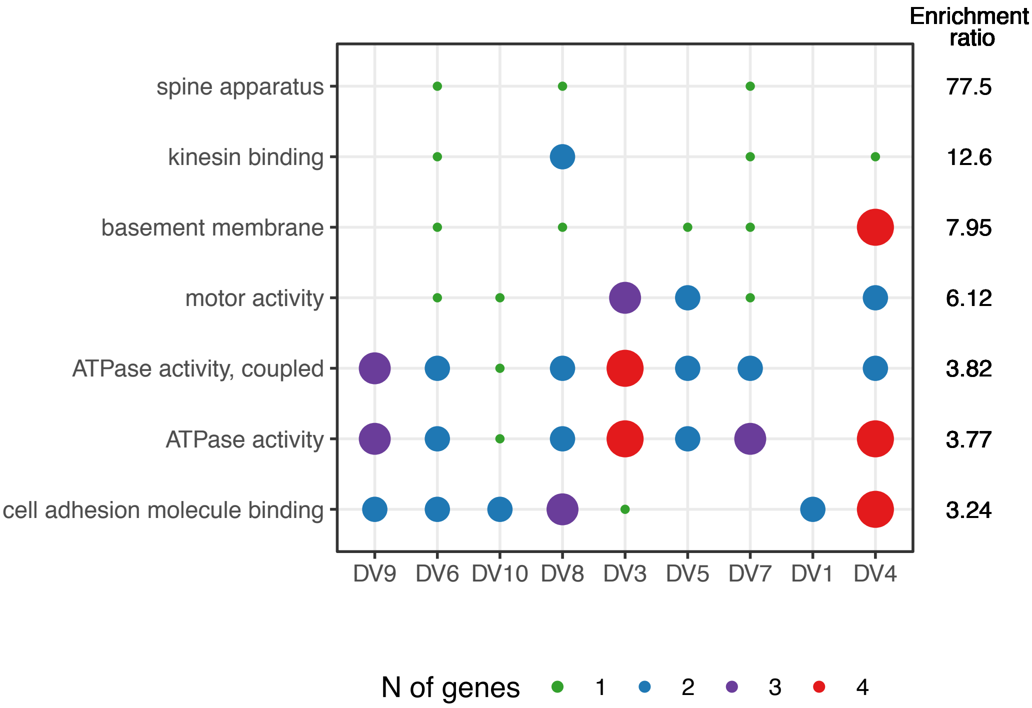


**Supplementary figure 2. Enriched terms in deleterious variants**. Overrepresentation analysis results for molecular function and cellular compartment in genes carrying germline variants predicted as deleterious and with low frequency in the reference Spanish population (1000GP IBS). Dot size and color represent the number of genes associated with each term each individual carries. Enrichment ratios are annotated on the right side, and all terms are significant (FDR $\leq$ 0.05). Individuals are ordered by ascending age at death.

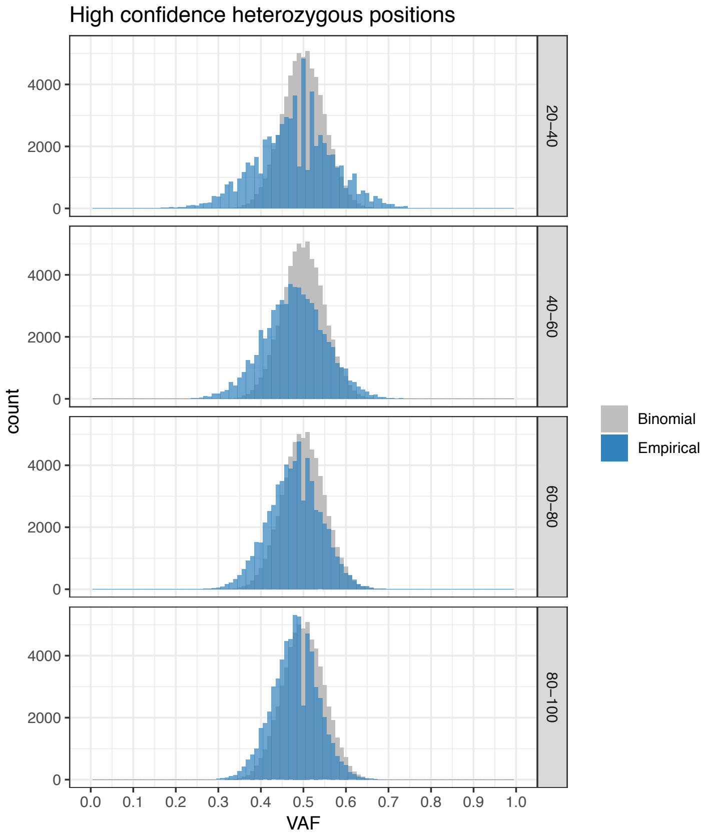


**Supplementary figure 3. Variant allele frequency dispersion gets smaller at higher depths.** VAF was stratified by coverage window (Y-axis facets). Random samples were obtained from each bin with a size equal to that of the smaller bin (in blue). A binomial distribution with p=0.5 is shown in grey for reference.

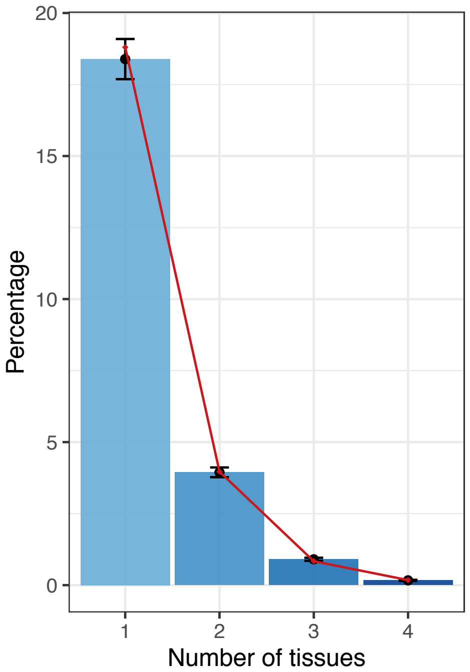


**Supplementary figure 4. Power to detect heterozygous variants by number of tissues.** Median percentages of high-confidence heterozygous positions that would be classified as not heterozygous because their binomial test in all the 1, 2, 3, or 4 tissues tested is significant (p-value<0.05). The red line shows a fitted exponential model. Black bars show the standard deviation. For each number of tissues, all possible comparisons were performed.

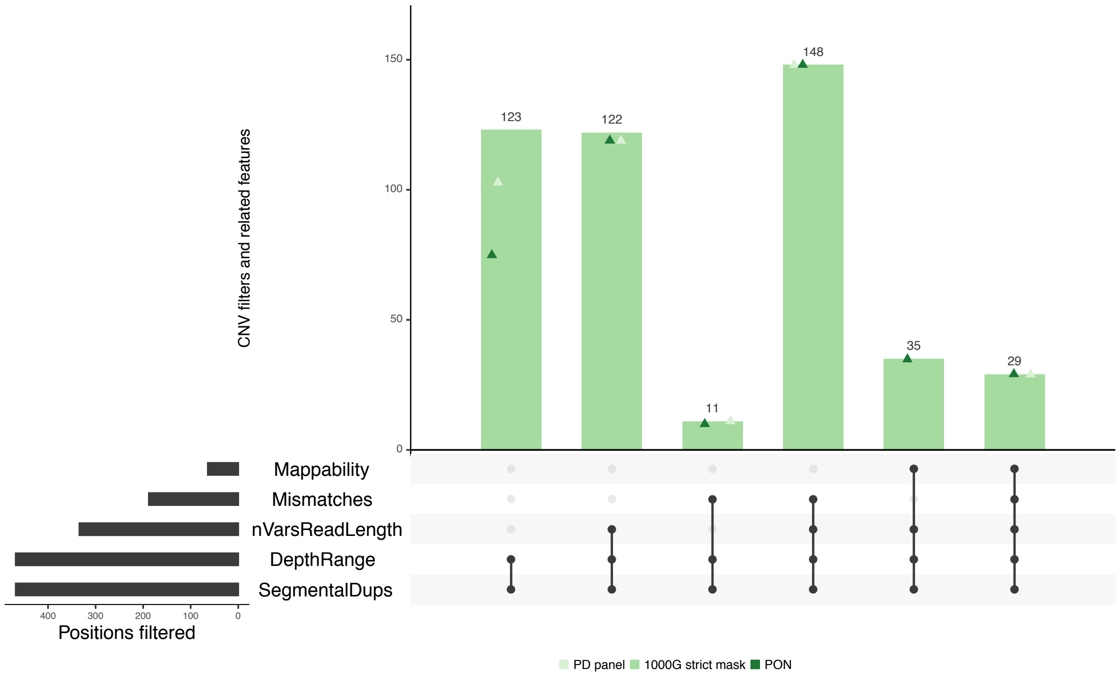


**Supplementary figure 5. Intersection of additional filters with the main CNV filters.** Intersection of CNV related filters in calls that are on target, not germline heterozygous, fail depth range and segmental duplications with PD panel and PON (light and dark green triangles, respectively) and variants present in the 1000GP strict mask filled in green.

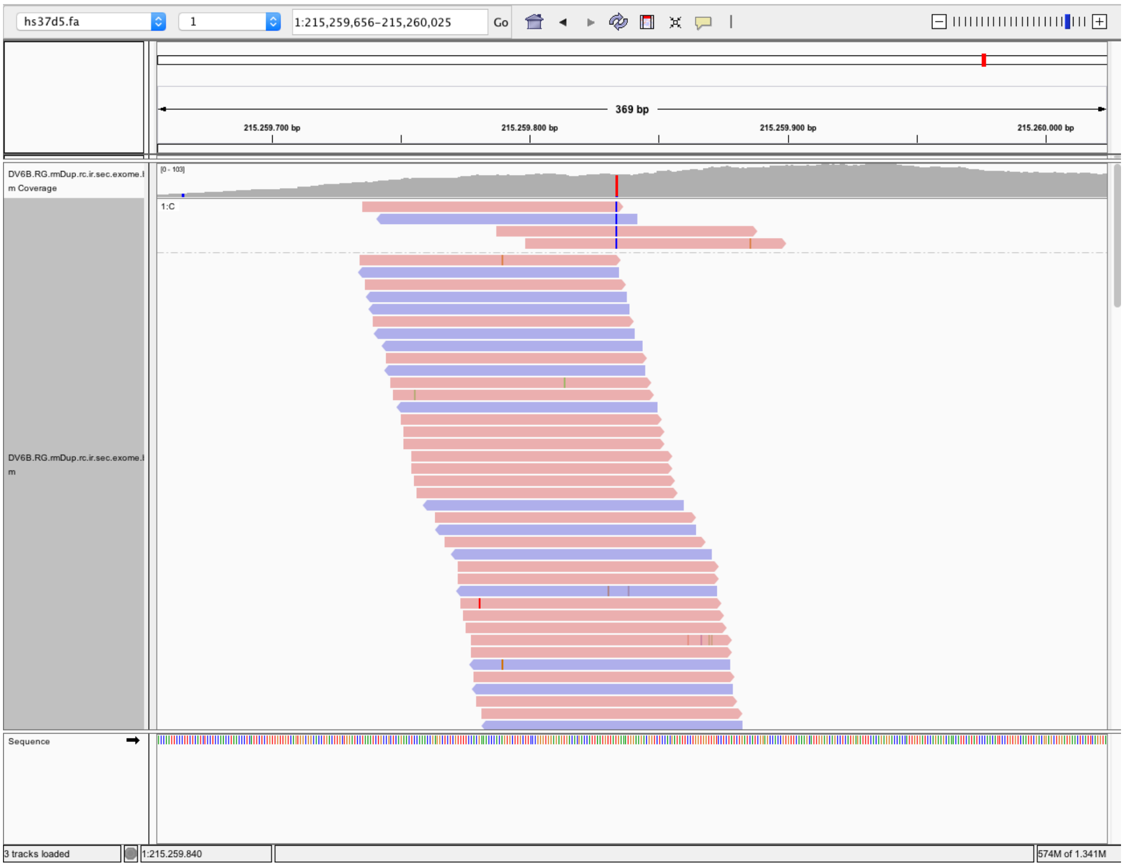


**Supplementary figure 6. Example of a high confidence sSNV.** IGV screenshot showing a tier 1 variant, for which only DV6's blood had any reads supporting the alternative allele. Reads aligned to the region do not look particularly noisy, and alternative allele supporting reads look unbiased. Vertical bars on top of the plot indicate the coverage per base pair, and the colored bar shows the variant position and allele proportions. Horizontal pink and blue lines denote forward and reverse reads, respectively, aligned to the region. Colored vertical bars within the reads indicate variants, with color depending on the alleles: A (green), C (blue), G (brown), and T (red).

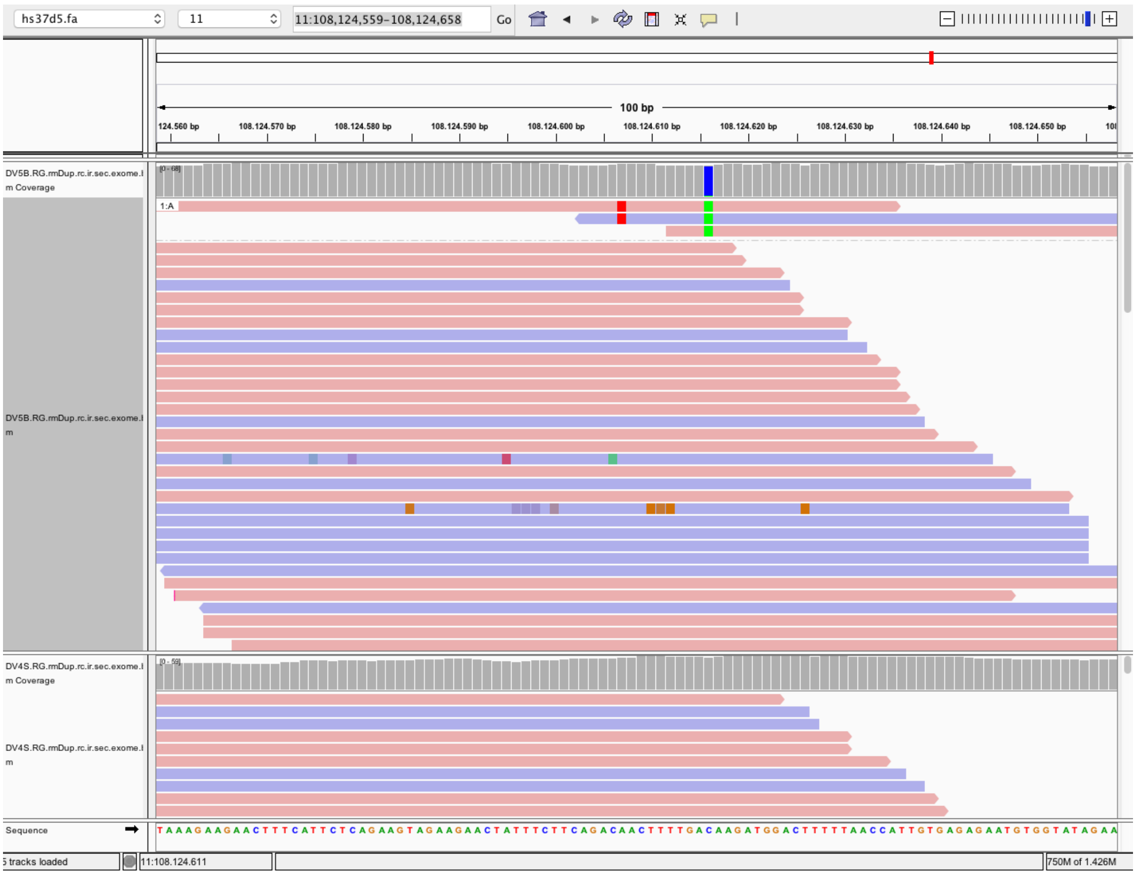


**Supplementary figure 7. Example of a low confidence sSNV.** IGV screenshot showing a tier 3 variant called in DV5's blood. Not only are noisy reads aligned to the region, but two of the reads supporting the alternative allele are in phase with another variant which was not present in any reference supporting reads, suggesting reads are misaligned. Vertical bars on top of the plot indicate the coverage per base pair, and the colored bar shows the variant position and allele proportions. Horizontal pink and blue lines denote forward and reverse reads, respectively, aligned to the region. Colored vertical bars within the reads indicate variants, with color depending on the alleles: A (green), C (blue), G (brown), and T (red).

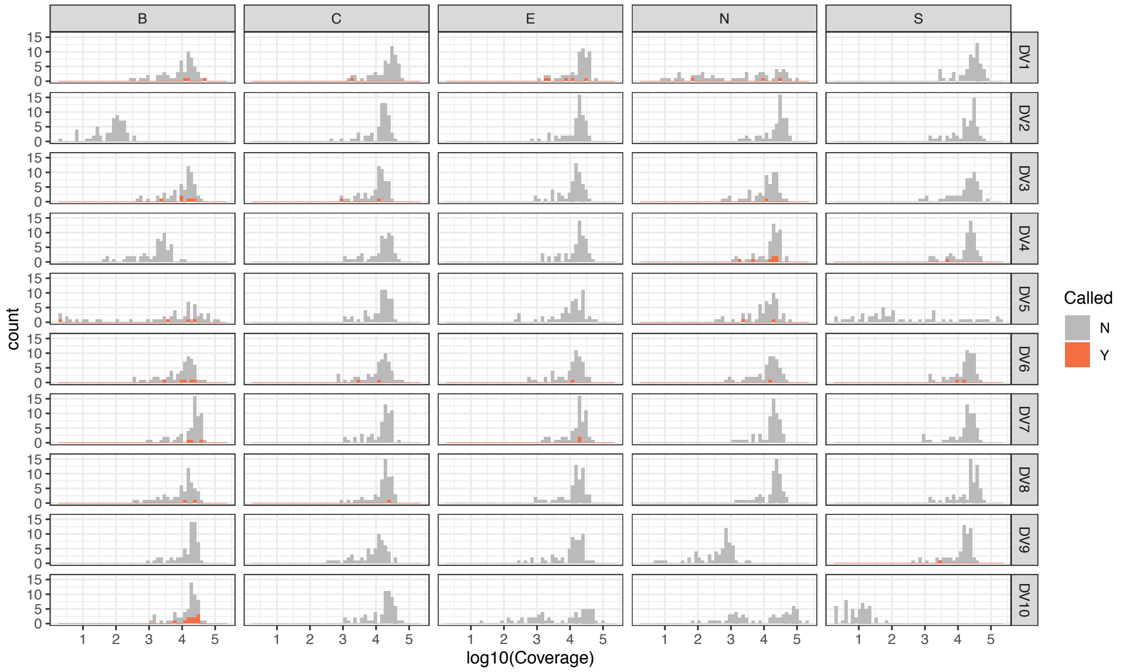


**Supplementary figure 8. Amplicon sequencing coverage.** Distribution of coverage at all positions per tissue and individual, with the coverage at positions called in the exome sequencing data of that sample in red.

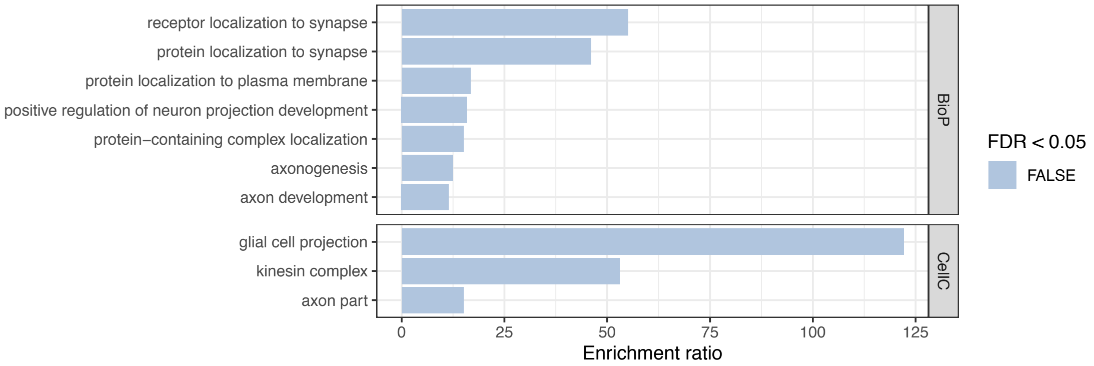


**Supplementary figure 9. Somatic variants enrichment analysis.** Overrepresentation enrichment analysis for biological process, cellular component, and molecular function with protein coding genes as background. Only the 15 validated brain variants that are nonsynonymous, affecting splicing consensus or a UTR region were used. All the terms are non-significant (FDR > 0.05).

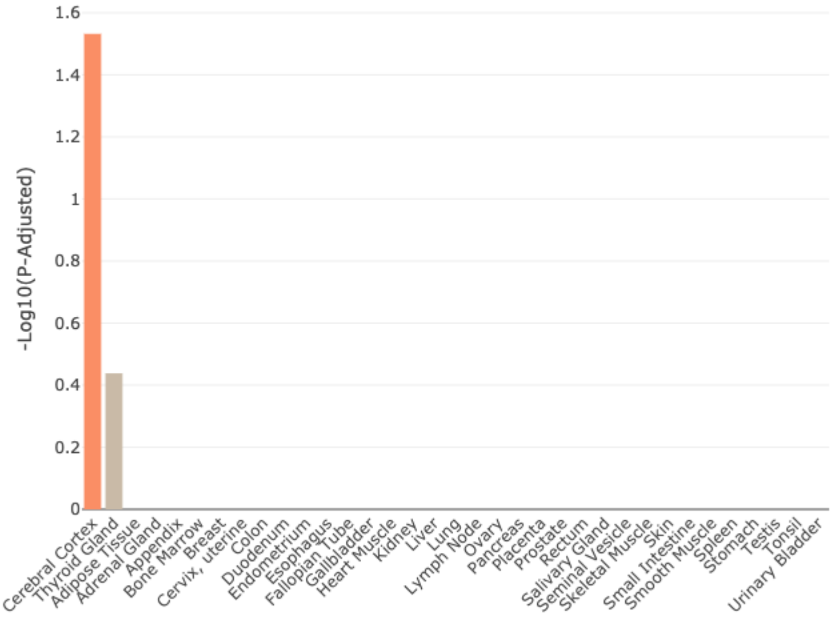


**Supplementary figure 10. Somatic variants tissue enrichment analysis.** Tissue enrichment analysis of the 15 validated variants which are in brain and are nonsynonymous or affect consensus splice sites.

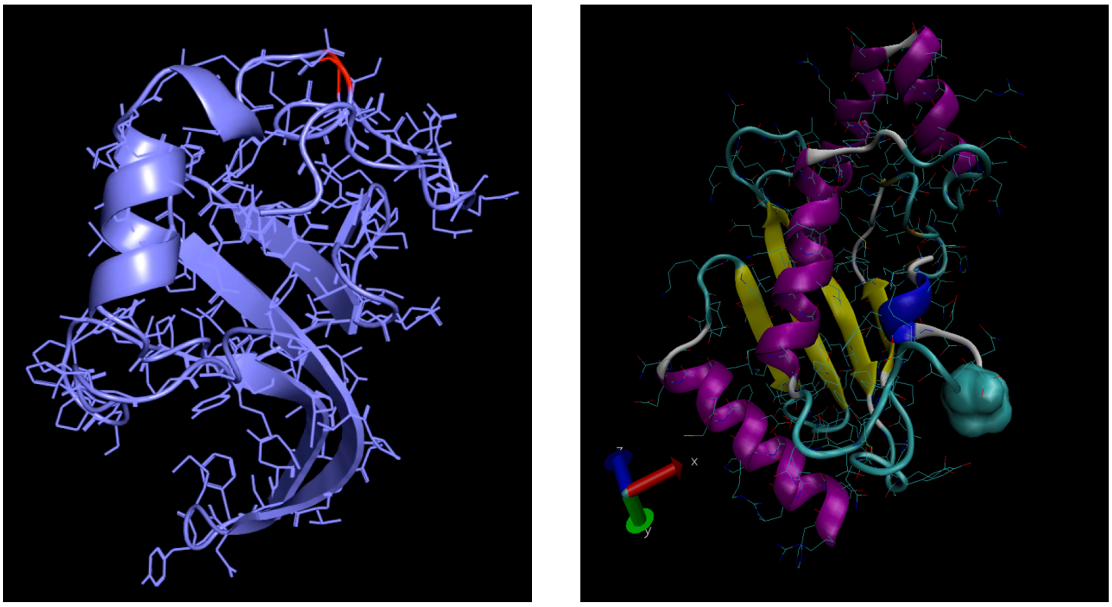

**Supplementary figure 11. Protein structures. A**. Structure predicted for the C-terminal domain of KIF5A, showing the secondary structures and amino acids, with the reference version of the mutated amino acid marked in red. **B.** Structure predicted for the active site and surrounding region of UBE2U, colored according to the secondary structure. The mutated amino acid can be recognized from its globular Surf shape.
