# Supplementary material for: Somatic Mutations Detected in Parkinson Disease Could Affect Genes With a Role in Synaptic and Neuronal Processes
Source: Front Aging. 2022 Apr 28;3:851039. doi: 10.3389/fragi.2022.851039 (PMC9261316; doi:10.3389/fragi.2022.851039)

Supplementary File Legend

Amplicon sequencing validation figures 1-57. The validation results for each candidate position are shown in each page (1-57). Variant allele frequency (VAF) and coverage of the amplicon sequencing data is shown for every sample. VAF is coloured by whether the candidate variant was called in the WES data. Coverage is coloured to indicate the frequency ranking of the candidate alternative allele. If the alternative allele was the 3rd or 4th most frequent allele, the candidate mutation was considered as not validated (see *VWA5B1* for an example). If it was the most common one (hence with a VAF >50%) it was considered a germline variant (see *EDAR* for an example). Candidates which were the 2nd most common allele but whose VAF was not significantly higher than in the other individuals were considered as false positives (see *GALNT5* for an example).

1.

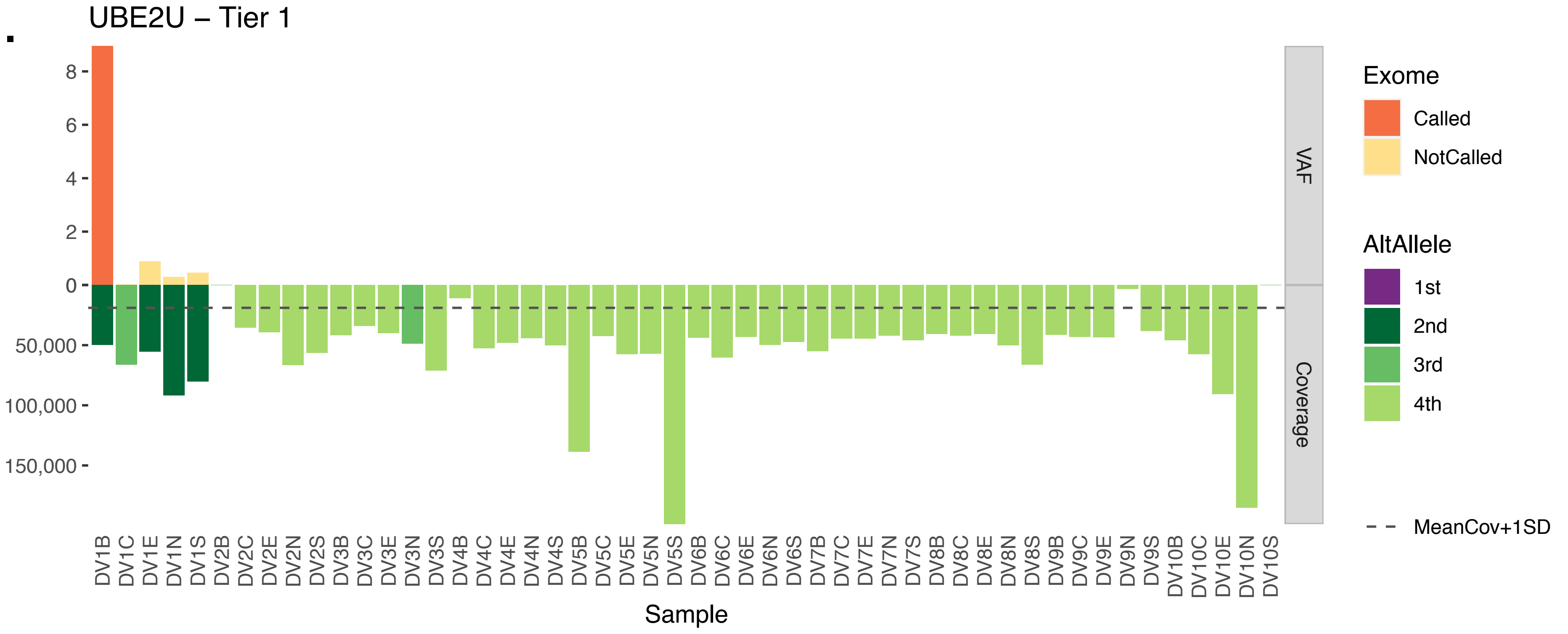
UBE2U – Tier 1
8
6
4
2
0
50,000
100,000
150,000
VAF
Coverage
Exome
Called
NotCalled
AltAllele
1st
2nd
3rd
4th
MeanCov+1SD
DV1B DV1C DV1E DV1N DV1S DV2B DV2C DV2E DV2N DV2S DV3B DV3C DV3E DV3N DV3S DV4B DV4C DV4E DV4N DV4S DV5B DV5C DV5E DV5N DV5S DV6B DV6C DV6E DV6N DV6S DV7B DV7C DV7E DV7N DV7S DV8B DV8C DV8E DV8N DV8S DV9B DV9C DV9E DV9N DV9S DV10B DV10C DV10E DV10N DV10S
Sample

2.

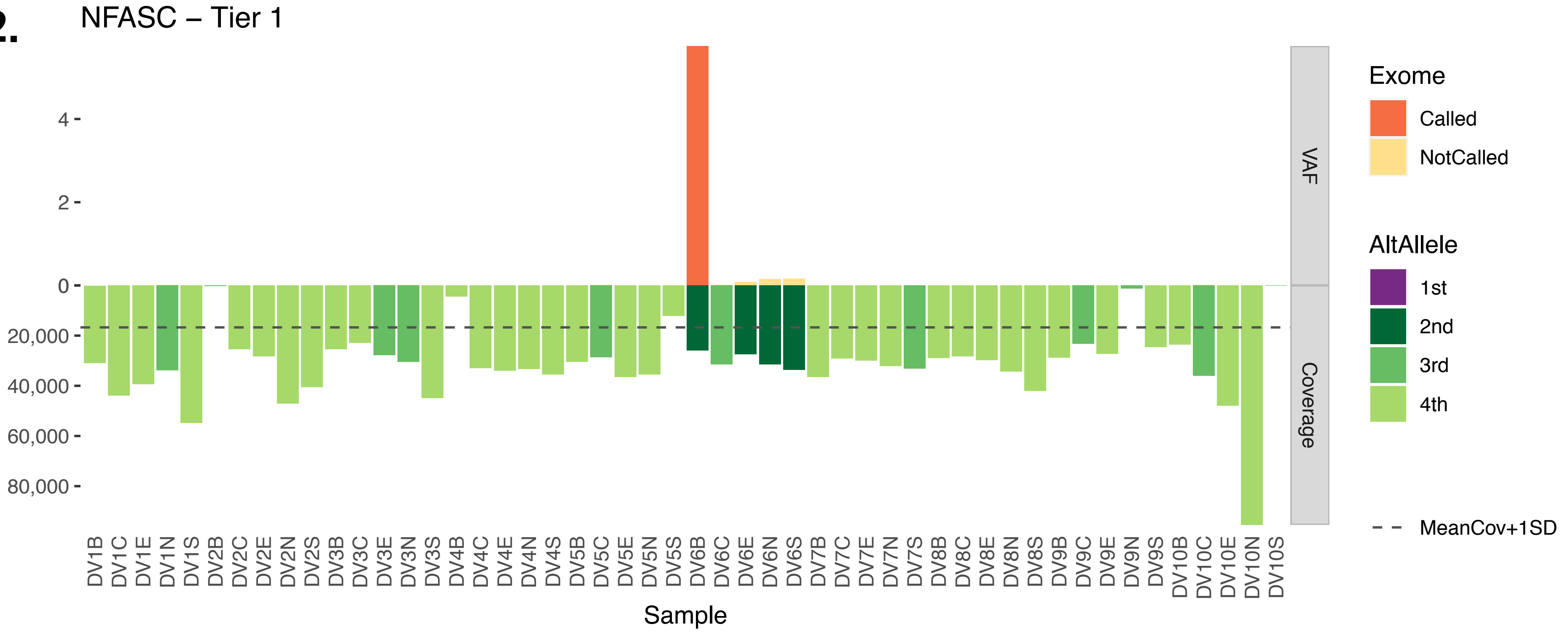
NFASC – Tier 1
4
2
0
20,000
40,000
60,000
80,000
VAF
Coverage
Exome
Called
NotCalled
AltAllele
1st
2nd
3rd
4th
MeanCov+1SD
DV1B
DV1C
DV1E
DV1N
DV1S
DV2B
DV2C
DV2E
DV2N
DV2S
DV3B
DV3C
DV3E
DV3N
DV3S
DV4B
DV4C
DV4E
DV4N
DV4S
DV5B
DV5C
DV5E
DV5N
DV5S
DV6B
DV6C
DV6E
DV6N
DV6S
DV7B
DV7C
DV7E
DV7N
DV7S
DV8B
DV8C
DV8E
DV8N
DV8S
DV9B
DV9C
DV9E
DV9N
DV9S
DV10B
DV10C
DV10E
DV10N
DV10S
Sample

3.

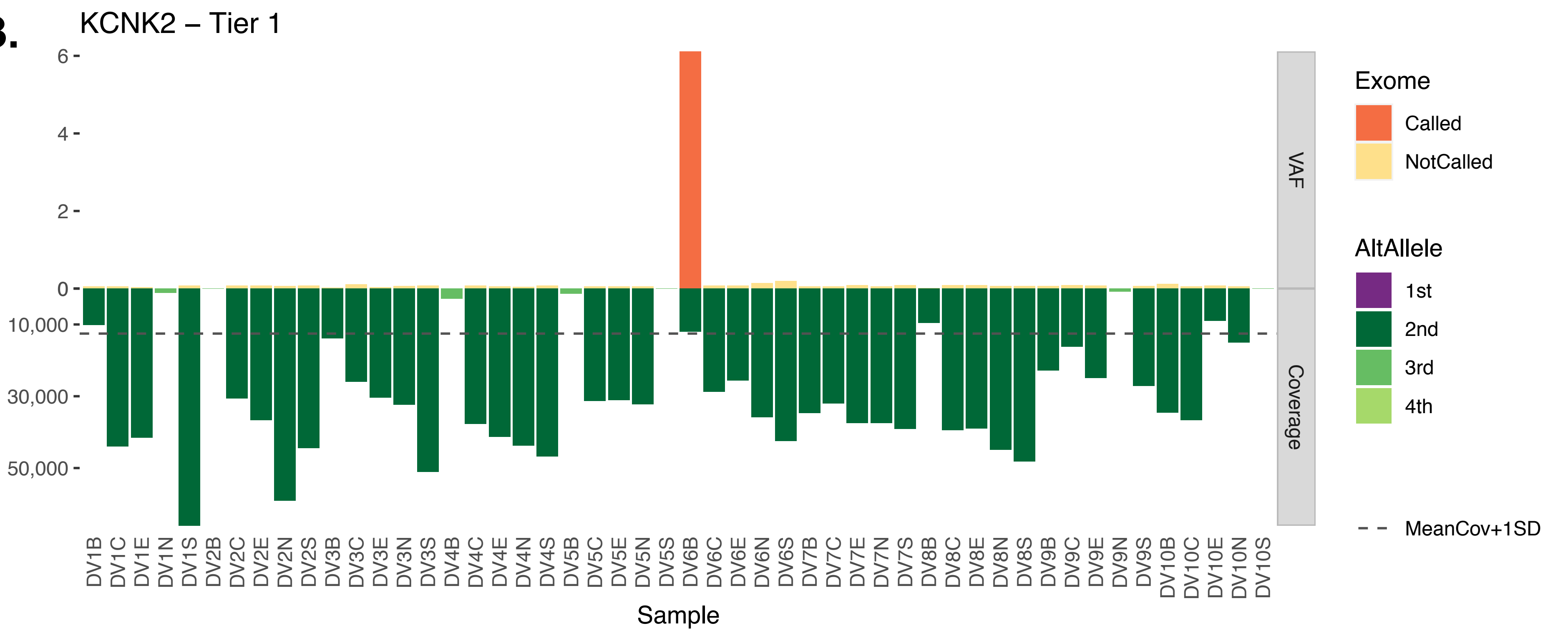
KCNK2 – Tier 1
6
4
2
0
10,000
30,000
50,000
VAF
Coverage
Exome
Called
NotCalled
AltAllele
1st
2nd
3rd
4th
MeanCov+1SD
DV1B DV1C DV1E DV1N DV1S DV2B DV2C DV2E DV2N DV2S DV3B DV3C DV3E DV3N DV3S DV4B DV4C DV4E DV4N DV4S DV5B DV5C DV5E DV5N DV5S DV6B DV6C DV6E DV6N DV6S DV7B DV7C DV7E DV7N DV7S DV8B DV8C DV8E DV8N DV8S DV9B DV9C DV9E DV9N DV9S DV10B DV10C DV10E DV10N DV10S
Sample

4.

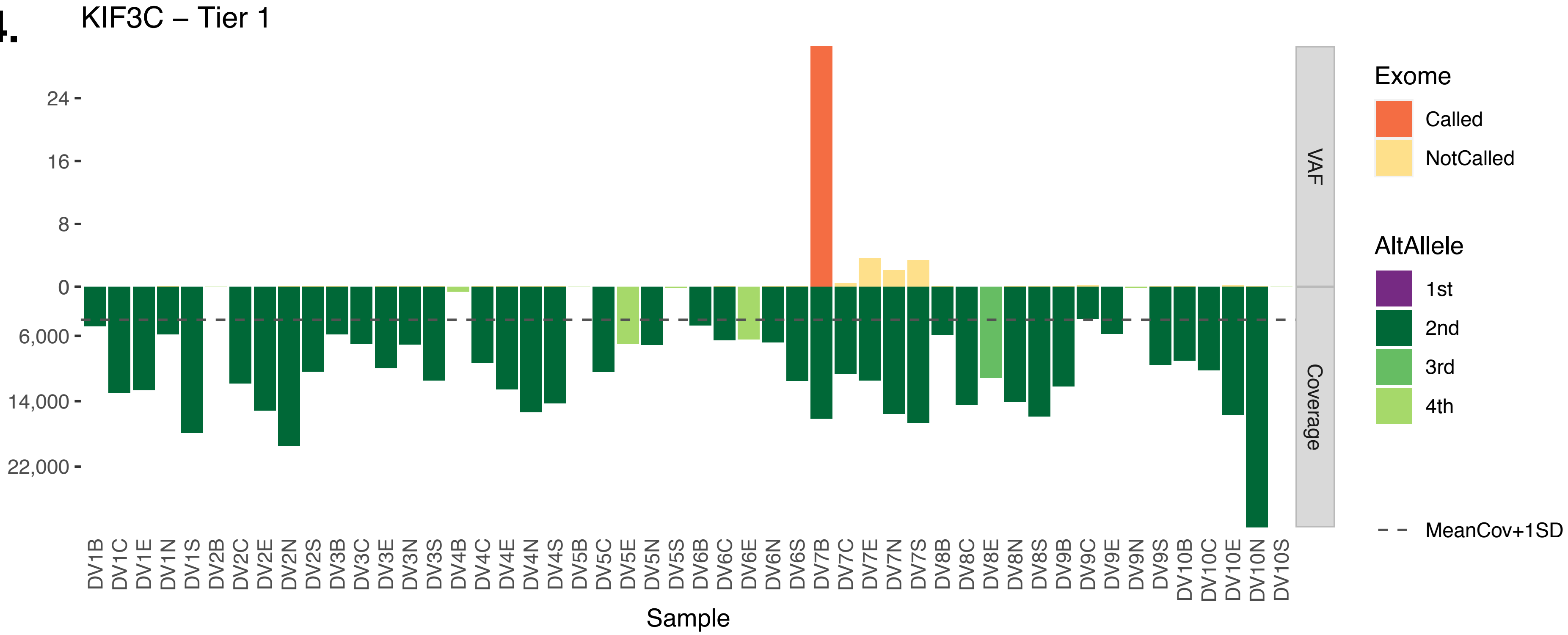

KIF3C – Tier 1
24
16
8
0
6,000
14,000
22,000
VAF
Coverage
Exome
Called
NotCalled
AltAllele
1st
2nd
3rd
4th
MeanCov+1SD
DV1B
DV1C
DV1E
DV1N
DV1S
DV2B
DV2C
DV2E
DV2N
DV2S
DV3B
DV3C
DV3E
DV3N
DV3S
DV4B
DV4C
DV4E
DV4N
DV4S
DV5B
DV5C
DV5E
DV5N
DV5S
DV6B
DV6C
DV6E
DV6N
DV6S
DV7B
DV7C
DV7E
DV7N
DV7S
DV8B
DV8C
DV8E
DV8N
DV8S
DV9B
DV9C
DV9E
DV9N
DV9S
DV10B
DV10C
DV10E
DV10N
DV10S
Sample

5.

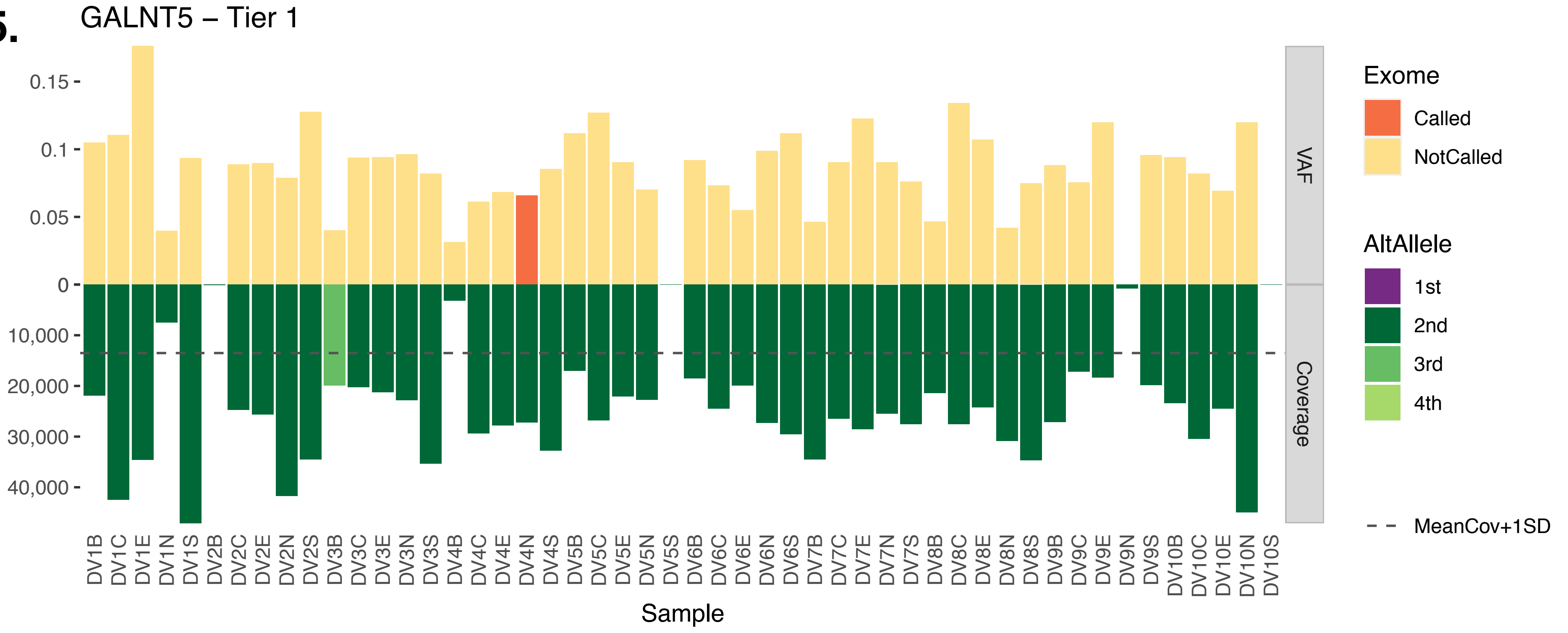
GALNT5 – Tier 1
0.15
0.1
0.05
0
10,000
20,000
30,000
40,000
VAF
Coverage
Exome
Called
NotCalled
AltAllele
1st
2nd
3rd
4th
MeanCov+1SD
DV1B DV1C DV1E DV1N DV1S DV2B DV2C DV2E DV2N DV2S DV3B DV3C DV3E DV3N DV3S DV4B DV4C DV4E DV4N DV4S DV5B DV5C DV5E DV5N DV5S DV6B DV6C DV6E DV6N DV6S DV7B DV7C DV7E DV7N DV7S DV8B DV8C DV8E DV8N DV8S DV9B DV9C DV9E DV9N DV9S DV10B DV10C DV10E DV10N DV10S
Sample

6.

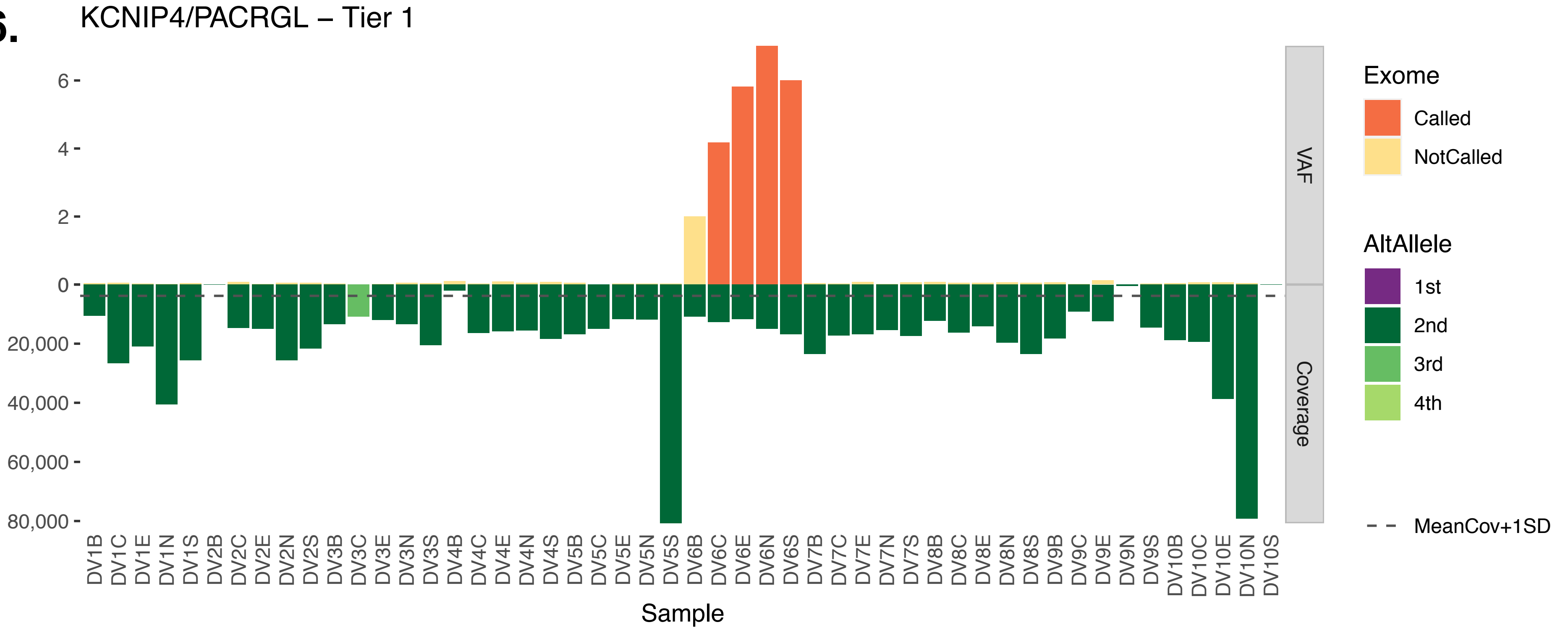

KCNIP4/PACRGL – Tier 1
6
4
2
0
20,000
40,000
60,000
80,000
VAF
Coverage
DV1B DV1C DV1E DV1N DV1S DV2B DV2C DV2E DV2N DV2S DV3B DV3C DV3E DV3N DV3S DV4B DV4C DV4E DV4N DV4S DV5B DV5C DV5E DV5N DV5S DV6B DV6C DV6E DV6N DV6S DV7B DV7C DV7E DV7N DV7S DV8B DV8C DV8E DV8N DV8S DV9B DV9C DV9E DV9N DV9S DV10B DV10C DV10E DV10N DV10S
Sample
Exome
Called
NotCalled
AltAllele
1st
2nd
3rd
4th
MeanCov+1SD

7.

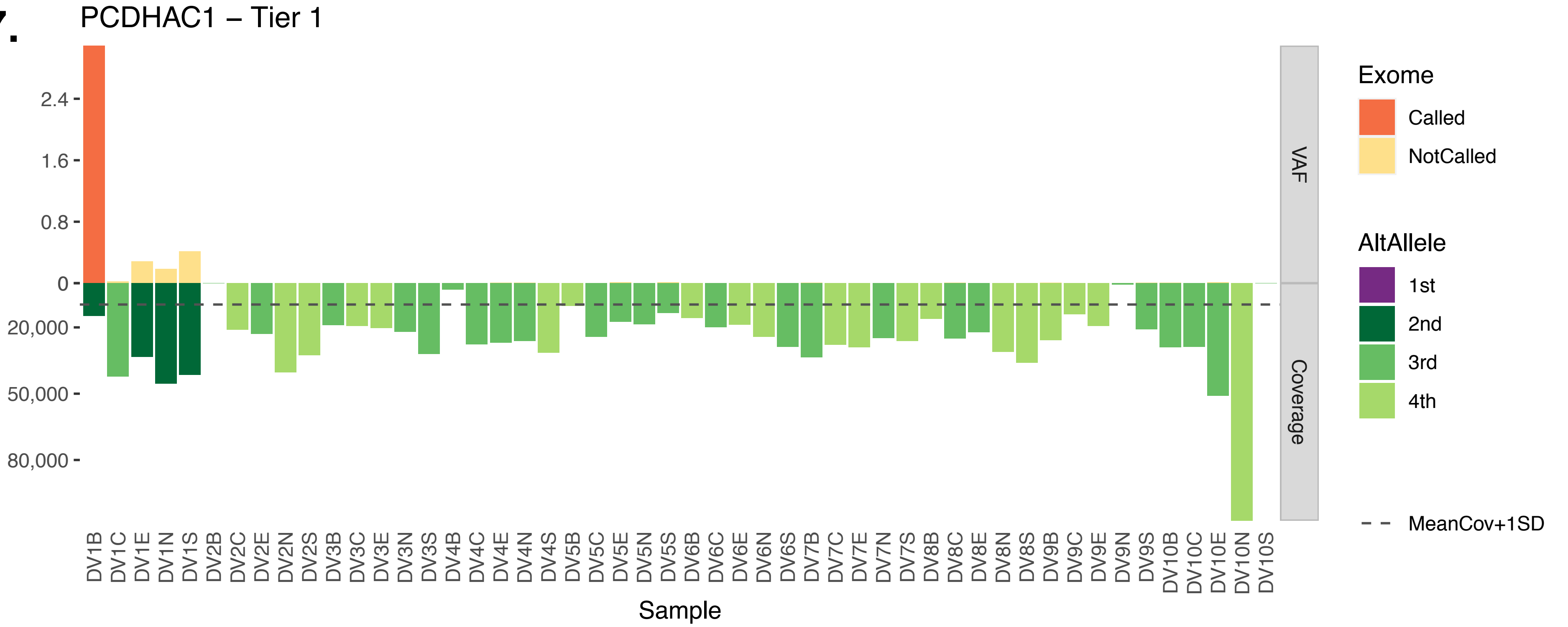

PCDHAC1 – Tier 1
2.4
1.6
0.8
0
20,000
50,000
80,000
VAF
Coverage
Sample
DV1B
DV1C
DV1E
DV1N
DV1S
DV2B
DV2C
DV2E
DV2N
DV2S
DV3B
DV3C
DV3E
DV3N
DV3S
DV4B
DV4C
DV4E
DV4N
DV4S
DV5B
DV5C
DV5E
DV5N
DV5S
DV6B
DV6C
DV6E
DV6N
DV6S
DV7B
DV7C
DV7E
DV7N
DV7S
DV8B
DV8C
DV8E
DV8N
DV8S
DV9B
DV9C
DV9E
DV9N
DV9S
DV10B
DV10C
DV10E
DV10N
DV10S
Exome
Called
NotCalled
AltAllele
1st
2nd
3rd
4th
MeanCov+1SD

8.

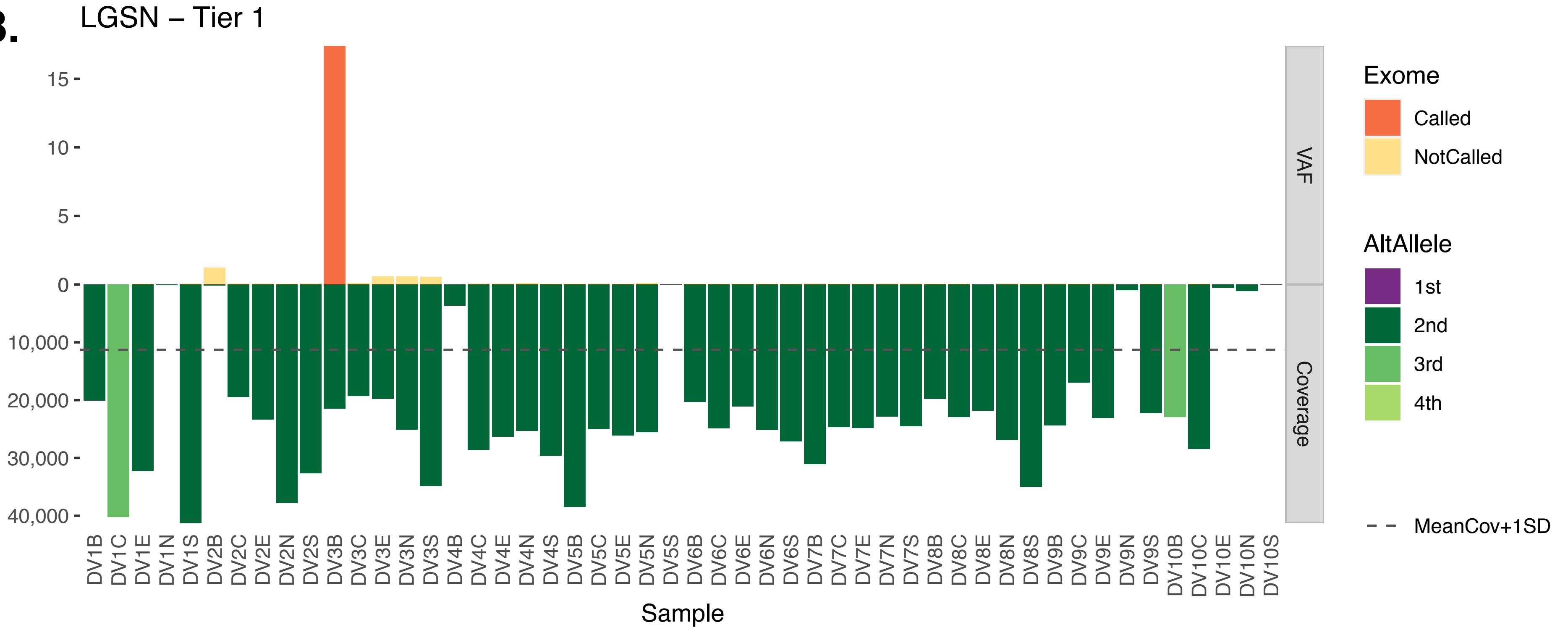

LGSN – Tier 1
15
10
5
0
10,000
20,000
30,000
40,000
VAF
Coverage
Exome
Called
NotCalled
AltAllele
1st
2nd
3rd
4th
MeanCov+1SD
DV1B
DV1C
DV1E
DV1N
DV1S
DV2B
DV2C
DV2E
DV2N
DV2S
DV3B
DV3C
DV3E
DV3N
DV3S
DV4B
DV4C
DV4E
DV4N
DV4S
DV5B
DV5C
DV5E
DV5N
DV5S
DV6B
DV6C
DV6E
DV6N
DV6S
DV7B
DV7C
DV7E
DV7N
DV7S
DV8B
DV8C
DV8E
DV8N
DV8S
DV9B
DV9C
DV9E
DV9N
DV9S
DV10B
DV10C
DV10E
DV10N
DV10S
Sample

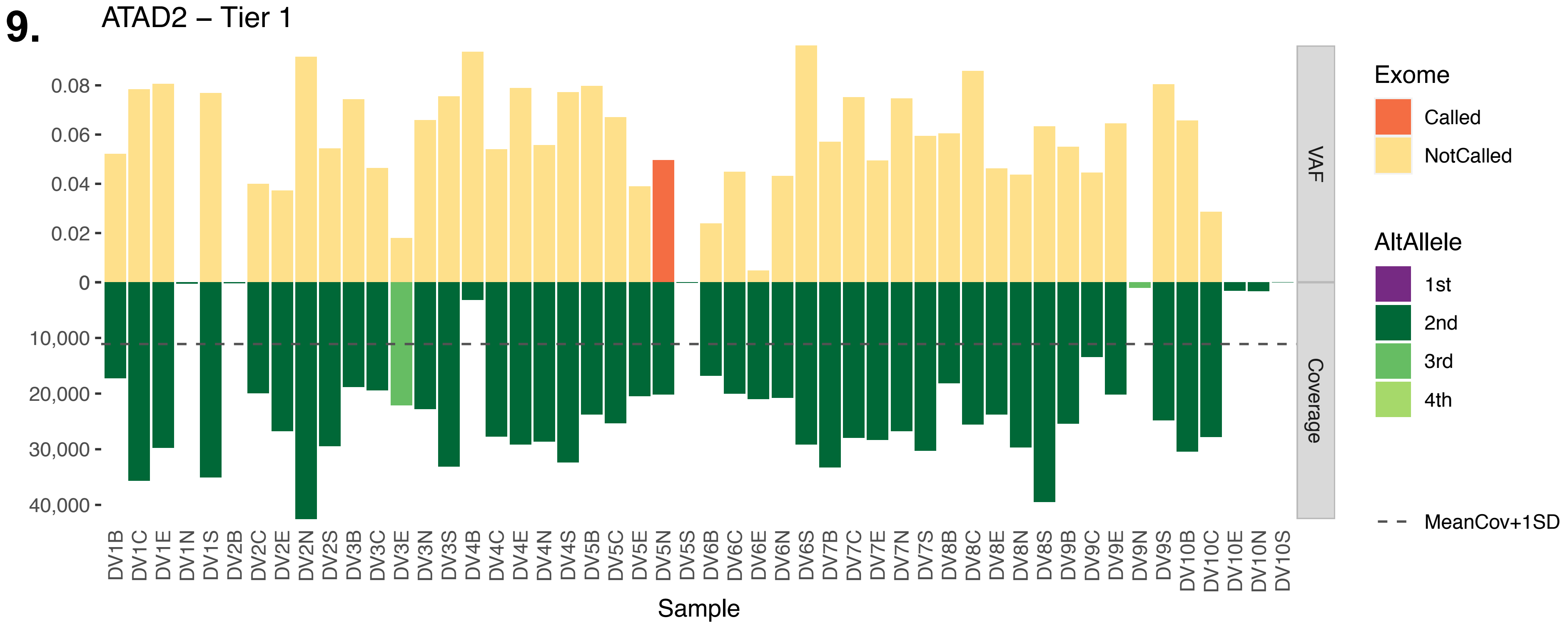

9.
ATAD2 – Tier 1
0.08
0.06
0.04
0.02
0
10,000
20,000
30,000
40,000
VAF
Coverage
Exome
Called
NotCalled
AltAllele
1st
2nd
3rd
4th
MeanCov+1SD
DV1B DV1C DV1E DV1N DV1S DV2B DV2C DV2E DV2N DV2S DV3B DV3C DV3E DV3N DV3S DV4B DV4C DV4E DV4N DV4S DV5B DV5C DV5E DV5N DV5S DV6B DV6C DV6E DV6N DV6S DV7B DV7C DV7E DV7N DV7S DV8B DV8C DV8E DV8N DV8S DV9B DV9C DV9E DV9N DV9S DV10B DV10C DV10E DV10N DV10S
Sample

10. NTRK2 – Tier 1

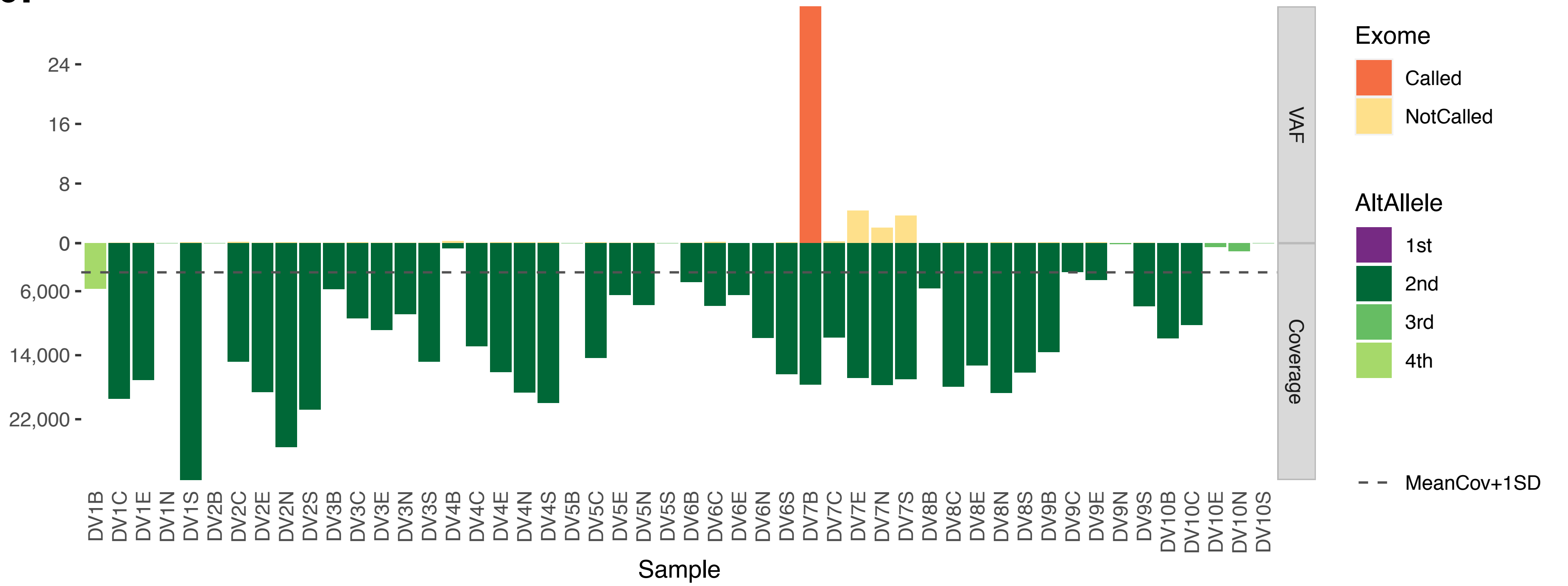

11.

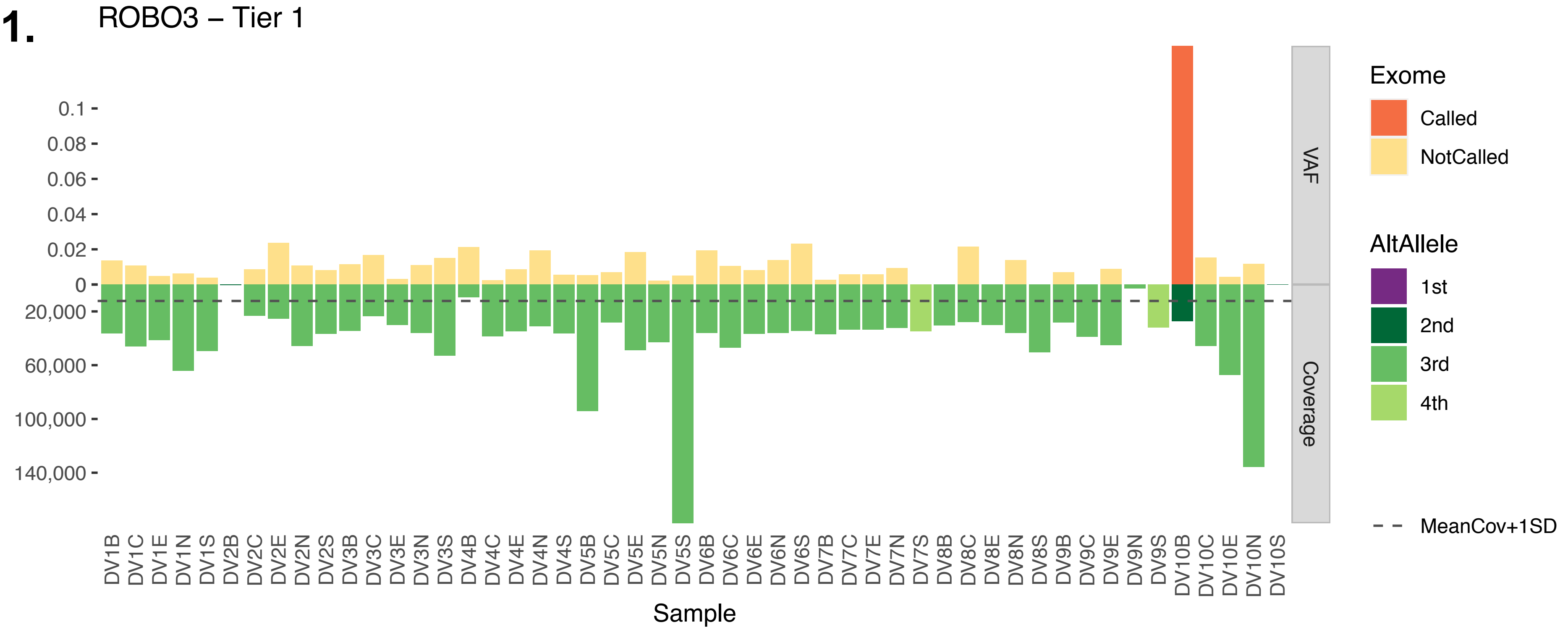
ROBO3 – Tier 1
0.1
0.08
0.06
0.04
0.02
0
20,000
60,000
100,000
140,000
VAF
Coverage
DV1B DV1C DV1E DV1N DV1S DV2B DV2C DV2E DV2N DV2S DV3B DV3C DV3E DV3N DV3S DV4B DV4C DV4E DV4N DV4S DV5B DV5C DV5E DV5N DV5S DV6B DV6C DV6E DV6N DV6S DV7B DV7C DV7E DV7N DV7S DV8B DV8C DV8E DV8N DV8S DV9B DV9C DV9E DV9N DV9S DV10B DV10C DV10E DV10N DV10S
Sample
Exome
Called
NotCalled
AltAllele
1st
2nd
3rd
4th
MeanCov+1SD

12.

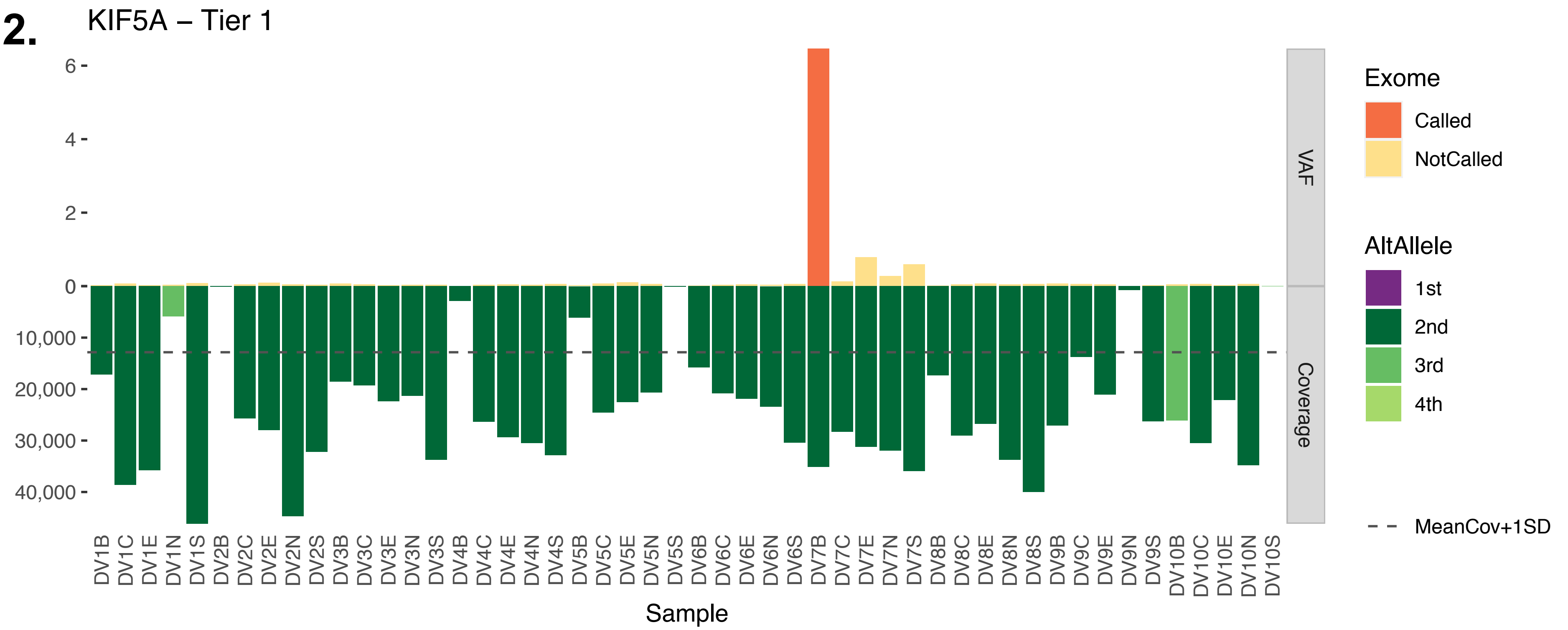

KIF5A – Tier 1
6
4
2
0
10,000
20,000
30,000
40,000
VAF
Coverage
Exome
Called
NotCalled
AltAllele
1st
2nd
3rd
4th
MeanCov+1SD
DV1B
DV1C
DV1E
DV1N
DV1S
DV2B
DV2C
DV2E
DV2N
DV2S
DV3B
DV3C
DV3E
DV3N
DV3S
DV4B
DV4C
DV4E
DV4N
DV4S
DV5B
DV5C
DV5E
DV5N
DV5S
DV6B
DV6C
DV6E
DV6N
DV6S
DV7B
DV7C
DV7E
DV7N
DV7S
DV8B
DV8C
DV8E
DV8N
DV8S
DV9B
DV9C
DV9E
DV9N
DV9S
DV10B
DV10C
DV10E
DV10N
DV10S
Sample

13.

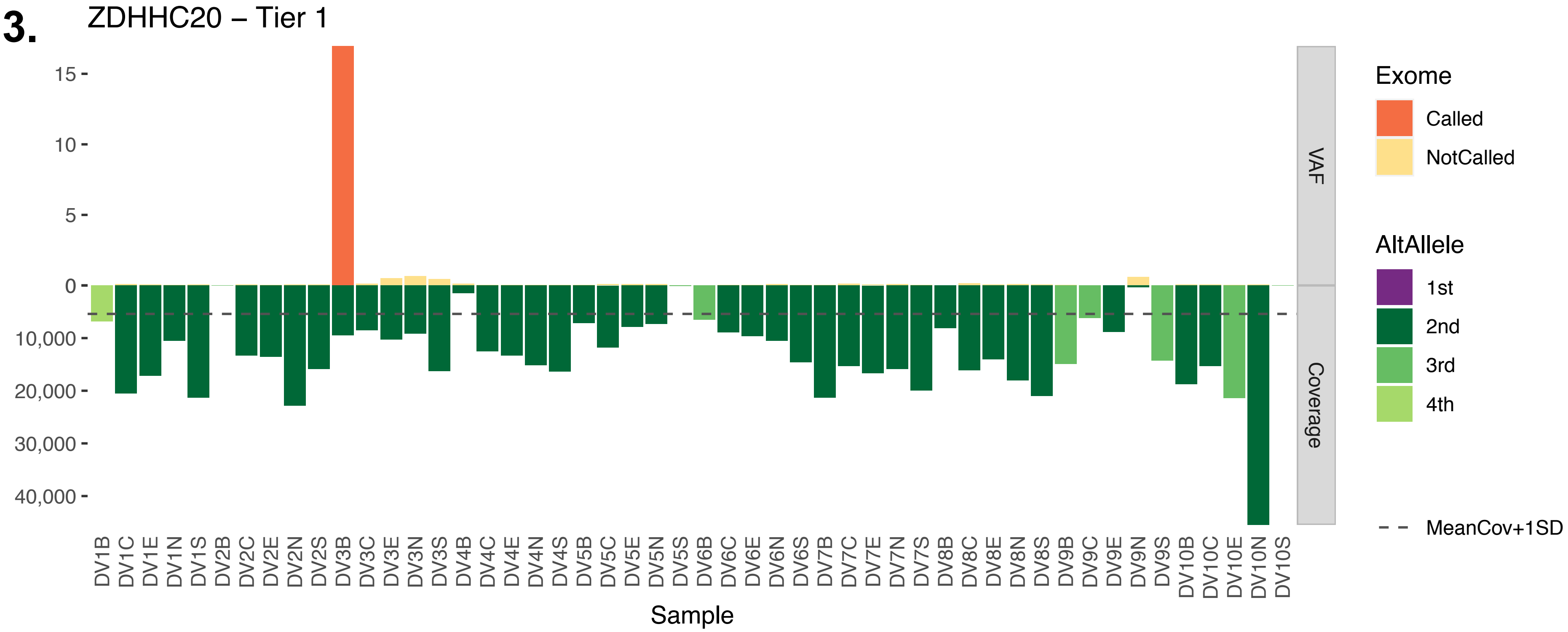
ZDHHC20 – Tier 1
15
10
5
0
10,000
20,000
30,000
40,000
VAF
Coverage
DV1B DV1C DV1E DV1N DV1S DV2B DV2C DV2E DV2N DV2S DV3B DV3C DV3E DV3N DV3S DV4B DV4C DV4E DV4N DV4S DV5B DV5C DV5E DV5N DV5S DV6B DV6C DV6E DV6N DV6S DV7B DV7C DV7E DV7N DV7S DV8B DV8C DV8E DV8N DV8S DV9B DV9C DV9E DV9N DV9S DV10B DV10C DV10E DV10N DV10S
Sample
Exome
Called
NotCalled
AltAllele
1st
2nd
3rd
4th
MeanCov+1SD

14.

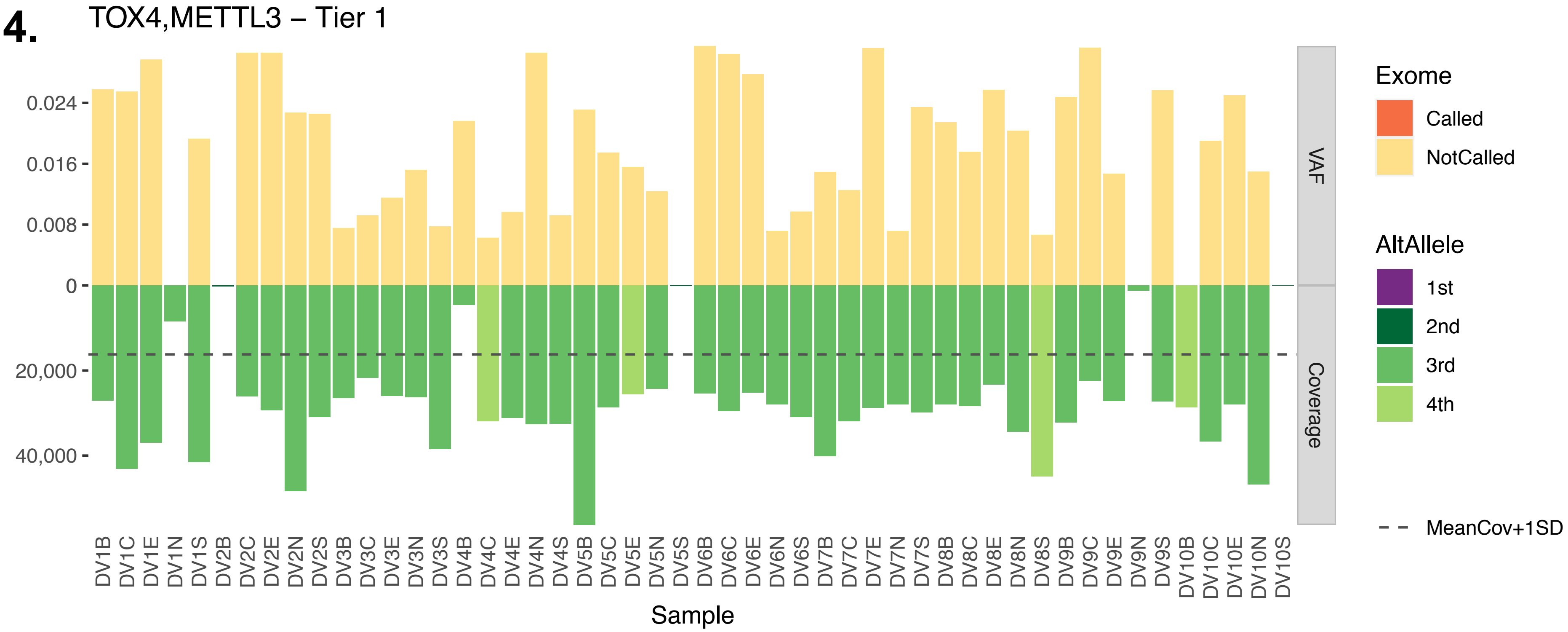

TOX4,METTL3 – Tier 1
0.024
0.016
0.008
0
20,000
40,000
VAF
Coverage
Exome
Called
NotCalled
AltAllele
1st
2nd
3rd
4th
MeanCov+1SD
DV1B DV1C DV1E DV1N DV1S DV2B DV2C DV2E DV2N DV2S DV3B DV3C DV3E DV3N DV3S DV4B DV4C DV4E DV4N DV4S DV5B DV5C DV5E DV5N DV5S DV6B DV6C DV6E DV6N DV6S DV7B DV7C DV7E DV7N DV7S DV8B DV8C DV8E DV8N DV8S DV9B DV9C DV9E DV9N DV9S DV10B DV10C DV10E DV10N DV10S
Sample

15.

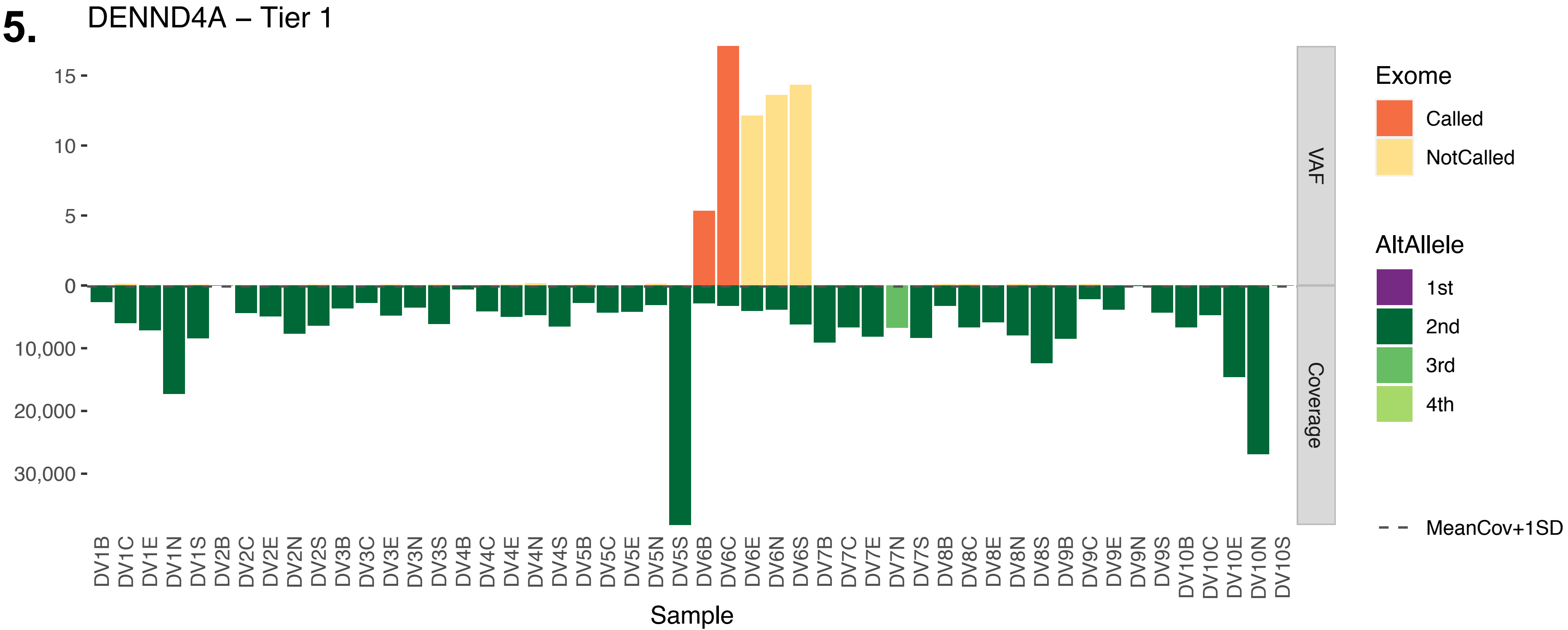

DENND4A – Tier 1
15
10
5
0
10,000
20,000
30,000
VAF
Coverage
Exome
Called
NotCalled
AltAllele
1st
2nd
3rd
4th
MeanCov+1SD
DV1B
DV1C
DV1E
DV1N
DV1S
DV2B
DV2C
DV2E
DV2N
DV2S
DV3B
DV3C
DV3E
DV3N
DV3S
DV4B
DV4C
DV4E
DV4N
DV4S
DV5B
DV5C
DV5E
DV5N
DV5S
DV6B
DV6C
DV6E
DV6N
DV6S
DV7B
DV7C
DV7E
DV7N
DV7S
DV8B
DV8C
DV8E
DV8N
DV8S
DV9B
DV9C
DV9E
DV9N
DV9S
DV10B
DV10C
DV10E
DV10N
DV10S
Sample

16.

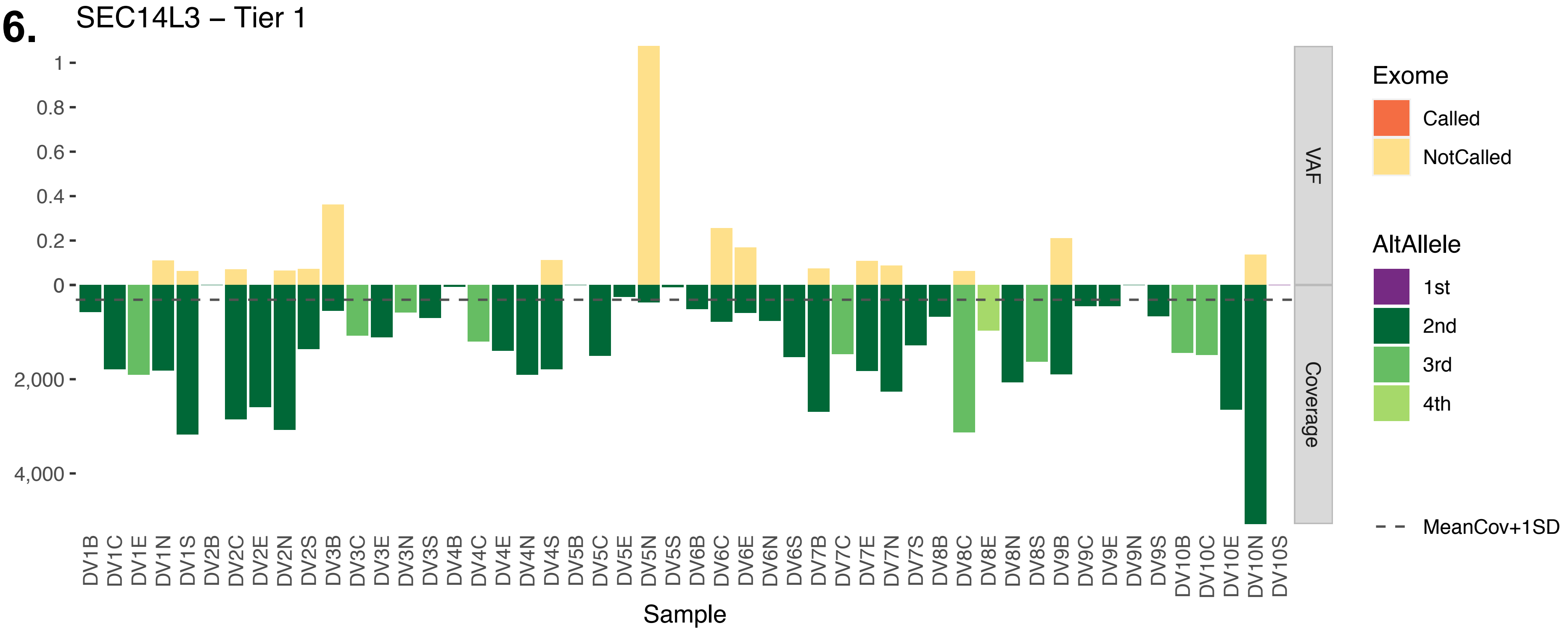
SEC14L3 – Tier 1
1
0.8
0.6
0.4
0.2
0
2,000
4,000
VAF
Coverage
Exome
Called
NotCalled
AltAllele
1st
2nd
3rd
4th
MeanCov+1SD
DV1B
DV1C
DV1E
DV1N
DV1S
DV2B
DV2C
DV2E
DV2N
DV2S
DV3B
DV3C
DV3E
DV3N
DV3S
DV4B
DV4C
DV4E
DV4N
DV4S
DV5B
DV5C
DV5E
DV5N
DV5S
DV6B
DV6C
DV6E
DV6N
DV6S
DV7B
DV7C
DV7E
DV7N
DV7S
DV8B
DV8C
DV8E
DV8N
DV8S
DV9B
DV9C
DV9E
DV9N
DV9S
DV10B
DV10C
DV10E
DV10N
DV10S
Sample

17. FOXRED2 – Tier 1

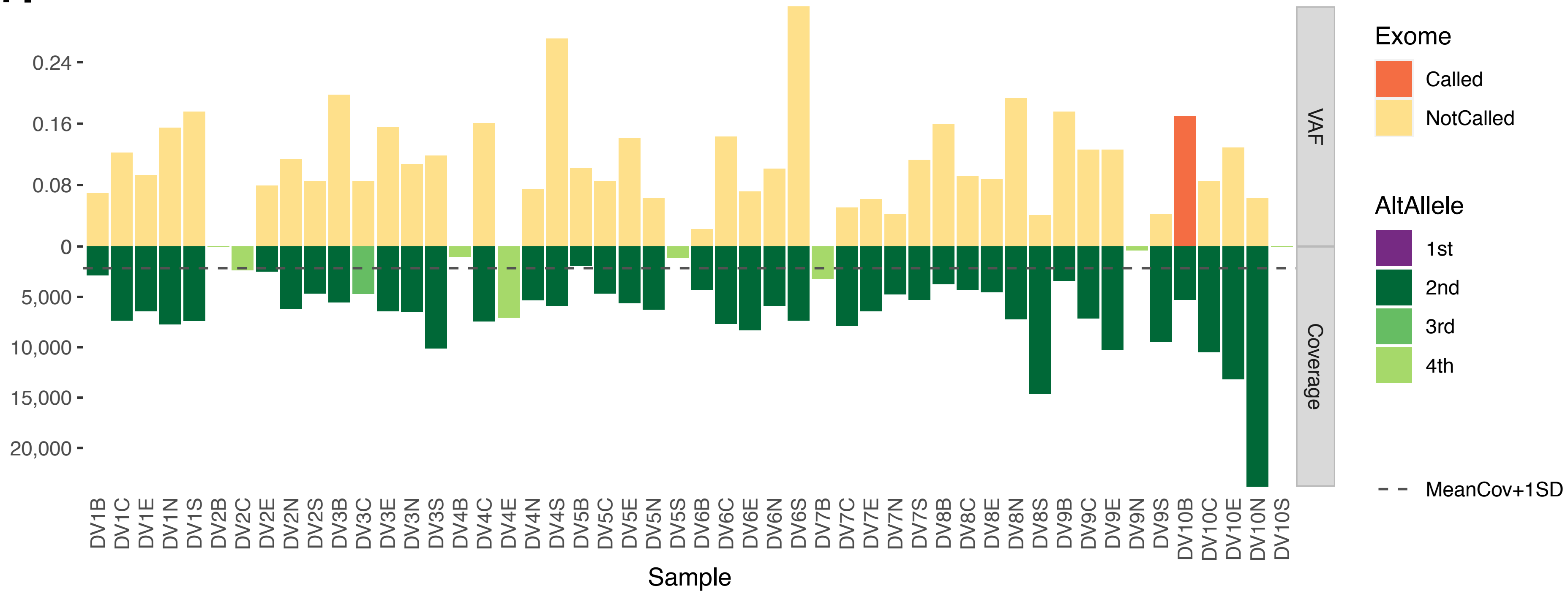

18.

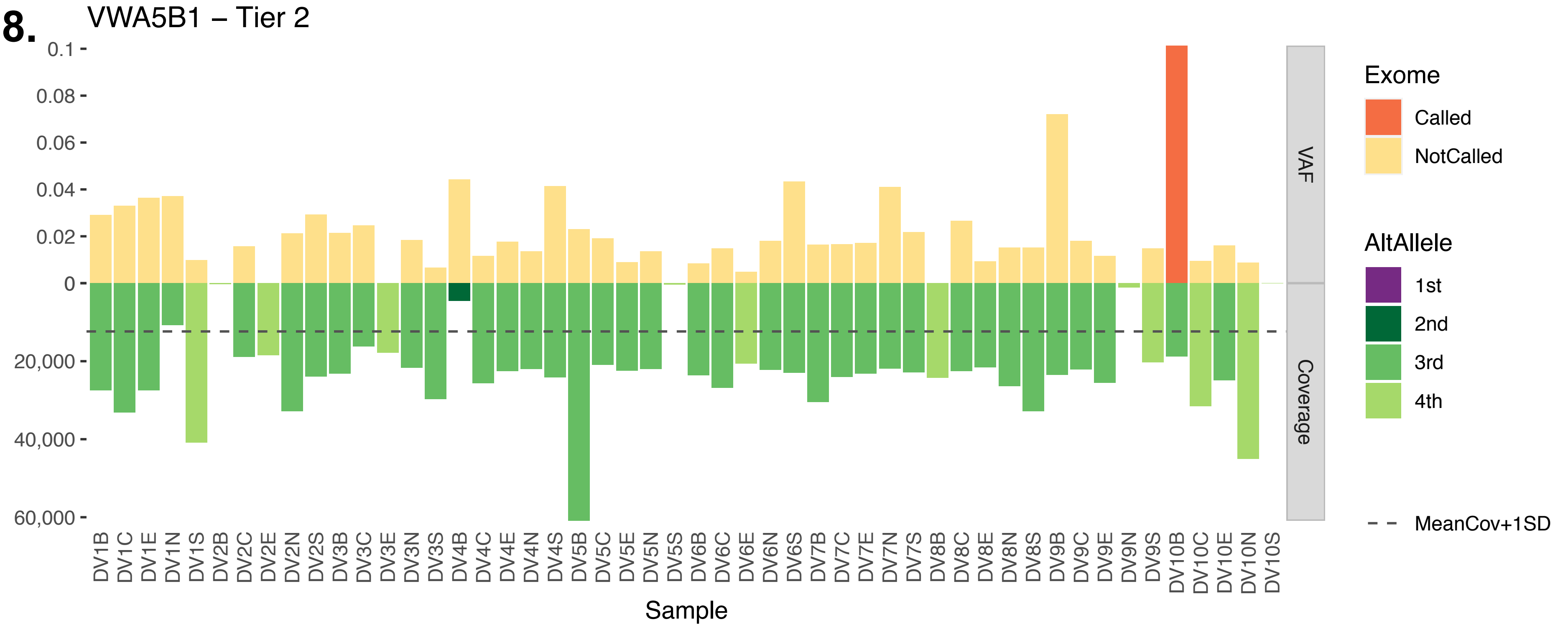

VWA5B1 – Tier 2
0.1
0.08
0.06
0.04
0.02
0
20,000
40,000
60,000
VAF
Coverage
DV1B DV1C DV1E DV1N DV1S DV2B DV2C DV2E DV2N DV2S DV3B DV3C DV3E DV3N DV3S DV4B DV4C DV4E DV4N DV4S DV5B DV5C DV5E DV5N DV5S DV6B DV6C DV6E DV6N DV6S DV7B DV7C DV7E DV7N DV7S DV8B DV8C DV8E DV8N DV8S DV9B DV9C DV9E DV9N DV9S DV10B DV10C DV10E DV10N DV10S
Sample
Exome
Called
NotCalled
AltAllele
1st
2nd
3rd
4th
MeanCov+1SD

19.

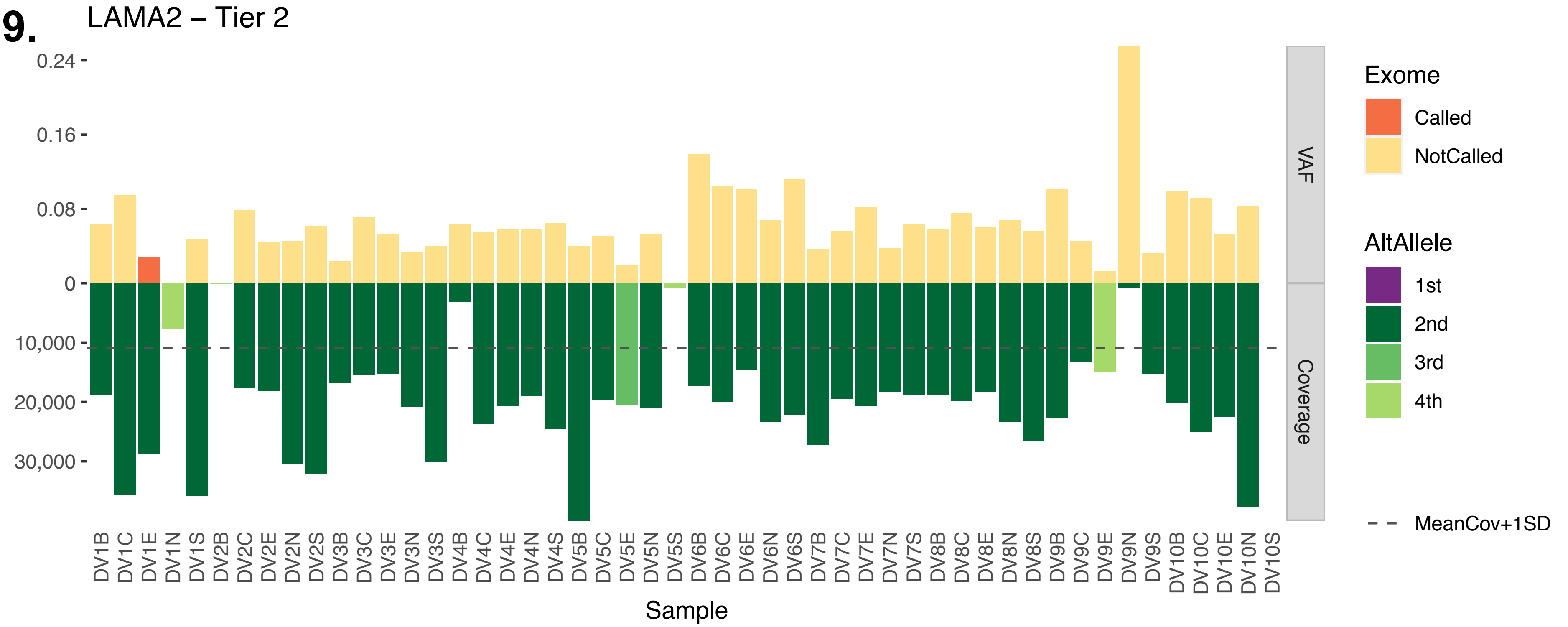
LAMA2 – Tier 2
0.24
0.16
0.08
0
10,000
20,000
30,000
VAF
Coverage
Sample
DV1B DV1C DV1E DV1N DV1S DV2B DV2C DV2E DV2N DV2S DV3B DV3C DV3E DV3N DV3S DV4B DV4C DV4E DV4N DV4S DV5B DV5C DV5E DV5N DV5S DV6B DV6C DV6E DV6N DV6S DV7B DV7C DV7E DV7N DV7S DV8B DV8C DV8E DV8N DV8S DV9B DV9C DV9E DV9N DV9S DV10B DV10C DV10E DV10N DV10S
Exome
Called
NotCalled
AltAllele
1st
2nd
3rd
4th
MeanCov+1SD

20.

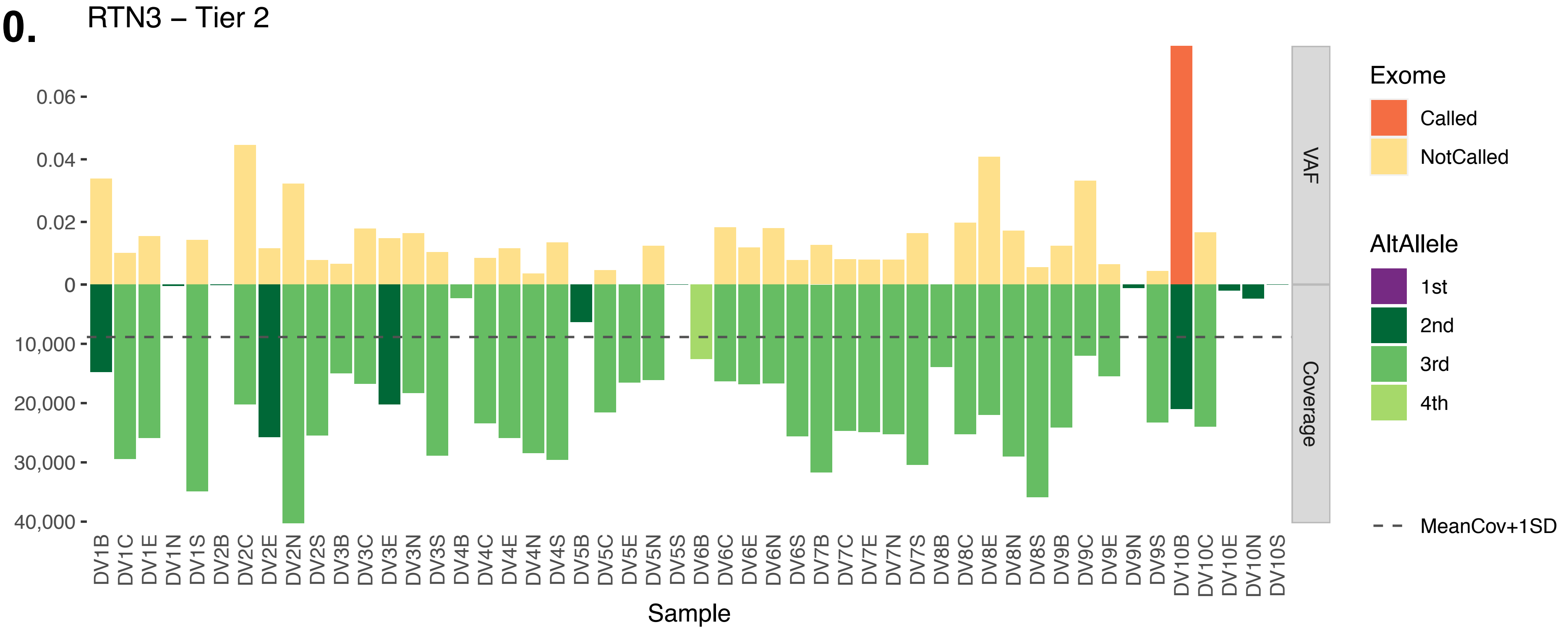

RTN3 – Tier 2
0.06
0.04
0.02
0
10,000
20,000
30,000
40,000
VAF
Coverage
DV1B DV1C DV1E DV1N DV1S DV2B DV2C DV2E DV2N DV2S DV3B DV3C DV3E DV3N DV3S DV4B DV4C DV4E DV4N DV4S DV5B DV5C DV5E DV5N DV5S DV6B DV6C DV6E DV6N DV6S DV7B DV7C DV7E DV7N DV7S DV8B DV8C DV8E DV8N DV8S DV9B DV9C DV9E DV9N DV9S DV10B DV10C DV10E DV10N DV10S
Sample
Exome
Called
NotCalled
AltAllele
1st
2nd
3rd
4th
MeanCov+1SD

21.

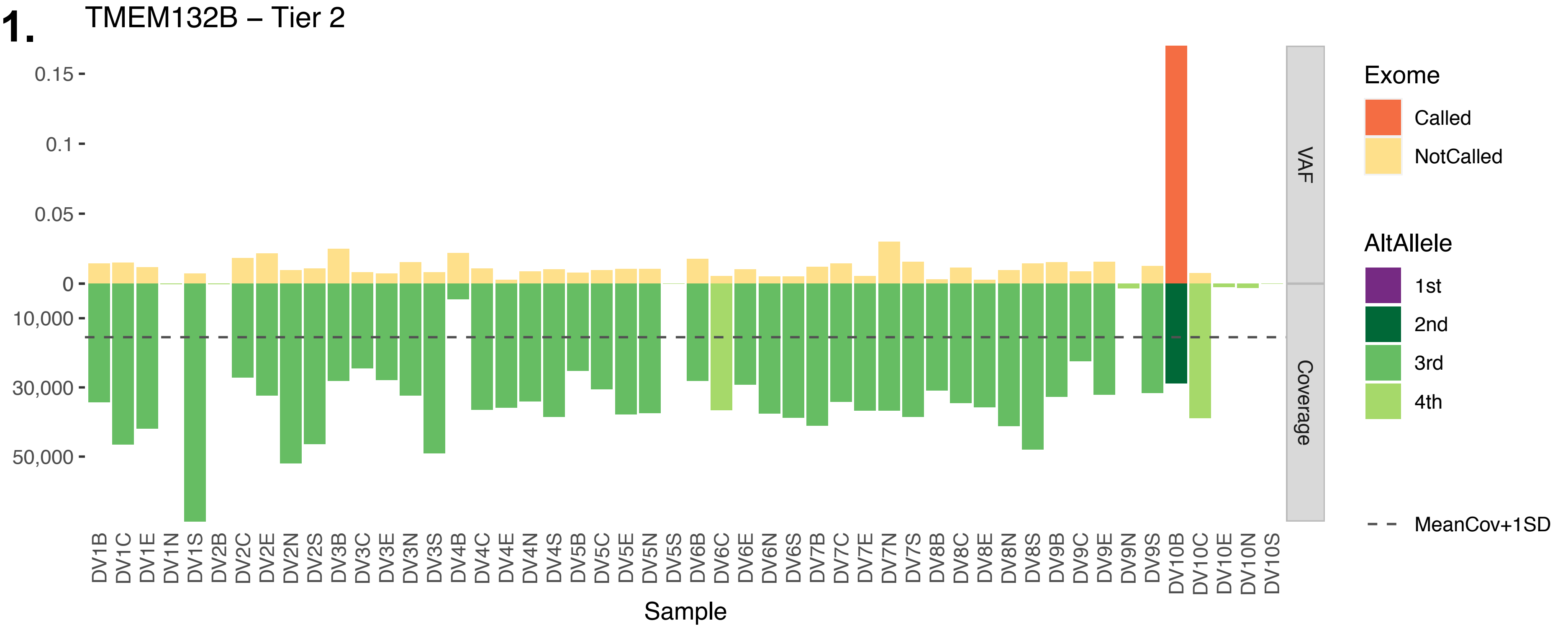
TMEM132B – Tier 2
0.15
0.1
0.05
0
10,000
30,000
50,000
VAF
Coverage
DV1B DV1C DV1E DV1N DV1S DV2B DV2C DV2E DV2N DV2S DV3B DV3C DV3E DV3N DV3S DV4B DV4C DV4E DV4N DV4S DV5B DV5C DV5E DV5N DV5S DV6B DV6C DV6E DV6N DV6S DV7B DV7C DV7E DV7N DV7S DV8B DV8C DV8E DV8N DV8S DV9B DV9C DV9E DV9N DV9S DV10B DV10C DV10E DV10N DV10S
Sample
Exome
Called
NotCalled
AltAllele
1st
2nd
3rd
4th
MeanCov+1SD

22.

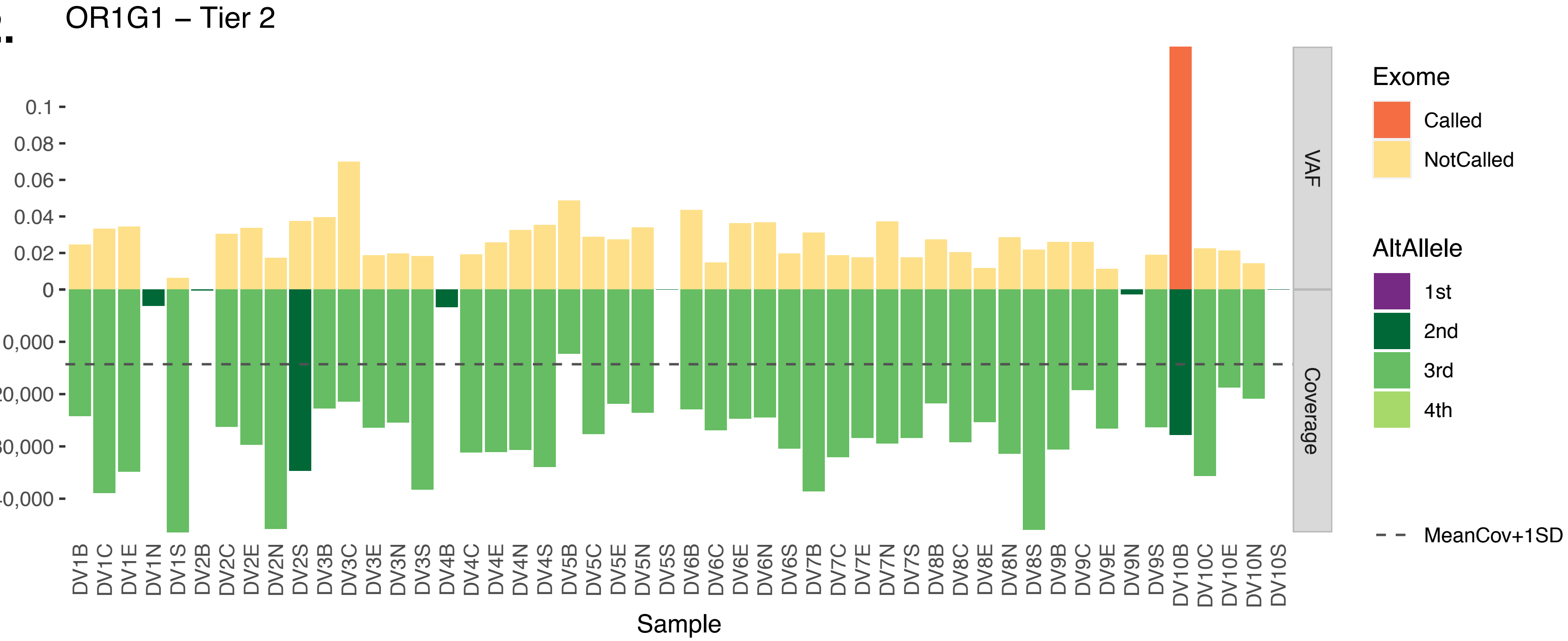
OR1G1 – Tier 2
0.1
0.08
0.06
0.04
0.02
0
10,000
20,000
30,000
40,000
VAF
Coverage
Exome
Called
NotCalled
AltAllele
1st
2nd
3rd
4th
MeanCov+1SD
DV1B DV1C DV1E DV1N DV1S DV2B DV2C DV2E DV2N DV2S DV3B DV3C DV3E DV3N DV3S DV4B DV4C DV4E DV4N DV4S DV5B DV5C DV5E DV5N DV5S DV6B DV6C DV6E DV6N DV6S DV7B DV7C DV7E DV7N DV7S DV8B DV8C DV8E DV8N DV8S DV9B DV9C DV9E DV9N DV9S DV10B DV10C DV10E DV10N DV10S
Sample

23.

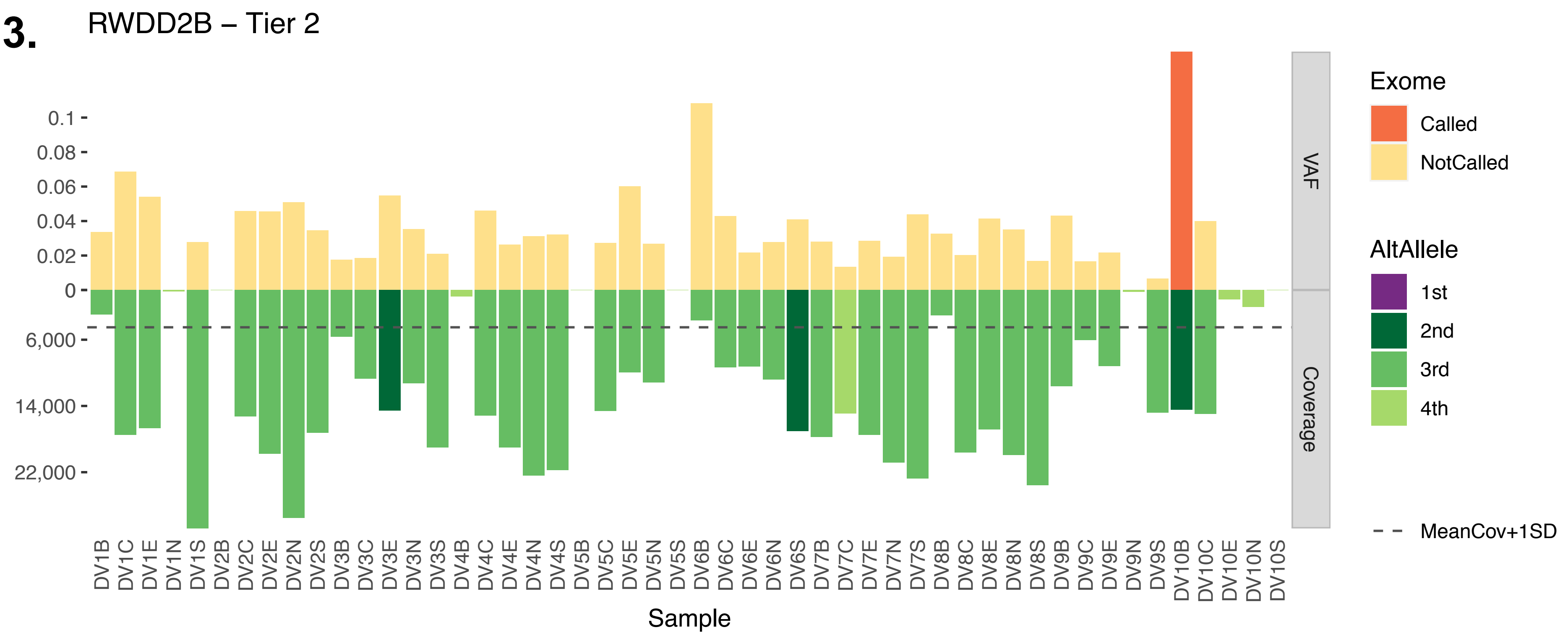
RWDD2B – Tier 2
0.1
0.08
0.06
0.04
0.02
0
6,000
14,000
22,000
VAF
Coverage
Exome
Called
NotCalled
AltAllele
1st
2nd
3rd
4th
MeanCov+1SD
DV1B
DV1C
DV1E
DV1N
DV1S
DV2B
DV2C
DV2E
DV2N
DV2S
DV3B
DV3C
DV3E
DV3N
DV3S
DV4B
DV4C
DV4E
DV4N
DV4S
DV5B
DV5C
DV5E
DV5N
DV5S
DV6B
DV6C
DV6E
DV6N
DV6S
DV7B
DV7C
DV7E
DV7N
DV7S
DV8B
DV8C
DV8E
DV8N
DV8S
DV9B
DV9C
DV9E
DV9N
DV9S
DV10B
DV10C
DV10E
DV10N
DV10S
Sample

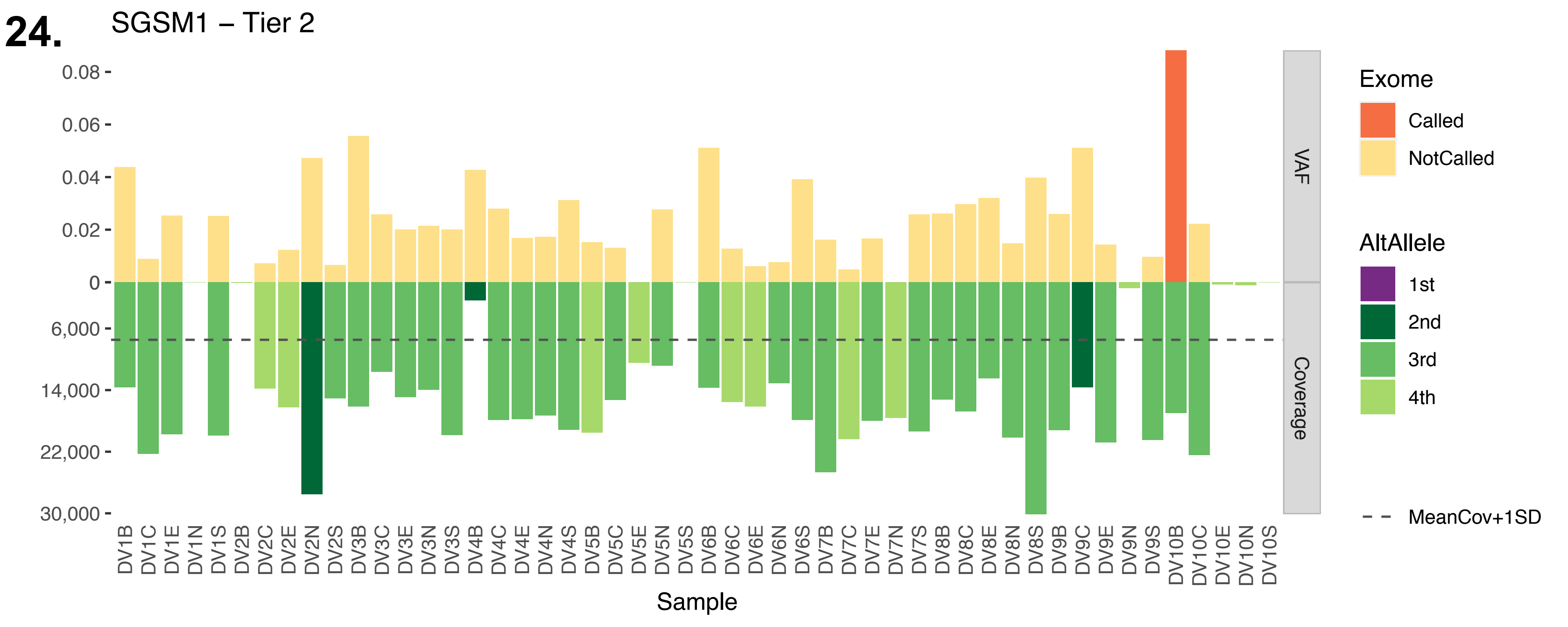

24.
SGSM1 – Tier 2
0.08
0.06
0.04
0.02
0
6,000
14,000
22,000
30,000
VAF
Coverage
Exome
Called
NotCalled
AltAllele
1st
2nd
3rd
4th
MeanCov+1SD
DV1B
DV1C
DV1E
DV1N
DV1S
DV2B
DV2C
DV2E
DV2N
DV2S
DV3B
DV3C
DV3E
DV3N
DV3S
DV4B
DV4C
DV4E
DV4N
DV4S
DV5B
DV5C
DV5E
DV5N
DV5S
DV6B
DV6C
DV6E
DV6N
DV6S
DV7B
DV7C
DV7E
DV7N
DV7S
DV8B
DV8C
DV8E
DV8N
DV8S
DV9B
DV9C
DV9E
DV9N
DV9S
DV10B
DV10C
DV10E
DV10N
DV10S
Sample

25. PQLC2 – Tier 3

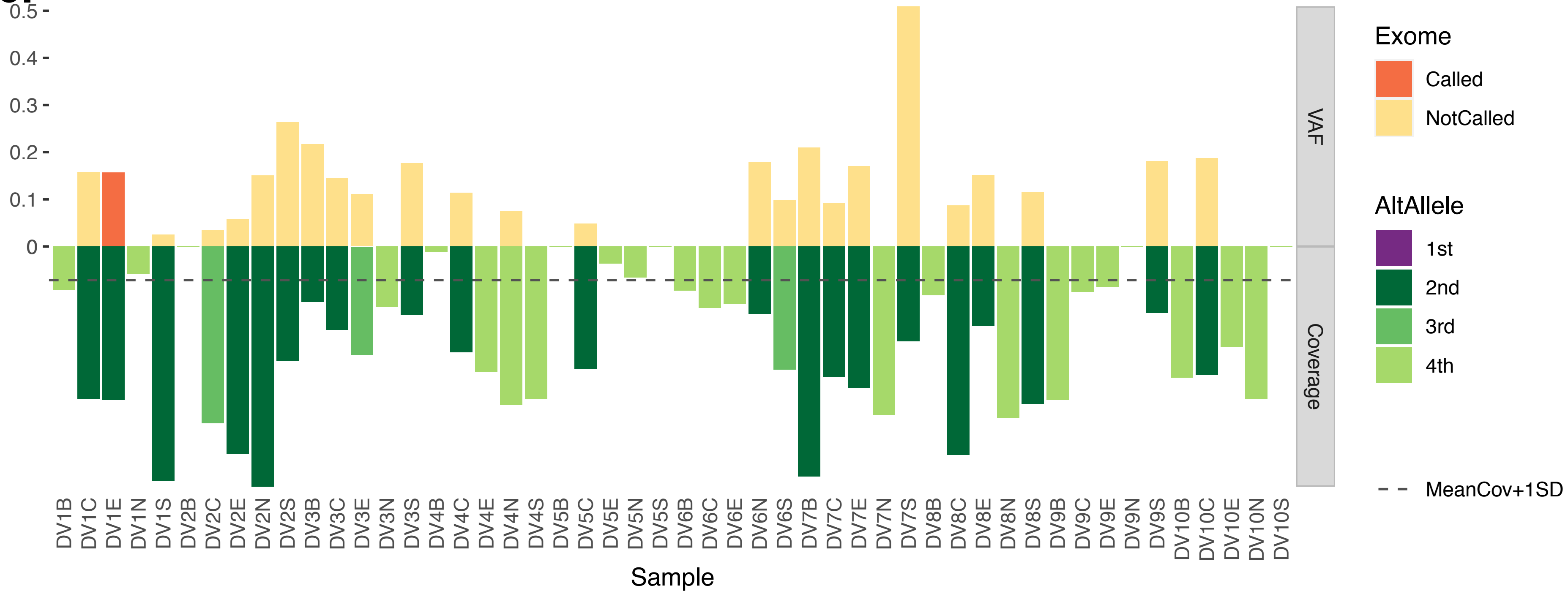

26.

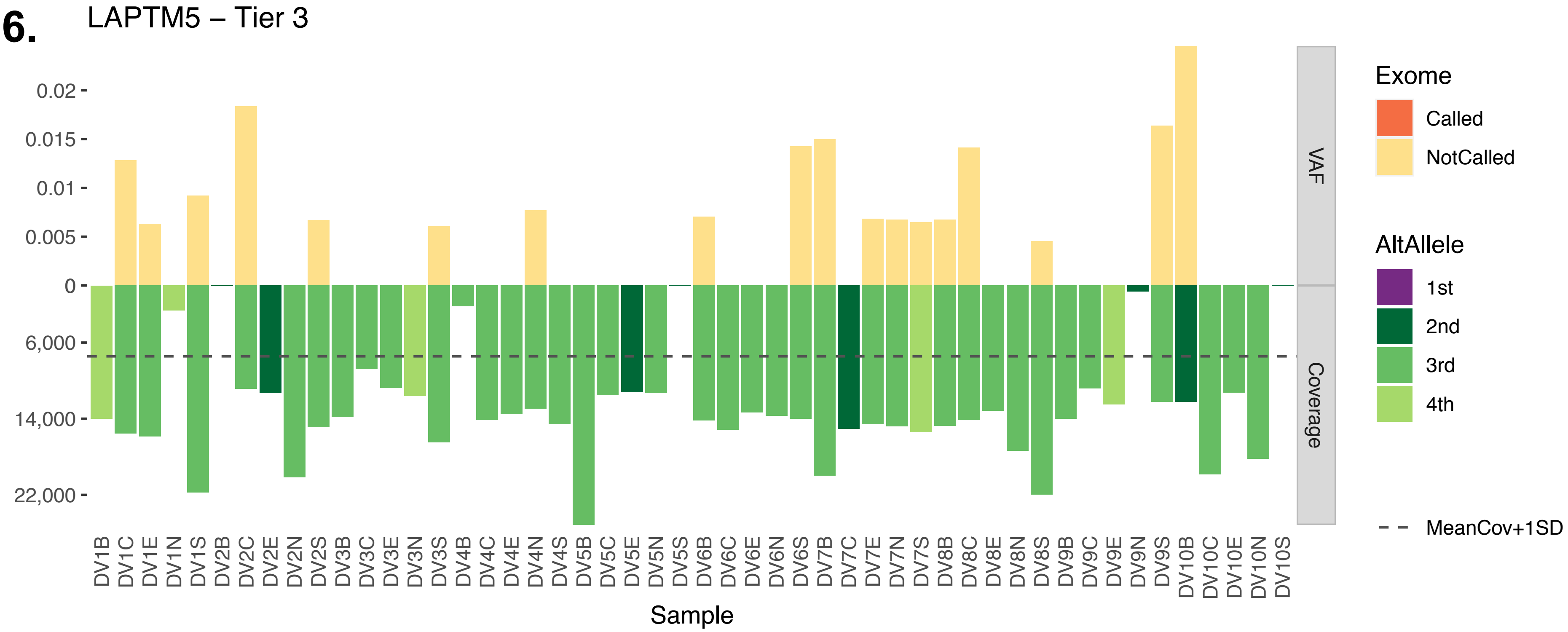

LAPTM5 – Tier 3
0.02
0.015
0.01
0.005
0
6,000
14,000
22,000
VAF
Coverage
DV1B DV1C DV1E DV1N DV1S DV2B DV2C DV2E DV2N DV2S DV3B DV3C DV3E DV3N DV3S DV4B DV4C DV4E DV4N DV4S DV5B DV5C DV5E DV5N DV5S DV6B DV6C DV6E DV6N DV6S DV7B DV7C DV7E DV7N DV7S DV8B DV8C DV8E DV8N DV8S DV9B DV9C DV9E DV9N DV9S DV10B DV10C DV10E DV10N DV10S
Sample
Exome
Called
NotCalled
AltAllele
1st
2nd
3rd
4th
MeanCov+1SD

27.

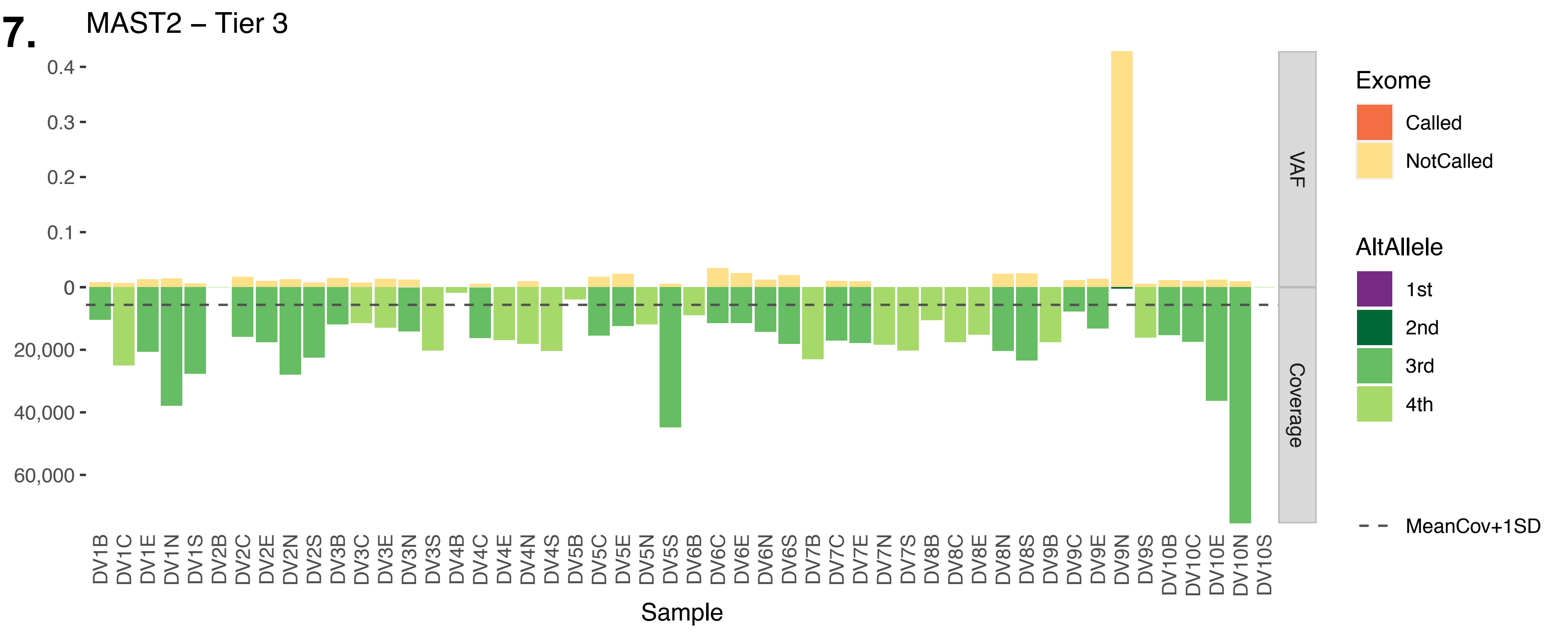
MAST2 – Tier 3
0.4
0.3
0.2
0.1
0
20,000
40,000
60,000
VAF
Coverage
Sample
DV1B DV1C DV1E DV1N DV1S DV2B DV2C DV2E DV2N DV2S DV3B DV3C DV3E DV3N DV3S DV4B DV4C DV4E DV4N DV4S DV5B DV5C DV5E DV5N DV5S DV6B DV6C DV6E DV6N DV6S DV7B DV7C DV7E DV7N DV7S DV8B DV8C DV8E DV8N DV8S DV9B DV9C DV9E DV9N DV9S DV10B DV10C DV10E DV10N DV10S
Exome
Called
NotCalled
AltAllele
1st
2nd
3rd
4th
MeanCov+1SD

28.

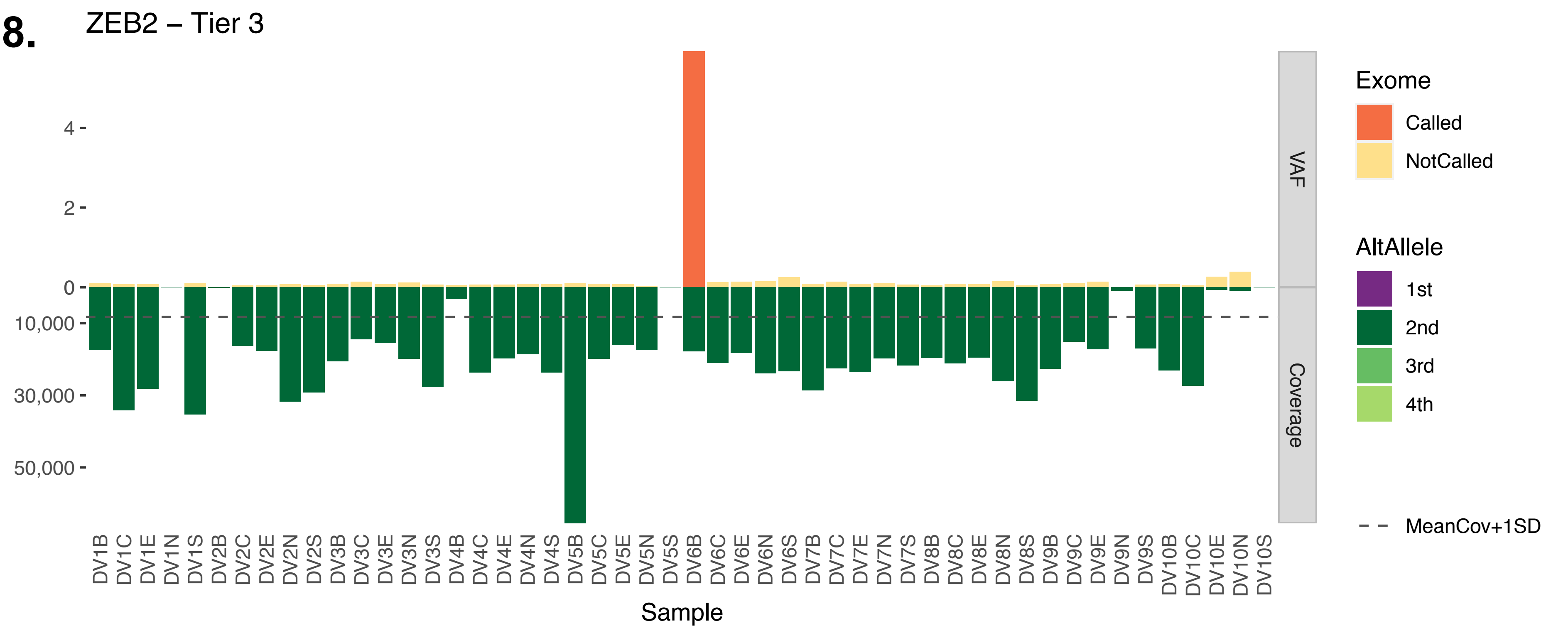

ZEB2 – Tier 3
4
2
0
10,000
30,000
50,000
VAF
Coverage
Exome
Called
NotCalled
AltAllele
1st
2nd
3rd
4th
MeanCov+1SD
DV1B DV1C DV1E DV1N DV1S DV2B DV2C DV2E DV2N DV2S DV3B DV3C DV3E DV3N DV3S DV4B DV4C DV4E DV4N DV4S DV5B DV5C DV5E DV5N DV5S DV6B DV6C DV6E DV6N DV6S DV7B DV7C DV7E DV7N DV7S DV8B DV8C DV8E DV8N DV8S DV9B DV9C DV9E DV9N DV9S DV10B DV10C DV10E DV10N DV10S
Sample

29.

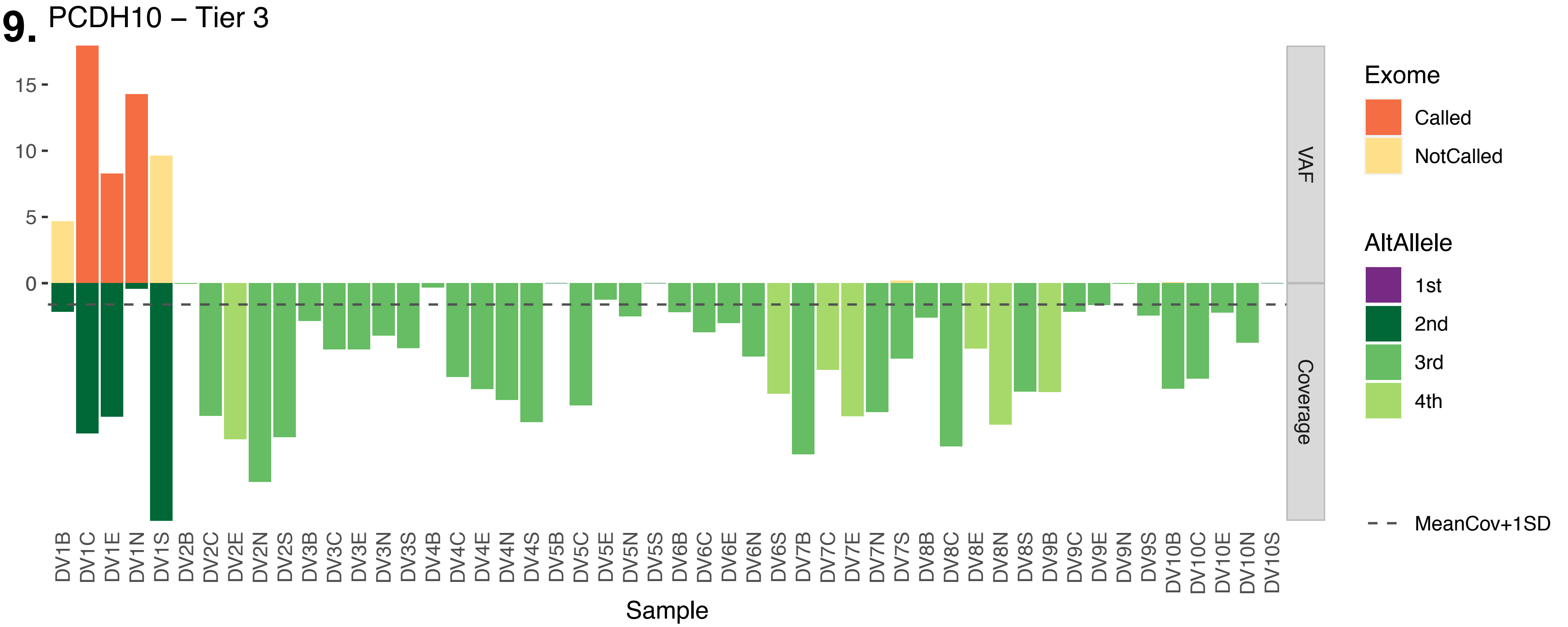
PCDH10 – Tier 3
15
10
5
0
VAF
Coverage
Exome
Called
NotCalled
AltAllele
1st
2nd
3rd
4th
MeanCov+1SD
DV1B DV1C DV1E DV1N DV1S DV2B DV2C DV2E DV2N DV2S DV3B DV3C DV3E DV3N DV3S DV4B DV4C DV4E DV4N DV4S DV5B DV5C DV5E DV5N DV5S DV6B DV6C DV6E DV6N DV6S DV7B DV7C DV7E DV7N DV7S DV8B DV8C DV8E DV8N DV8S DV9B DV9C DV9E DV9N DV9S DV10B DV10C DV10E DV10N DV10S
Sample

30.

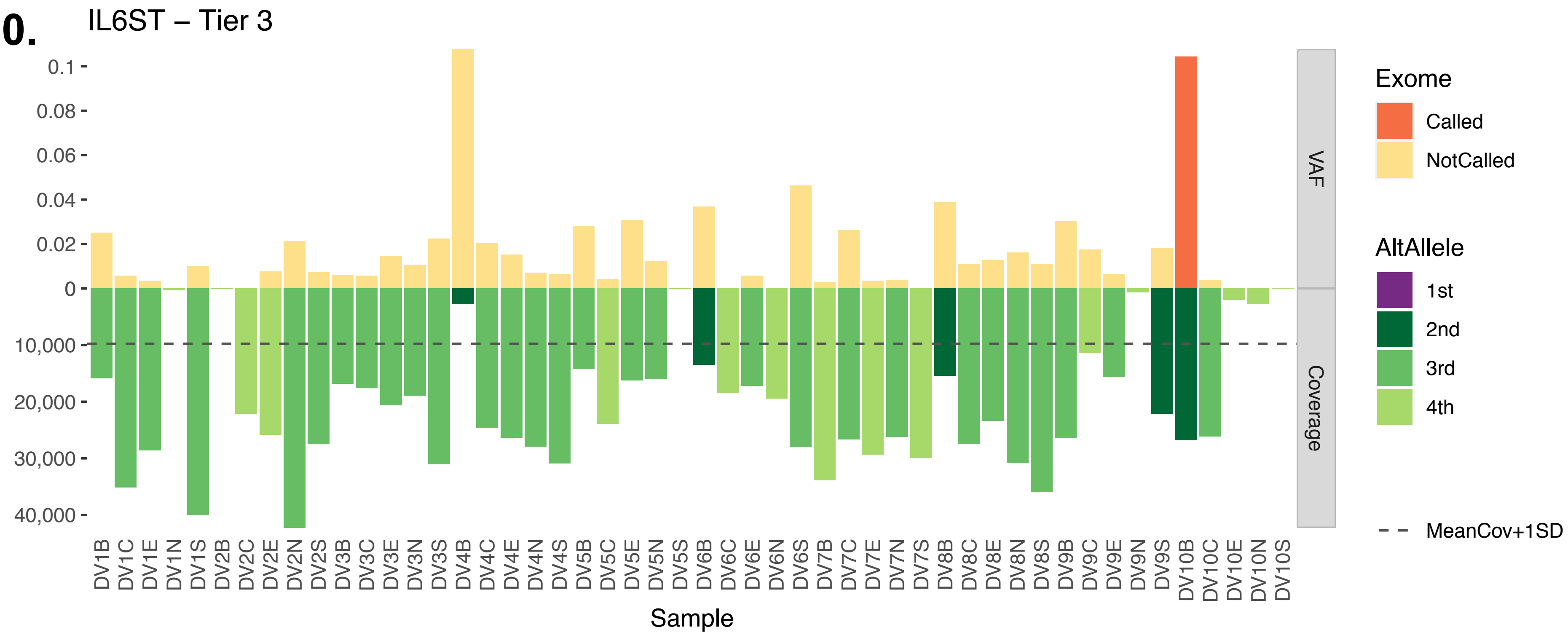
IL6ST – Tier 3
0.1
0.08
0.06
0.04
0.02
0
10,000
20,000
30,000
40,000
VAF
Coverage
Exome
Called
NotCalled
AltAllele
1st
2nd
3rd
4th
MeanCov+1SD
DV1B
DV1C
DV1E
DV1N
DV1S
DV2B
DV2C
DV2E
DV2N
DV2S
DV3B
DV3C
DV3E
DV3N
DV3S
DV4B
DV4C
DV4E
DV4N
DV4S
DV5B
DV5C
DV5E
DV5N
DV5S
DV6B
DV6C
DV6E
DV6N
DV6S
DV7B
DV7C
DV7E
DV7N
DV7S
DV8B
DV8C
DV8E
DV8N
DV8S
DV9B
DV9C
DV9E
DV9N
DV9S
DV10B
DV10C
DV10E
DV10N
DV10S
Sample

31.

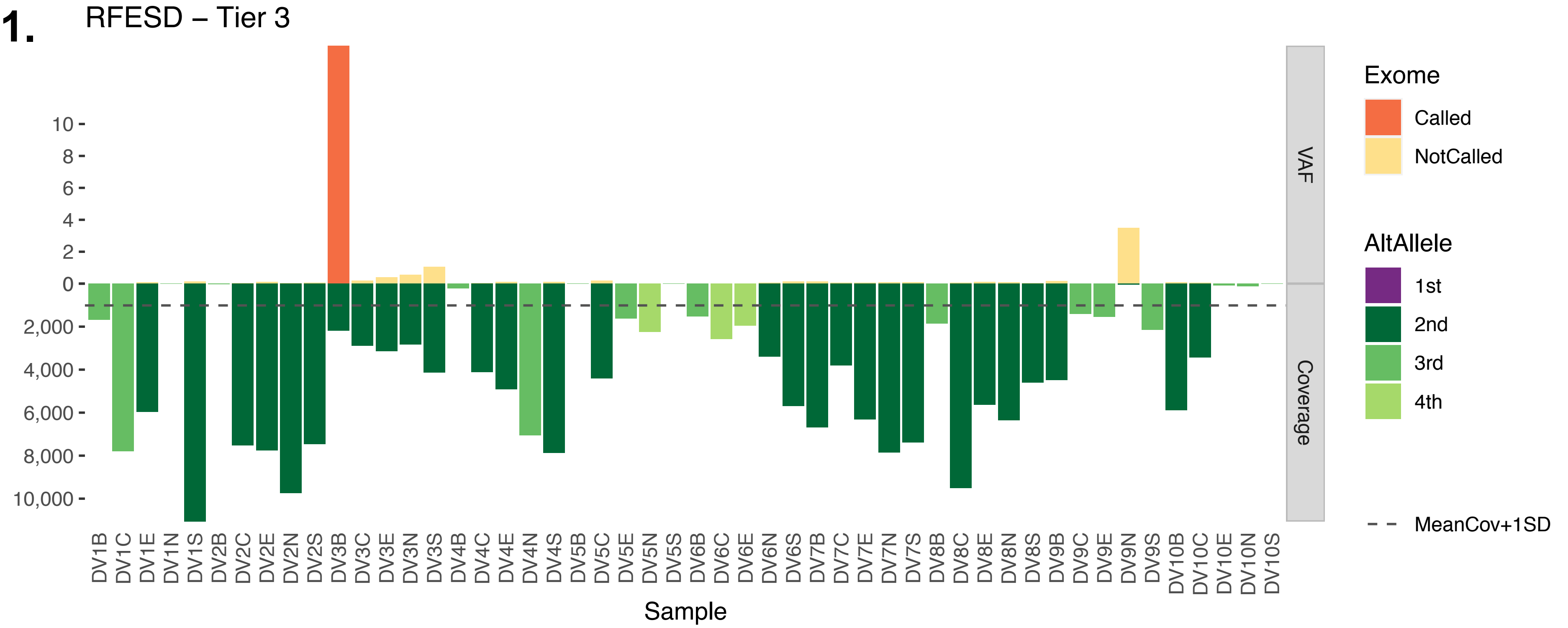

RFESD – Tier 3
10
8
6
4
2
0
2,000
4,000
6,000
8,000
10,000
VAF
Coverage
DV1B DV1C DV1E DV1N DV1S DV2B DV2C DV2E DV2N DV2S DV3B DV3C DV3E DV3N DV3S DV4B DV4C DV4E DV4N DV4S DV5B DV5C DV5E DV5N DV5S DV6B DV6C DV6E DV6N DV6S DV7B DV7C DV7E DV7N DV7S DV8B DV8C DV8E DV8N DV8S DV9B DV9C DV9E DV9N DV9S DV10B DV10C DV10E DV10N DV10S
Sample
Exome
Called
NotCalled
AltAllele
1st
2nd
3rd
4th
MeanCov+1SD

32.

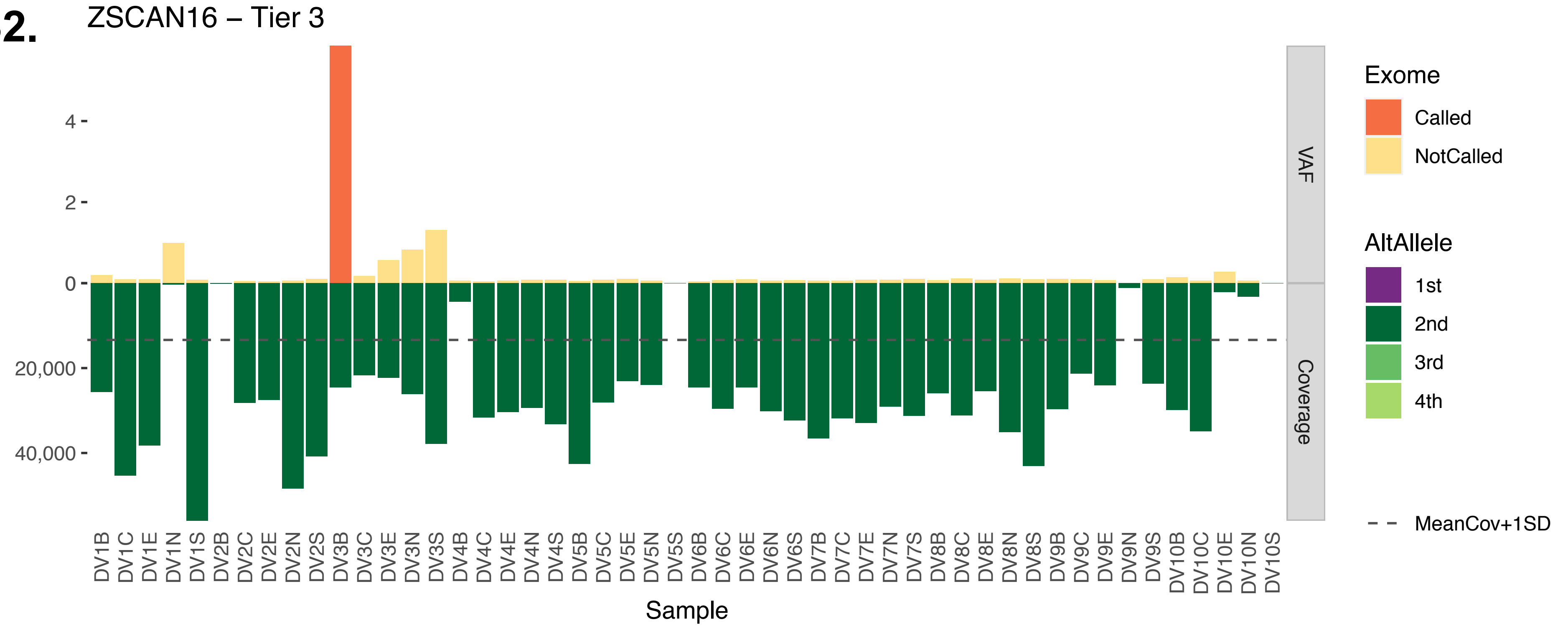

ZSCAN16 – Tier 3
4
2
0
20,000
40,000
VAF
Coverage
Exome
Called
NotCalled
AltAllele
1st
2nd
3rd
4th
MeanCov+1SD
DV1B
DV1C
DV1E
DV1N
DV1S
DV2B
DV2C
DV2E
DV2N
DV2S
DV3B
DV3C
DV3E
DV3N
DV3S
DV4B
DV4C
DV4E
DV4N
DV4S
DV5B
DV5C
DV5E
DV5N
DV5S
DV6B
DV6C
DV6E
DV6N
DV6S
DV7B
DV7C
DV7E
DV7N
DV7S
DV8B
DV8C
DV8E
DV8N
DV8S
DV9B
DV9C
DV9E
DV9N
DV9S
DV10B
DV10C
DV10E
DV10N
DV10S
Sample

33.

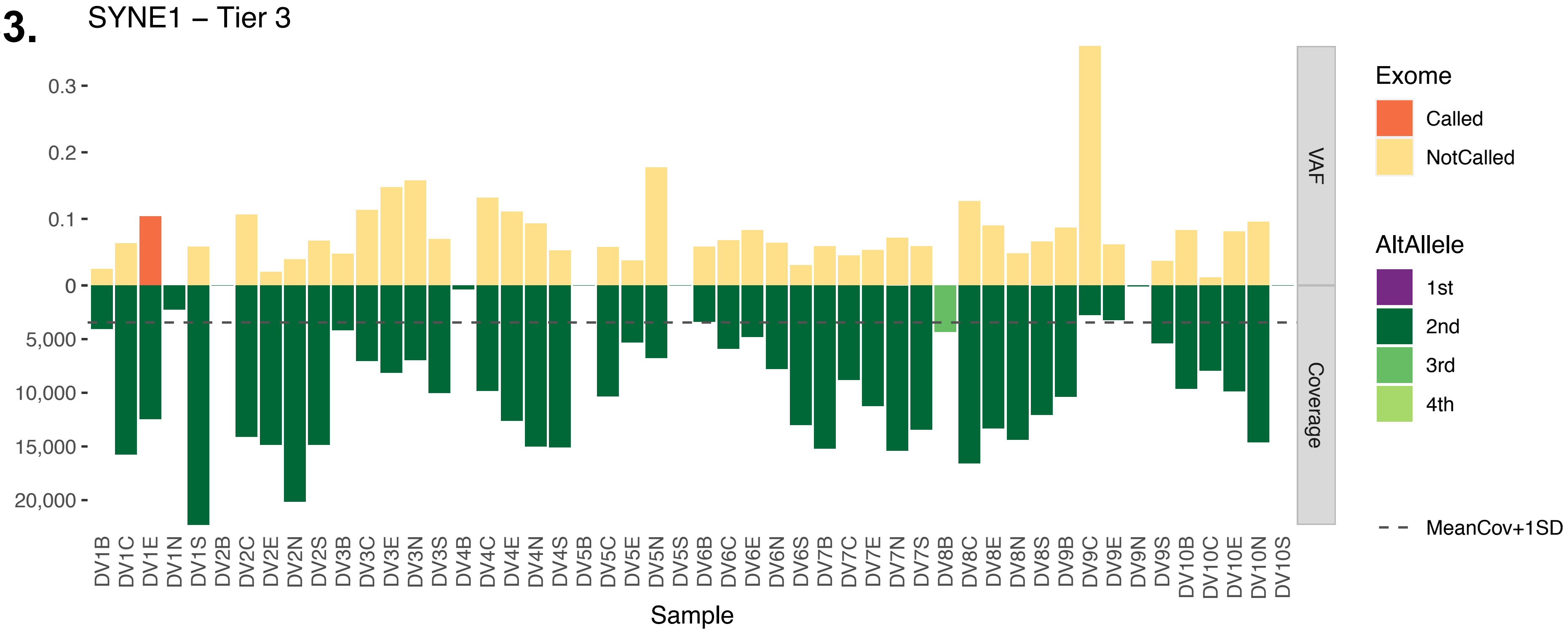

SYNE1 – Tier 3
0.3
0.2
0.1
0
5,000
10,000
15,000
20,000
VAF
Coverage
DV1B
DV1C
DV1E
DV1N
DV1S
DV2B
DV2C
DV2E
DV2N
DV2S
DV3B
DV3C
DV3E
DV3N
DV3S
DV4B
DV4C
DV4E
DV4N
DV4S
DV5B
DV5C
DV5E
DV5N
DV5S
DV6B
DV6C
DV6E
DV6N
DV6S
DV7B
DV7C
DV7E
DV7N
DV7S
DV8B
DV8C
DV8E
DV8N
DV8S
DV9B
DV9C
DV9E
DV9N
DV9S
DV10B
DV10C
DV10E
DV10N
DV10S
Sample
Exome
Called
NotCalled
AltAllele
1st
2nd
3rd
4th
MeanCov+1SD

34.

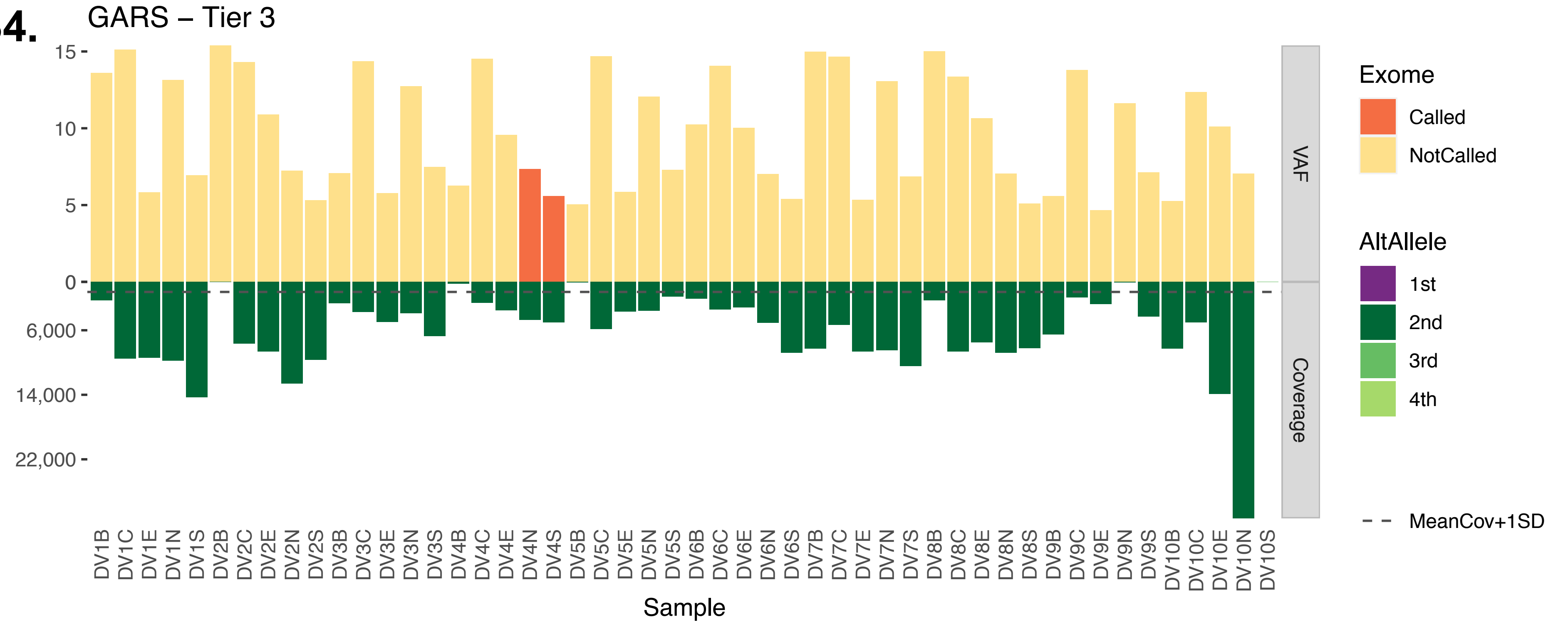

GARS – Tier 3
15
10
5
0
6,000
14,000
22,000
VAF
Coverage
DV1B
DV1C
DV1E
DV1N
DV1S
DV2B
DV2C
DV2E
DV2N
DV2S
DV3B
DV3C
DV3E
DV3N
DV3S
DV4B
DV4C
DV4E
DV4N
DV4S
DV5B
DV5C
DV5E
DV5N
DV5S
DV6B
DV6C
DV6E
DV6N
DV6S
DV7B
DV7C
DV7E
DV7N
DV7S
DV8B
DV8C
DV8E
DV8N
DV8S
DV9B
DV9C
DV9E
DV9N
DV9S
DV10B
DV10C
DV10E
DV10N
DV10S
Sample
Exome
Called
NotCalled
AltAllele
1st
2nd
3rd
4th
MeanCov+1SD

35.

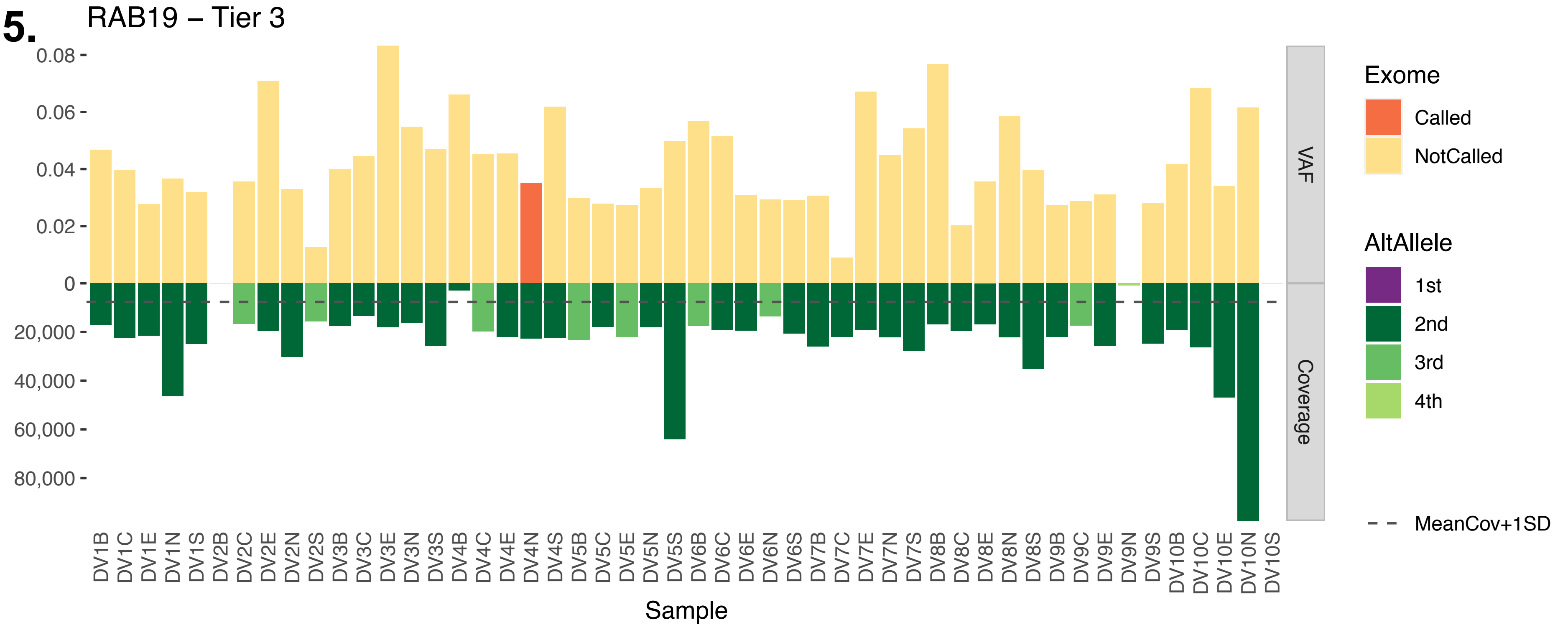

RAB19 – Tier 3
0.08
0.06
0.04
0.02
0
20,000
40,000
60,000
80,000
VAF
Coverage
DV1B DV1C DV1E DV1N DV1S DV2B DV2C DV2E DV2N DV2S DV3B DV3C DV3E DV3N DV3S DV4B DV4C DV4E DV4N DV4S DV5B DV5C DV5E DV5N DV5S DV6B DV6C DV6E DV6N DV6S DV7B DV7C DV7E DV7N DV7S DV8B DV8C DV8E DV8N DV8S DV9B DV9C DV9E DV9N DV9S DV10B DV10C DV10E DV10N DV10S
Sample
Exome
Called
NotCalled
AltAllele
1st
2nd
3rd
4th
MeanCov+1SD

36.

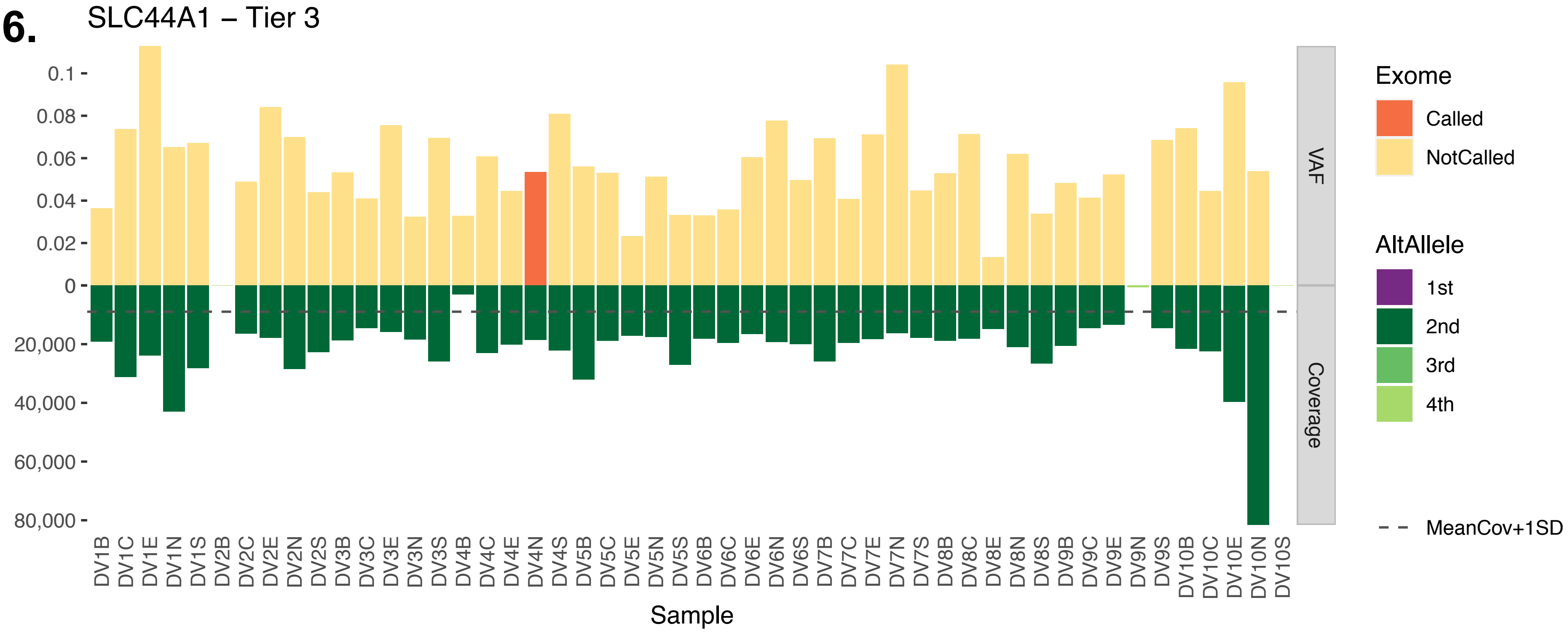

SLC44A1 – Tier 3
0.1
0.08
0.06
0.04
0.02
0
20,000
40,000
60,000
80,000
VAF
Coverage
Exome
Called
NotCalled
AltAllele
1st
2nd
3rd
4th
MeanCov+1SD
DV1B
DV1C
DV1E
DV1N
DV1S
DV2B
DV2C
DV2E
DV2N
DV2S
DV3B
DV3C
DV3E
DV3N
DV3S
DV4B
DV4C
DV4E
DV4N
DV4S
DV5B
DV5C
DV5E
DV5N
DV5S
DV6B
DV6C
DV6E
DV6N
DV6S
DV7B
DV7C
DV7E
DV7N
DV7S
DV8B
DV8C
DV8E
DV8N
DV8S
DV9B
DV9C
DV9E
DV9N
DV9S
DV10B
DV10C
DV10E
DV10N
DV10S
Sample

37.

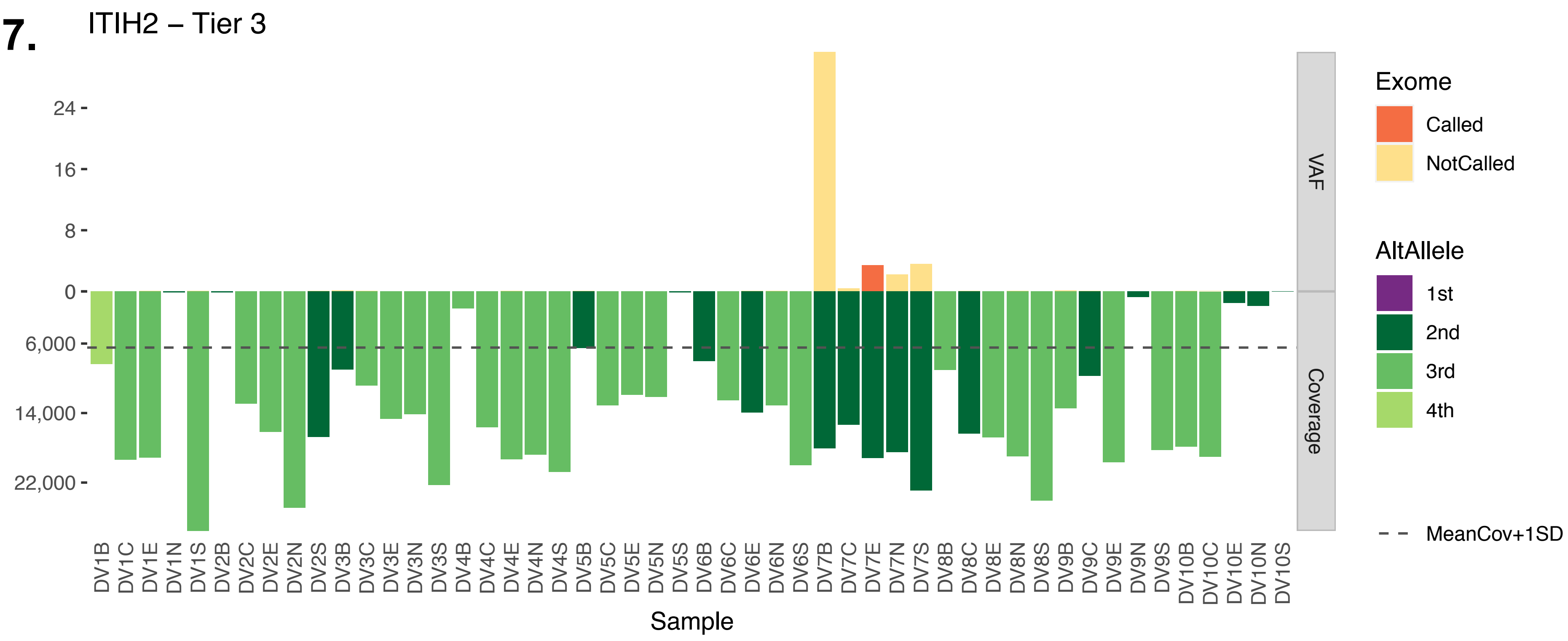

ITIH2 – Tier 3
24
16
8
0
6,000
14,000
22,000
VAF
Coverage
DV1B
DV1C
DV1E
DV1N
DV1S
DV2B
DV2C
DV2E
DV2N
DV2S
DV3B
DV3C
DV3E
DV3N
DV3S
DV4B
DV4C
DV4E
DV4N
DV4S
DV5B
DV5C
DV5E
DV5N
DV5S
DV6B
DV6C
DV6E
DV6N
DV6S
DV7B
DV7C
DV7E
DV7N
DV7S
DV8B
DV8C
DV8E
DV8N
DV8S
DV9B
DV9C
DV9E
DV9N
DV9S
DV10B
DV10C
DV10E
DV10N
DV10S
Sample
Exome
Called
NotCalled
AltAllele
1st
2nd
3rd
4th
MeanCov+1SD

38.

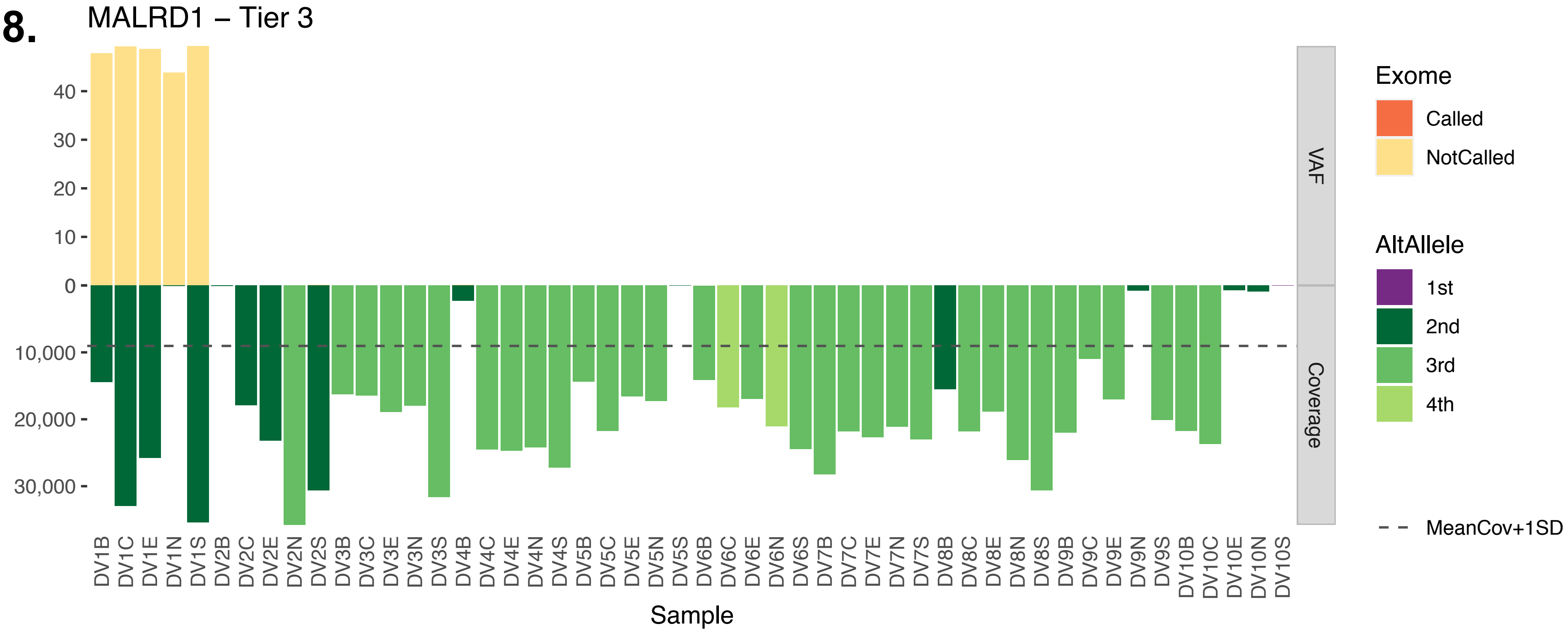

MALRD1 – Tier 3
40
30
20
10
0
10,000
20,000
30,000
VAF
Coverage
Exome
Called
NotCalled
AltAllele
1st
2nd
3rd
4th
MeanCov+1SD
DV1B DV1C DV1E DV1N DV1S DV2B DV2C DV2E DV2N DV2S DV3B DV3C DV3E DV3N DV3S DV4B DV4C DV4E DV4N DV4S DV5B DV5C DV5E DV5N DV5S DV6B DV6C DV6E DV6N DV6S DV7B DV7C DV7E DV7N DV7S DV8B DV8C DV8E DV8N DV8S DV9B DV9C DV9E DV9N DV9S DV10B DV10C DV10E DV10N DV10S
Sample

39.

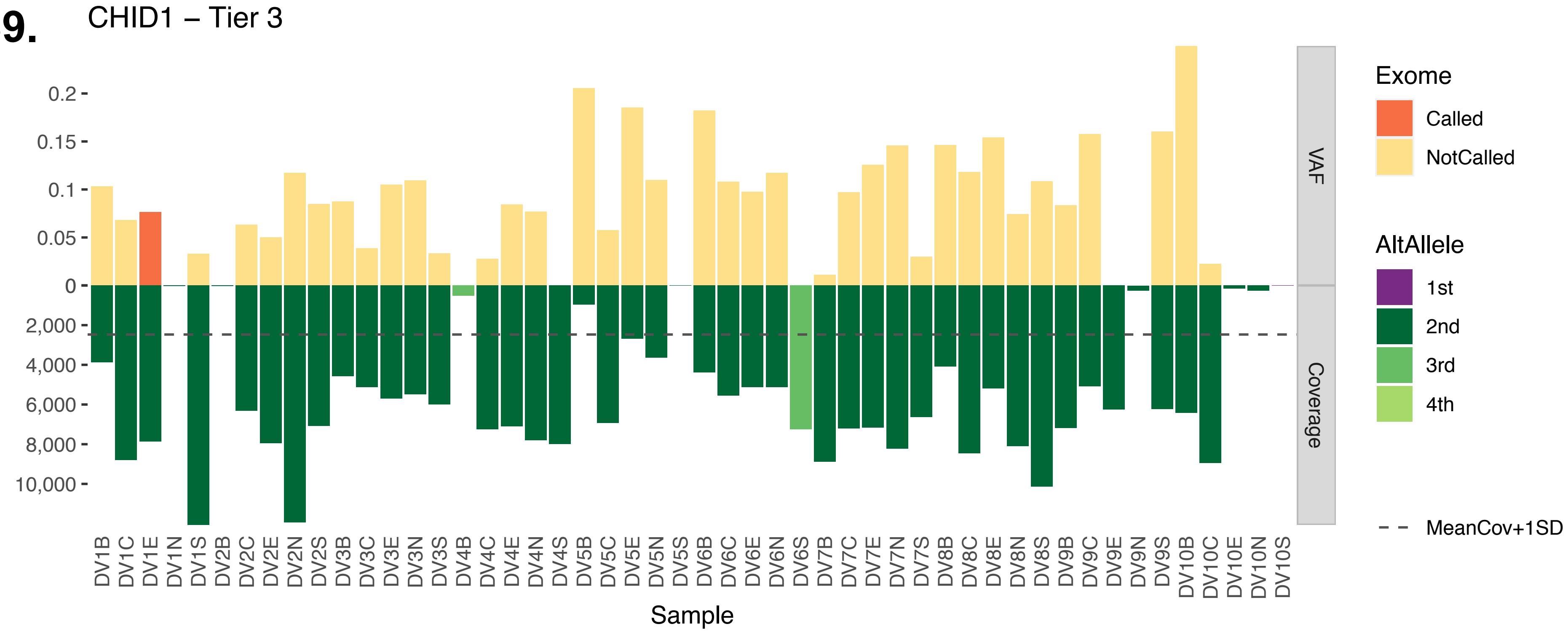
CHID1 – Tier 3
0.2
0.15
0.1
0.05
0
2,000
4,000
6,000
8,000
10,000
VAF
Coverage
Exome
Called
NotCalled
AltAllele
1st
2nd
3rd
4th
MeanCov+1SD
DV1B
DV1C
DV1E
DV1N
DV1S
DV2B
DV2C
DV2E
DV2N
DV2S
DV3B
DV3C
DV3E
DV3N
DV3S
DV4B
DV4C
DV4E
DV4N
DV4S
DV5B
DV5C
DV5E
DV5N
DV5S
DV6B
DV6C
DV6E
DV6N
DV6S
DV7B
DV7C
DV7E
DV7N
DV7S
DV8B
DV8C
DV8E
DV8N
DV8S
DV9B
DV9C
DV9E
DV9N
DV9S
DV10B
DV10C
DV10E
DV10N
DV10S
Sample

40.

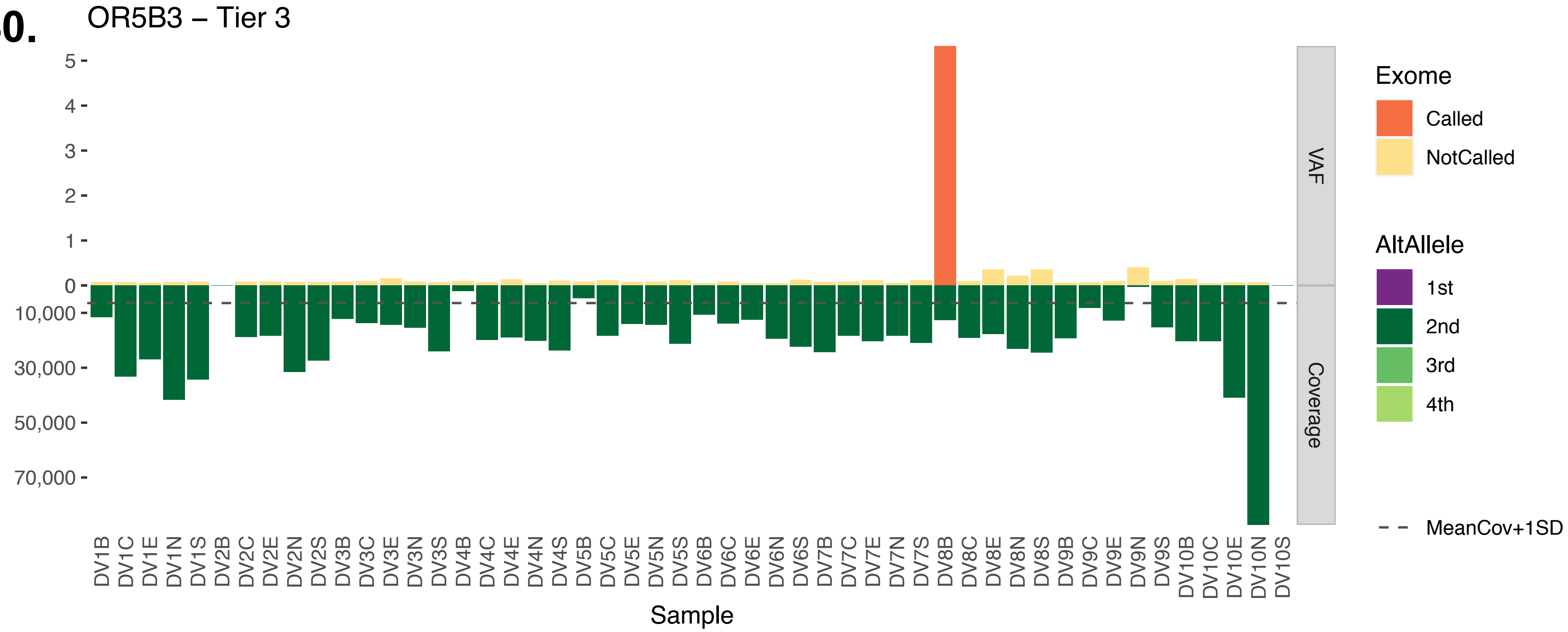
OR5B3 – Tier 3
5
4
3
2
1
0
10,000
30,000
50,000
70,000
VAF
Coverage
Exome
Called
NotCalled
AltAllele
1st
2nd
3rd
4th
MeanCov+1SD
DV1B DV1C DV1E DV1N DV1S DV2B DV2C DV2E DV2N DV2S DV3B DV3C DV3E DV3N DV3S DV4B DV4C DV4E DV4N DV4S DV5B DV5C DV5E DV5N DV5S DV6B DV6C DV6E DV6N DV6S DV7B DV7C DV7E DV7N DV7S DV8B DV8C DV8E DV8N DV8S DV9B DV9C DV9E DV9N DV9S DV10B DV10C DV10E DV10N DV10S
Sample

41.

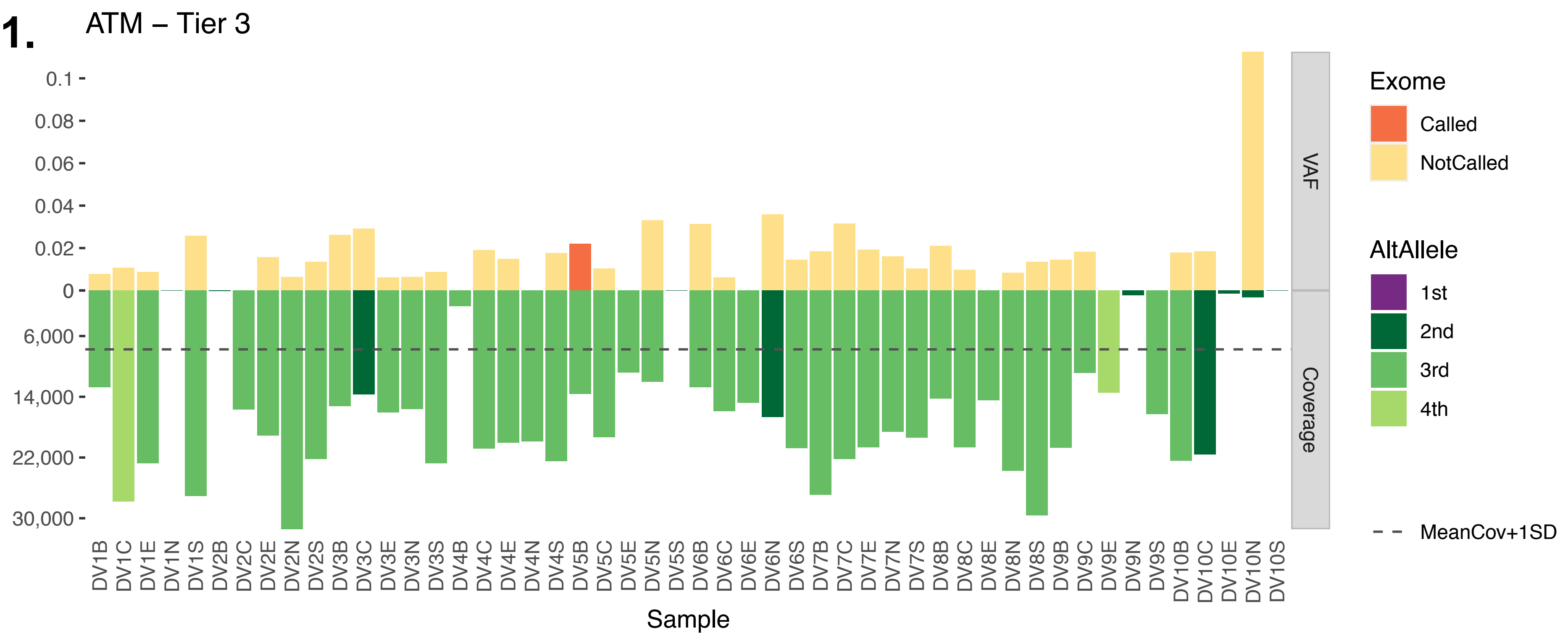
ATM – Tier 3
0.1
0.08
0.06
0.04
0.02
0
6,000
14,000
22,000
30,000
VAF
Coverage
DV1B
DV1C
DV1E
DV1N
DV1S
DV2B
DV2C
DV2E
DV2N
DV2S
DV3B
DV3C
DV3E
DV3N
DV3S
DV4B
DV4C
DV4E
DV4N
DV4S
DV5B
DV5C
DV5E
DV5N
DV5S
DV6B
DV6C
DV6E
DV6N
DV6S
DV7B
DV7C
DV7E
DV7N
DV7S
DV8B
DV8C
DV8E
DV8N
DV8S
DV9B
DV9C
DV9E
DV9N
DV9S
DV10B
DV10C
DV10E
DV10N
DV10S
Sample
Exome
Called
NotCalled
AltAllele
1st
2nd
3rd
4th
MeanCov+1SD

42.

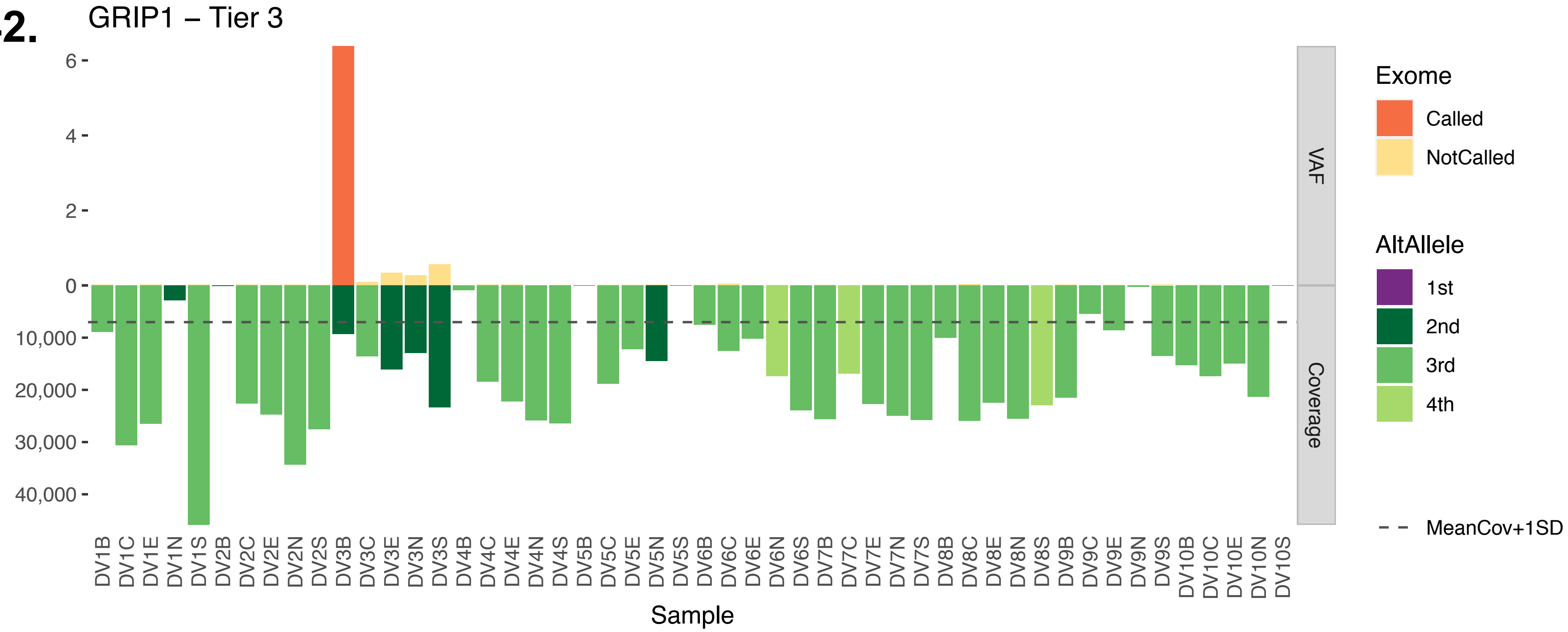

GRIP1 – Tier 3
6
4
2
0
10,000
20,000
30,000
40,000
VAF
Coverage
Exome
Called
NotCalled
AltAllele
1st
2nd
3rd
4th
MeanCov+1SD
DV1B DV1C DV1E DV1N DV1S DV2B DV2C DV2E DV2N DV2S DV3B DV3C DV3E DV3N DV3S DV4B DV4C DV4E DV4N DV4S DV5B DV5C DV5E DV5N DV5S DV6B DV6C DV6E DV6N DV6S DV7B DV7C DV7E DV7N DV7S DV8B DV8C DV8E DV8N DV8S DV9B DV9C DV9E DV9N DV9S DV10B DV10C DV10E DV10N DV10S
Sample

43.

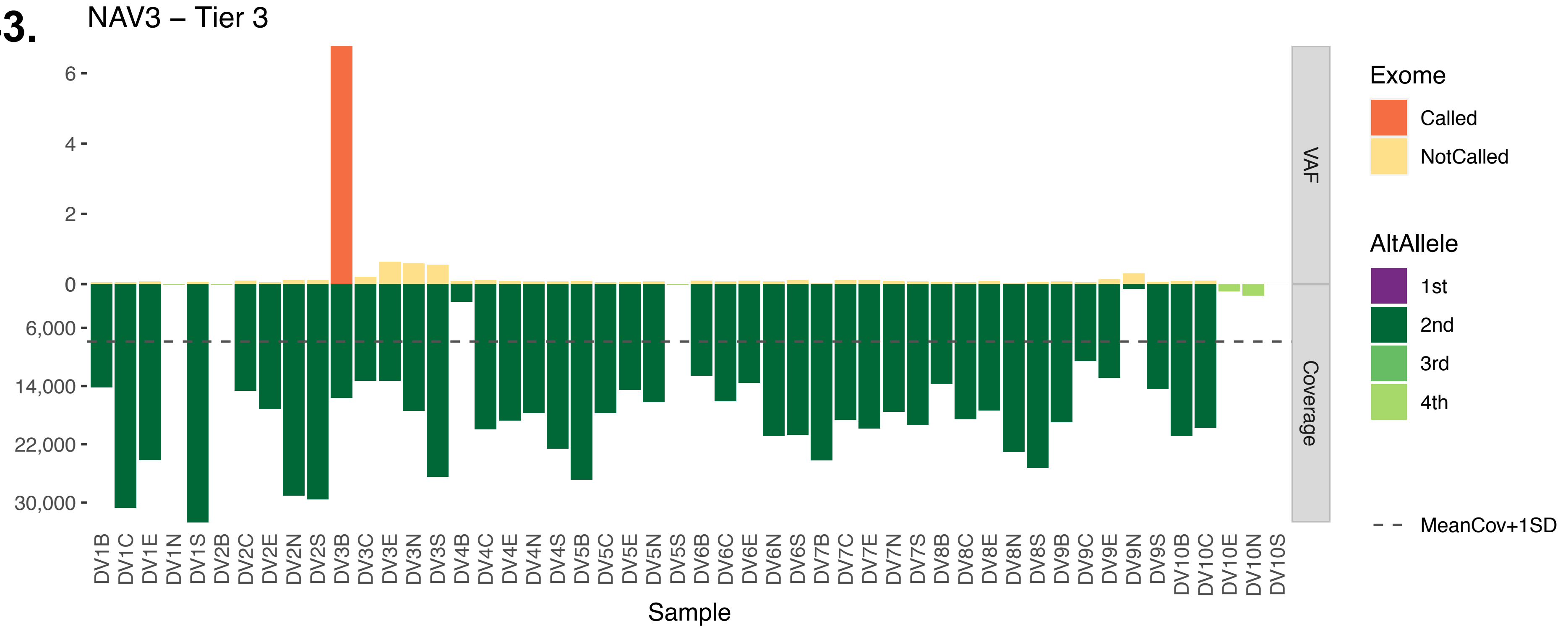
NAV3 – Tier 3
6
4
2
0
6,000
14,000
22,000
30,000
VAF
Coverage
Exome
Called
NotCalled
AltAllele
1st
2nd
3rd
4th
MeanCov+1SD
DV1B
DV1C
DV1E
DV1N
DV1S
DV2B
DV2C
DV2E
DV2N
DV2S
DV3B
DV3C
DV3E
DV3N
DV3S
DV4B
DV4C
DV4E
DV4N
DV4S
DV5B
DV5C
DV5E
DV5N
DV5S
DV6B
DV6C
DV6E
DV6N
DV6S
DV7B
DV7C
DV7E
DV7N
DV7S
DV8B
DV8C
DV8E
DV8N
DV8S
DV9B
DV9C
DV9E
DV9N
DV9S
DV10B
DV10C
DV10E
DV10N
DV10S
Sample

44.

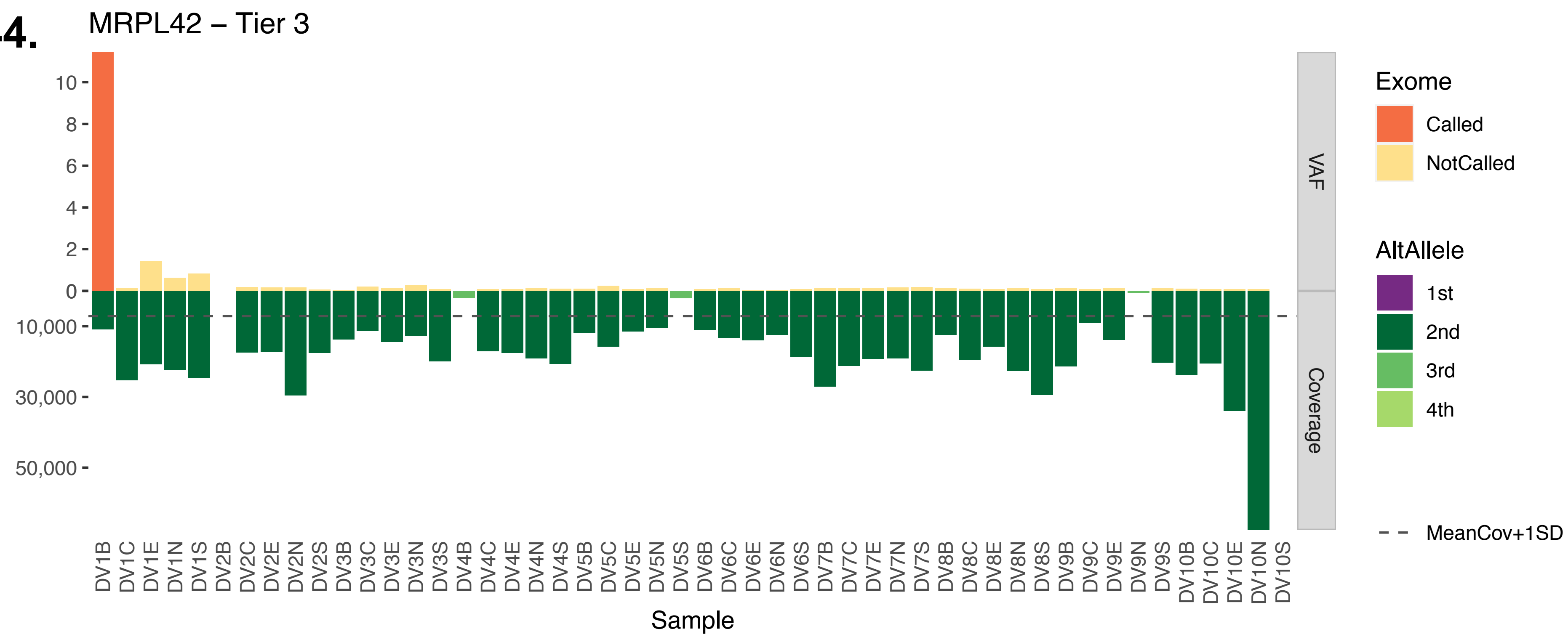
MRPL42 – Tier 3
10
8
6
4
2
0
10,000
30,000
50,000
VAF
Coverage
Exome
Called
NotCalled
AltAllele
1st
2nd
3rd
4th
MeanCov+1SD
DV1B DV1C DV1E DV1N DV1S DV2B DV2C DV2E DV2N DV2S DV3B DV3C DV3E DV3N DV3S DV4B DV4C DV4E DV4N DV4S DV5B DV5C DV5E DV5N DV5S DV6B DV6C DV6E DV6N DV6S DV7B DV7C DV7E DV7N DV7S DV8B DV8C DV8E DV8N DV8S DV9B DV9C DV9E DV9N DV9S DV10B DV10C DV10E DV10N DV10S
Sample

45.

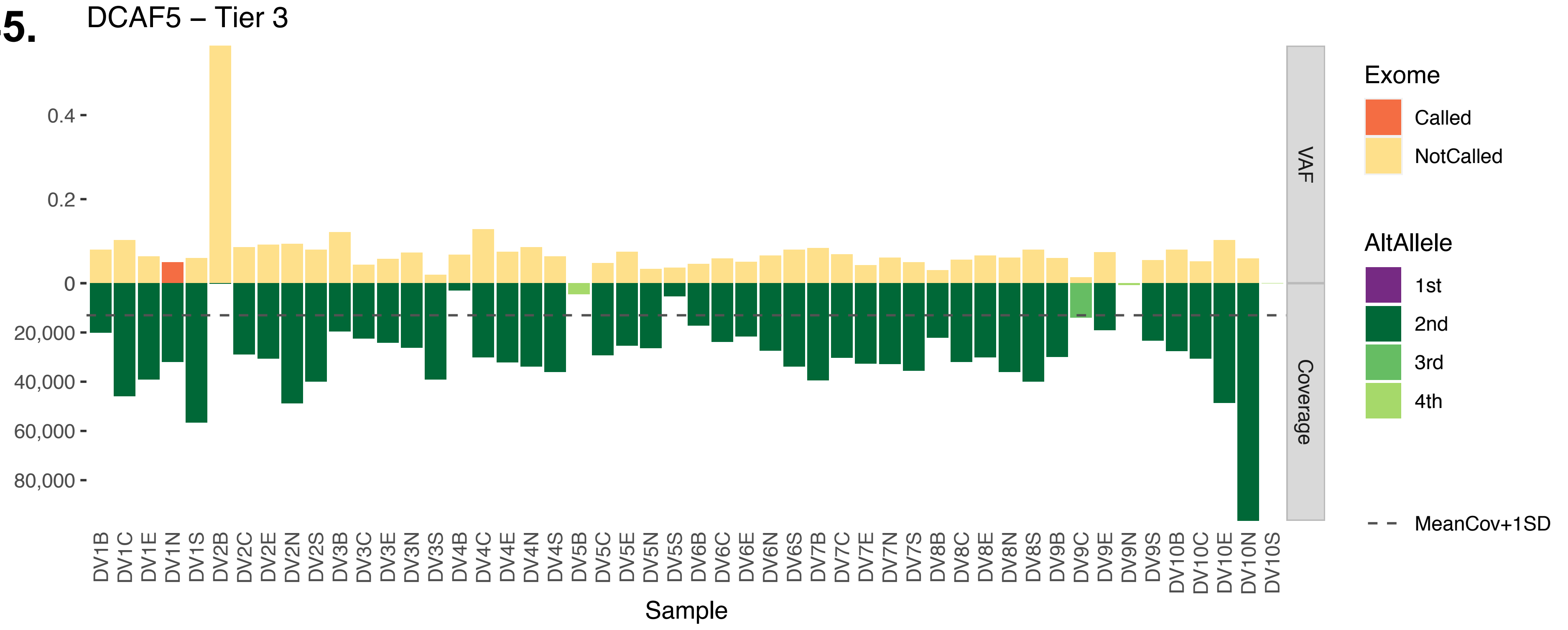

DCAF5 – Tier 3
0.4
0.2
0
20,000
40,000
60,000
80,000
VAF
Coverage
Exome
Called
NotCalled
AltAllele
1st
2nd
3rd
4th
MeanCov+1SD
DV1B
DV1C
DV1E
DV1N
DV1S
DV2B
DV2C
DV2E
DV2N
DV2S
DV3B
DV3C
DV3E
DV3N
DV3S
DV4B
DV4C
DV4E
DV4N
DV4S
DV5B
DV5C
DV5E
DV5N
DV5S
DV6B
DV6C
DV6E
DV6N
DV6S
DV7B
DV7C
DV7E
DV7N
DV7S
DV8B
DV8C
DV8E
DV8N
DV8S
DV9B
DV9C
DV9E
DV9N
DV9S
DV10B
DV10C
DV10E
DV10N
DV10S
Sample

46.

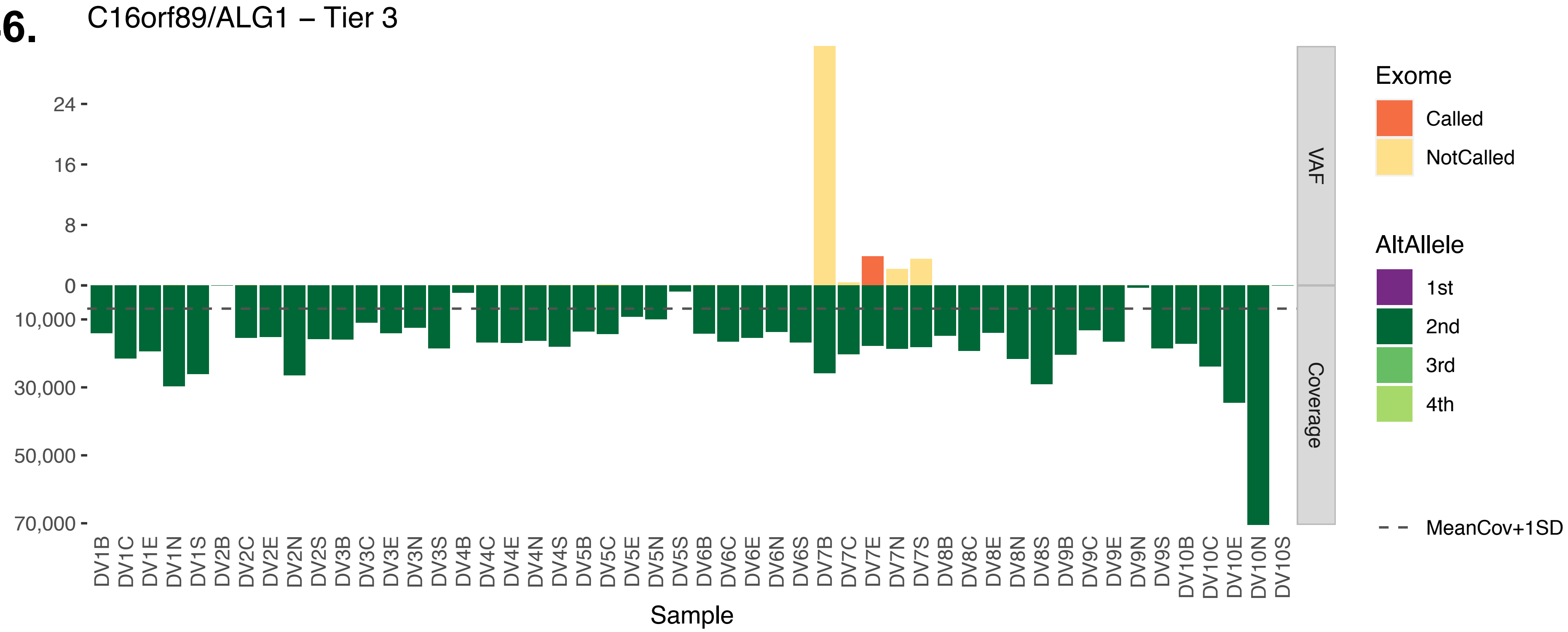

C16orf89/ALG1 – Tier 3
24
16
8
0
10,000
30,000
50,000
70,000
VAF
Coverage
DV1B
DV1C
DV1E
DV1N
DV1S
DV2B
DV2C
DV2E
DV2N
DV2S
DV3B
DV3C
DV3E
DV3N
DV3S
DV4B
DV4C
DV4E
DV4N
DV4S
DV5B
DV5C
DV5E
DV5N
DV5S
DV6B
DV6C
DV6E
DV6N
DV6S
DV7B
DV7C
DV7E
DV7N
DV7S
DV8B
DV8C
DV8E
DV8N
DV8S
DV9B
DV9C
DV9E
DV9N
DV9S
DV10B
DV10C
DV10E
DV10N
DV10S
Sample
Exome
Called
NotCalled
AltAllele
1st
2nd
3rd
4th
MeanCov+1SD

47.

NEURL4,GPS2 – Tier 3

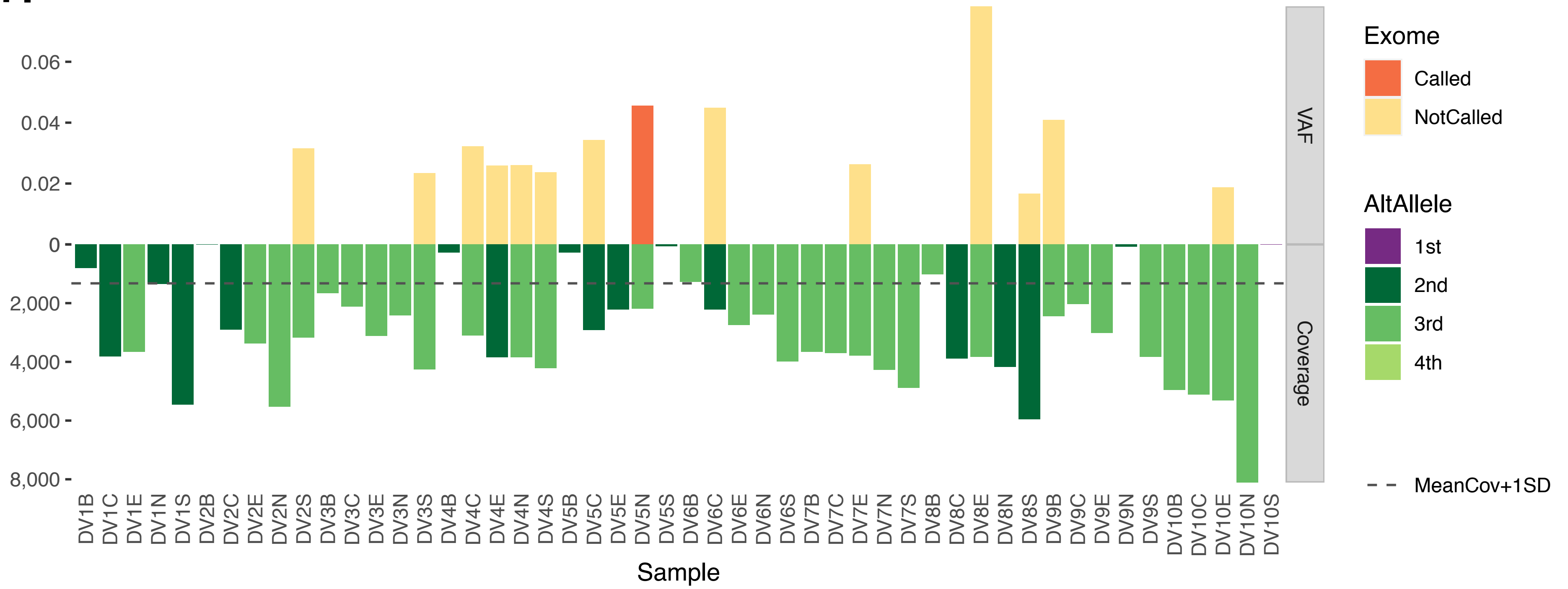

48.

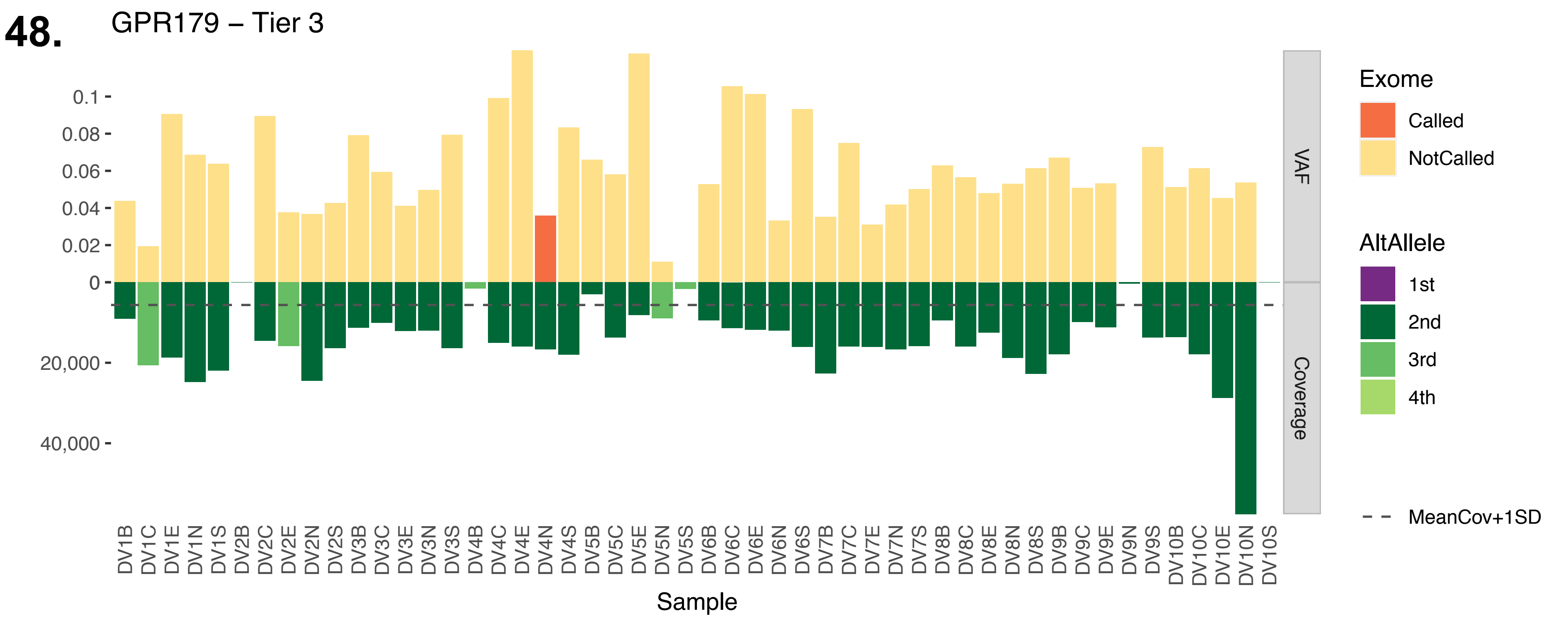
GPR179 – Tier 3
0.1
0.08
0.06
0.04
0.02
0
20,000
40,000
VAF
Coverage
DV1B
DV1C
DV1E
DV1N
DV1S
DV2B
DV2C
DV2E
DV2N
DV2S
DV3B
DV3C
DV3E
DV3N
DV3S
DV4B
DV4C
DV4E
DV4N
DV4S
DV5B
DV5C
DV5E
DV5N
DV5S
DV6B
DV6C
DV6E
DV6N
DV6S
DV7B
DV7C
DV7E
DV7N
DV7S
DV8B
DV8C
DV8E
DV8N
DV8S
DV9B
DV9C
DV9E
DV9N
DV9S
DV10B
DV10C
DV10E
DV10N
DV10S
Sample
Exome
Called
NotCalled
AltAllele
1st
2nd
3rd
4th
MeanCov+1SD

49.

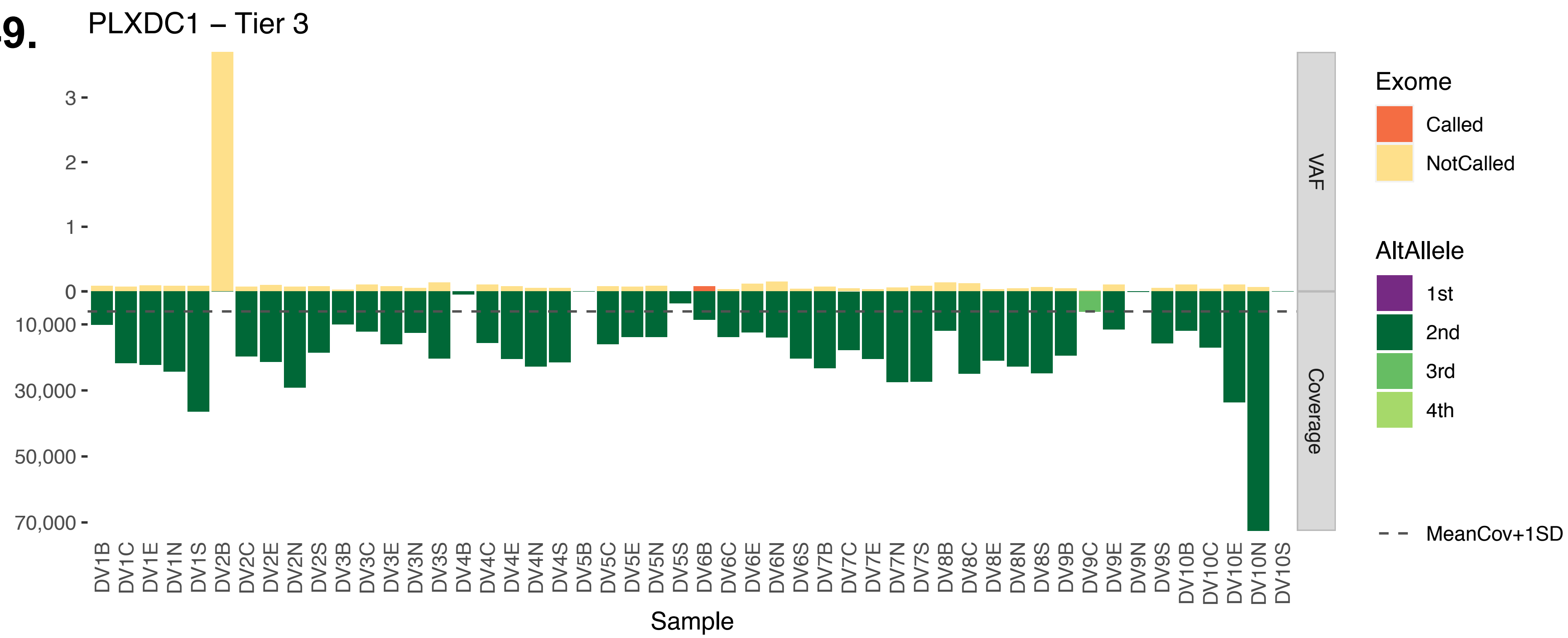
PLXDC1 – Tier 3
3
2
1
0
10,000
30,000
50,000
70,000
VAF
Coverage
Sample
DV1B
DV1C
DV1E
DV1N
DV1S
DV2B
DV2C
DV2E
DV2N
DV2S
DV3B
DV3C
DV3E
DV3N
DV3S
DV4B
DV4C
DV4E
DV4N
DV4S
DV5B
DV5C
DV5E
DV5N
DV5S
DV6B
DV6C
DV6E
DV6N
DV6S
DV7B
DV7C
DV7E
DV7N
DV7S
DV8B
DV8C
DV8E
DV8N
DV8S
DV9B
DV9C
DV9E
DV9N
DV9S
DV10B
DV10C
DV10E
DV10N
DV10S
Exome
Called
NotCalled
AltAllele
1st
2nd
3rd
4th
MeanCov+1SD

50.

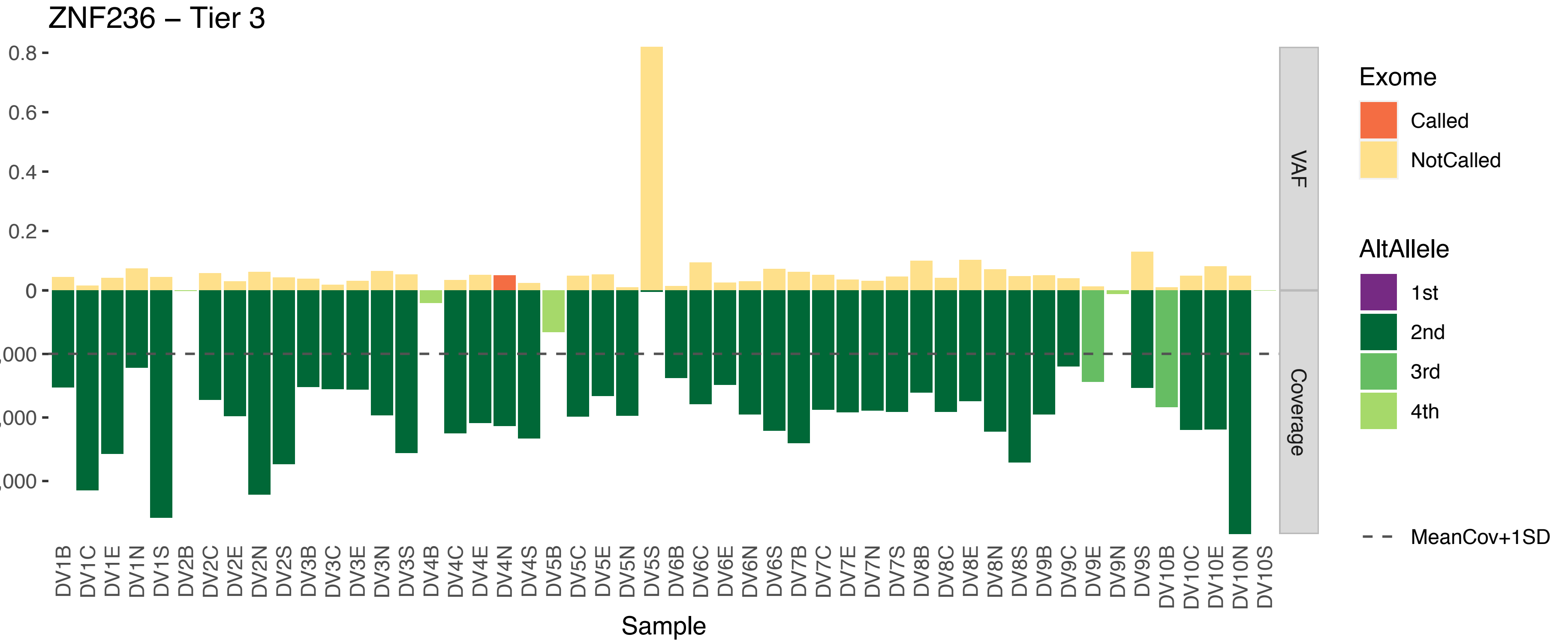
ZNF236 – Tier 3
0.8
0.6
0.4
0.2
0
10,000
20,000
30,000
VAF
Coverage
DV1B
DV1C
DV1E
DV1N
DV1S
DV2B
DV2C
DV2E
DV2N
DV2S
DV3B
DV3C
DV3E
DV3N
DV3S
DV4B
DV4C
DV4E
DV4N
DV4S
DV5B
DV5C
DV5E
DV5N
DV5S
DV6B
DV6C
DV6E
DV6N
DV6S
DV7B
DV7C
DV7E
DV7N
DV7S
DV8B
DV8C
DV8E
DV8N
DV8S
DV9B
DV9C
DV9E
DV9N
DV9S
DV10B
DV10C
DV10E
DV10N
DV10S
Sample
Exome
Called
NotCalled
AltAllele
1st
2nd
3rd
4th
MeanCov+1SD

51.

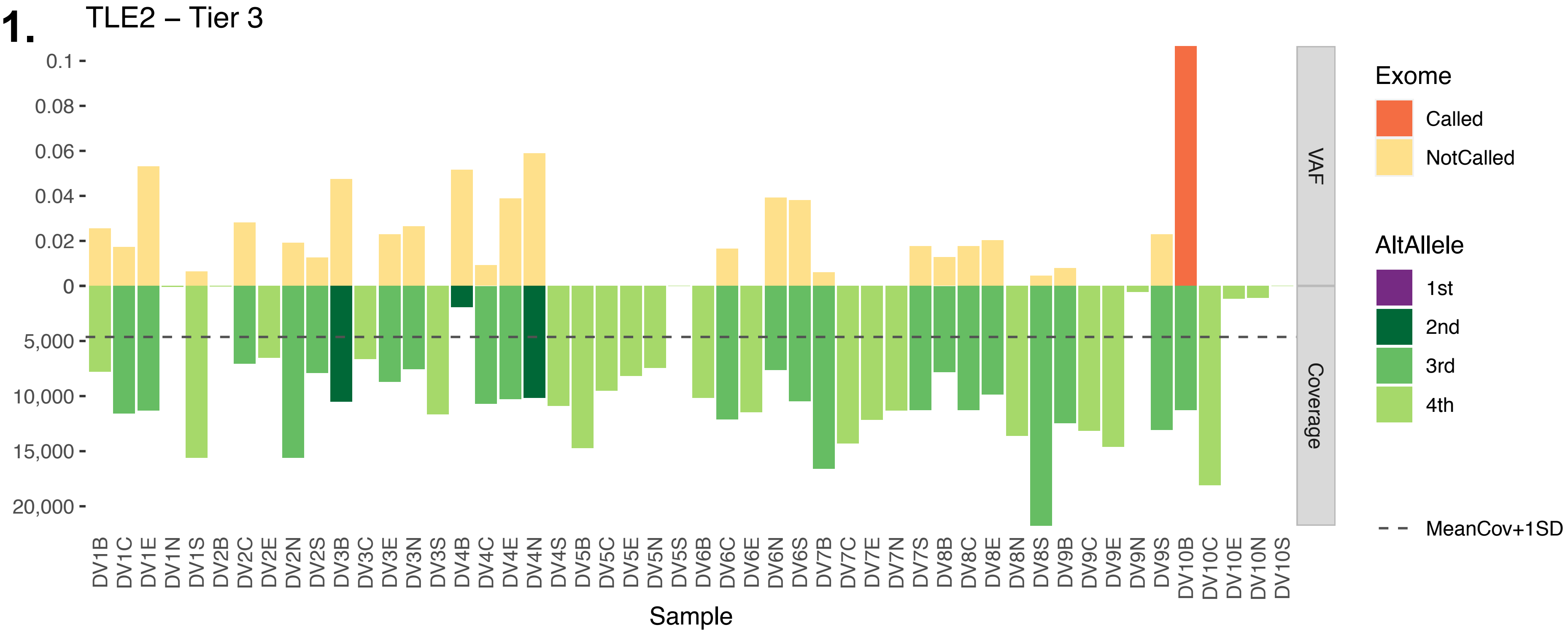
TLE2 – Tier 3
0.1
0.08
0.06
0.04
0.02
0
5,000
10,000
15,000
20,000
VAF
Coverage
DV1B
DV1C
DV1E
DV1N
DV1S
DV2B
DV2C
DV2E
DV2N
DV2S
DV3B
DV3C
DV3E
DV3N
DV3S
DV4B
DV4C
DV4E
DV4N
DV4S
DV5B
DV5C
DV5E
DV5N
DV5S
DV6B
DV6C
DV6E
DV6N
DV6S
DV7B
DV7C
DV7E
DV7N
DV7S
DV8B
DV8C
DV8E
DV8N
DV8S
DV9B
DV9C
DV9E
DV9N
DV9S
DV10B
DV10C
DV10E
DV10N
DV10S
Sample
Exome
Called
NotCalled
AltAllele
1st
2nd
3rd
4th
MeanCov+1SD

52.

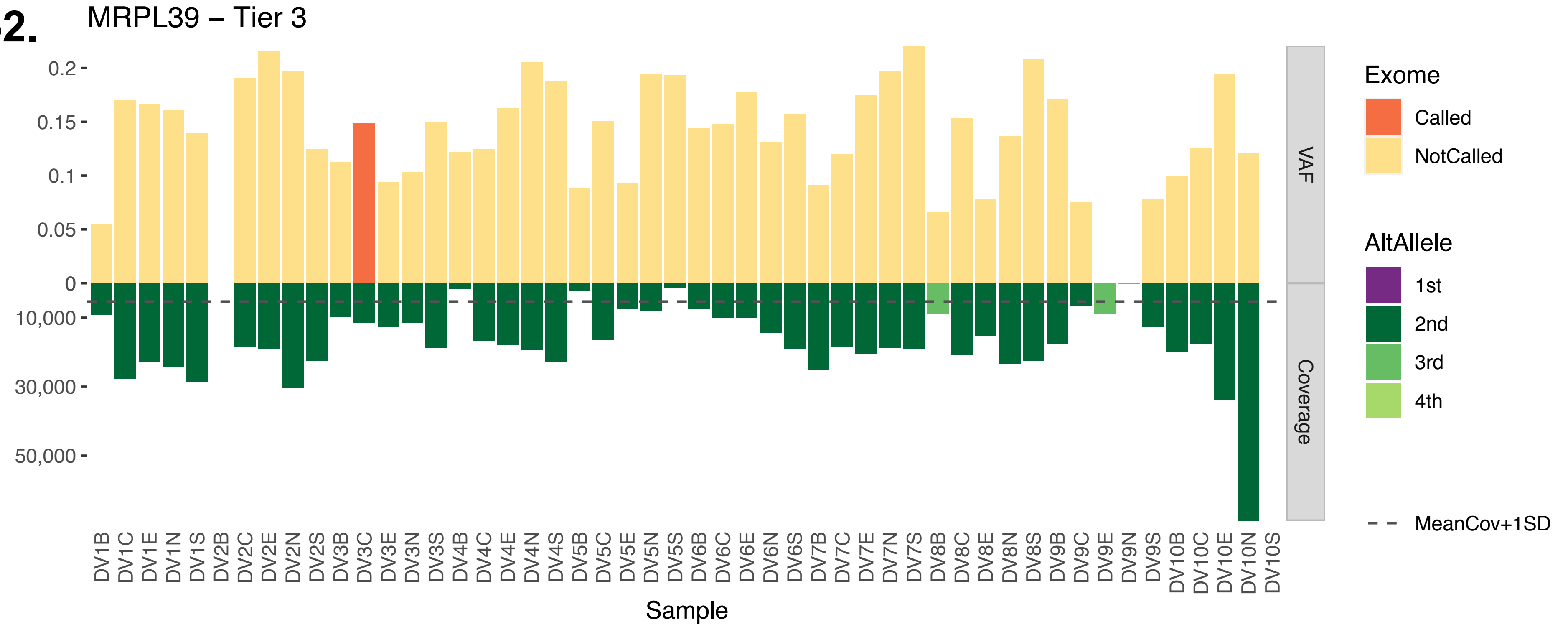

MRPL39 – Tier 3
0.2
0.15
0.1
0.05
0
10,000
30,000
50,000
VAF
Coverage
Exome
Called
NotCalled
AltAllele
1st
2nd
3rd
4th
MeanCov+1SD
DV1B DV1C DV1E DV1N DV1S DV2B DV2C DV2E DV2N DV2S DV3B DV3C DV3E DV3N DV3S DV4B DV4C DV4E DV4N DV4S DV5B DV5C DV5E DV5N DV5S DV6B DV6C DV6E DV6N DV6S DV7B DV7C DV7E DV7N DV7S DV8B DV8C DV8E DV8N DV8S DV9B DV9C DV9E DV9N DV9S DV10B DV10C DV10E DV10N DV10S
Sample

53.

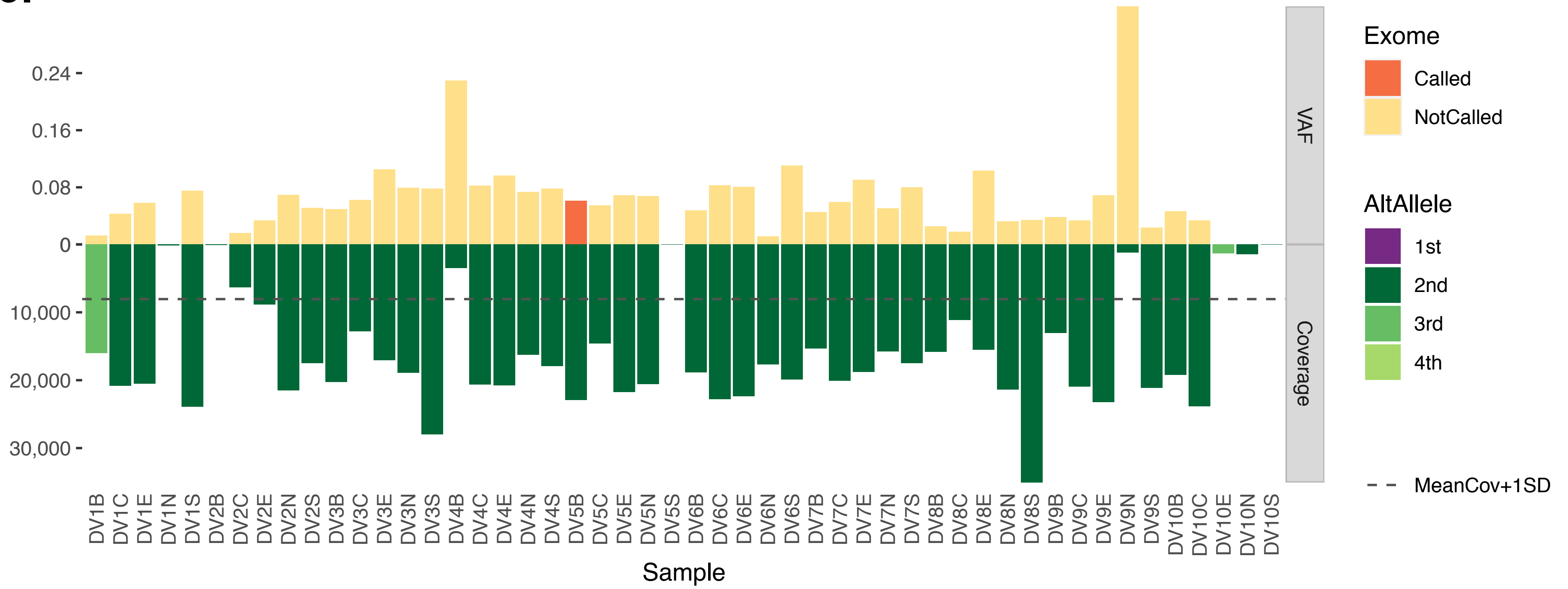

GUCD1,SNRPD3 – Tier 3
0.24
0.16
0.08
0
10,000
20,000
30,000
VAF
Coverage
Exome
Called
NotCalled
AltAllele
1st
2nd
3rd
4th
MeanCov+1SD
DV1B DV1C DV1E DV1N DV1S DV2B DV2C DV2E DV2N DV2S DV3B DV3C DV3E DV3N DV3S DV4B DV4C DV4E DV4N DV4S DV5B DV5C DV5E DV5N DV5S DV6B DV6C DV6E DV6N DV6S DV7B DV7C DV7E DV7N DV7S DV8B DV8C DV8E DV8N DV8S DV9B DV9C DV9E DV9N DV9S DV10B DV10C DV10E DV10N DV10S
Sample

54.

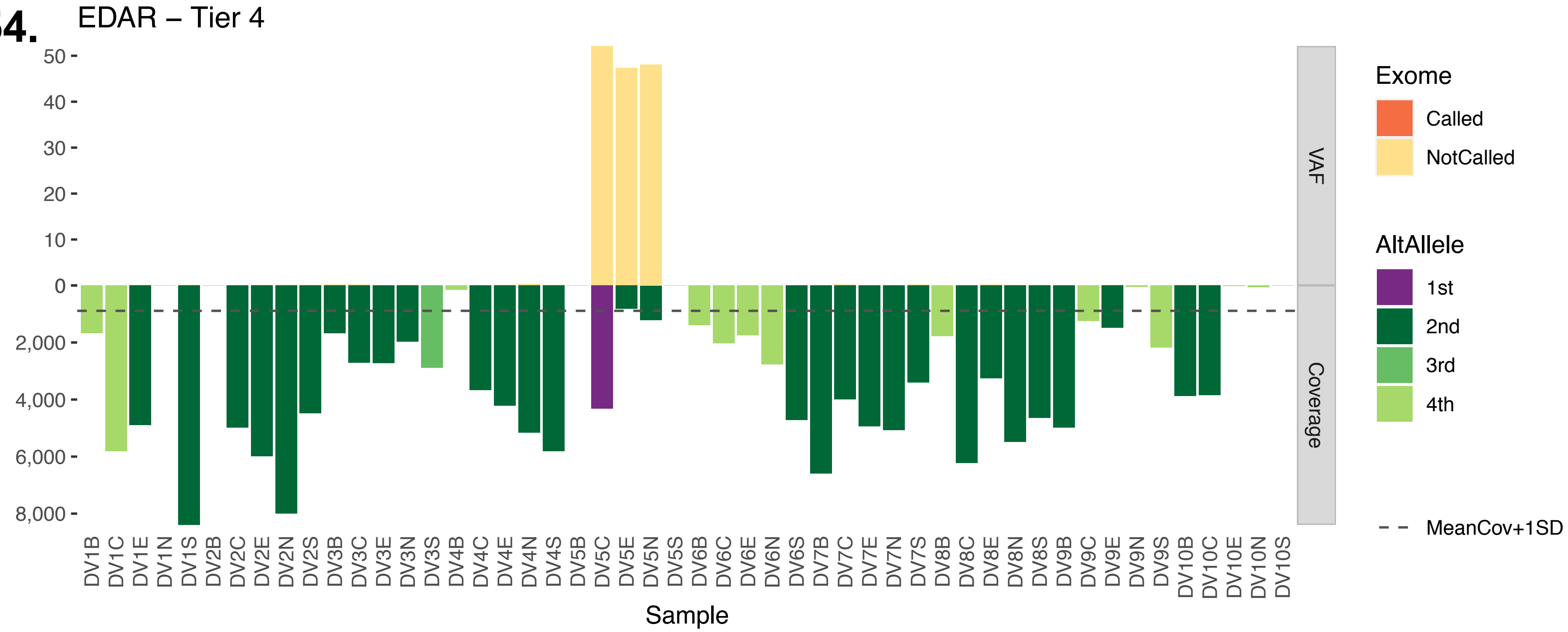
EDAR – Tier 4
50
40
30
20
10
0
2,000
4,000
6,000
8,000
VAF
Coverage
Exome
Called
NotCalled
AltAllele
1st
2nd
3rd
4th
MeanCov+1SD
DV1B DV1C DV1E DV1N DV1S DV2B DV2C DV2E DV2N DV2S DV3B DV3C DV3E DV3N DV3S DV4B DV4C DV4E DV4N DV4S DV5B DV5C DV5E DV5N DV5S DV6B DV6C DV6E DV6N DV6S DV7B DV7C DV7E DV7N DV7S DV8B DV8C DV8E DV8N DV8S DV9B DV9C DV9E DV9N DV9S DV10B DV10C DV10E DV10N DV10S
Sample

55.

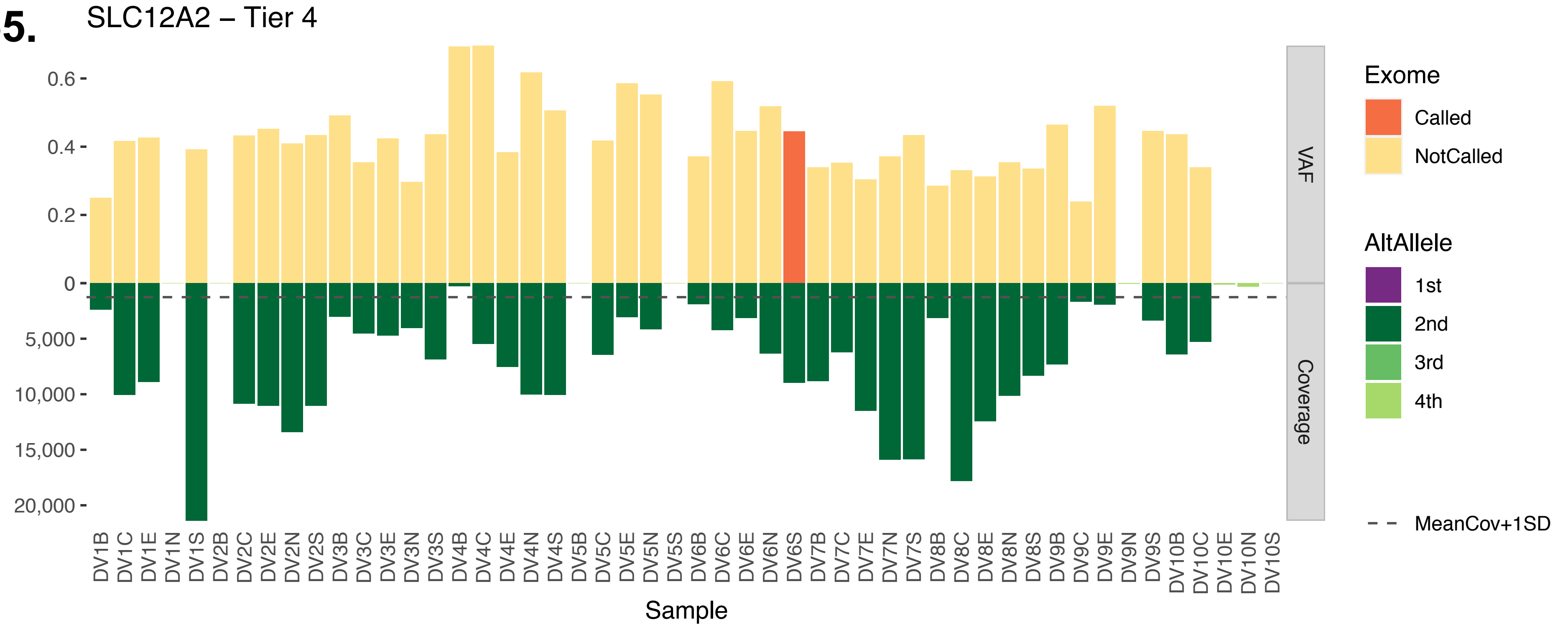

SLC12A2 – Tier 4
0.6
0.4
0.2
0
5,000
10,000
15,000
20,000
VAF
Coverage
DV1B DV1C DV1E DV1N DV1S DV2B DV2C DV2E DV2N DV2S DV3B DV3C DV3E DV3N DV3S DV4B DV4C DV4E DV4N DV4S DV5B DV5C DV5E DV5N DV5S DV6B DV6C DV6E DV6N DV6S DV7B DV7C DV7E DV7N DV7S DV8B DV8C DV8E DV8N DV8S DV9B DV9C DV9E DV9N DV9S DV10B DV10C DV10E DV10N DV10S
Sample
Exome
Called
NotCalled
AltAllele
1st
2nd
3rd
4th
MeanCov+1SD

56.

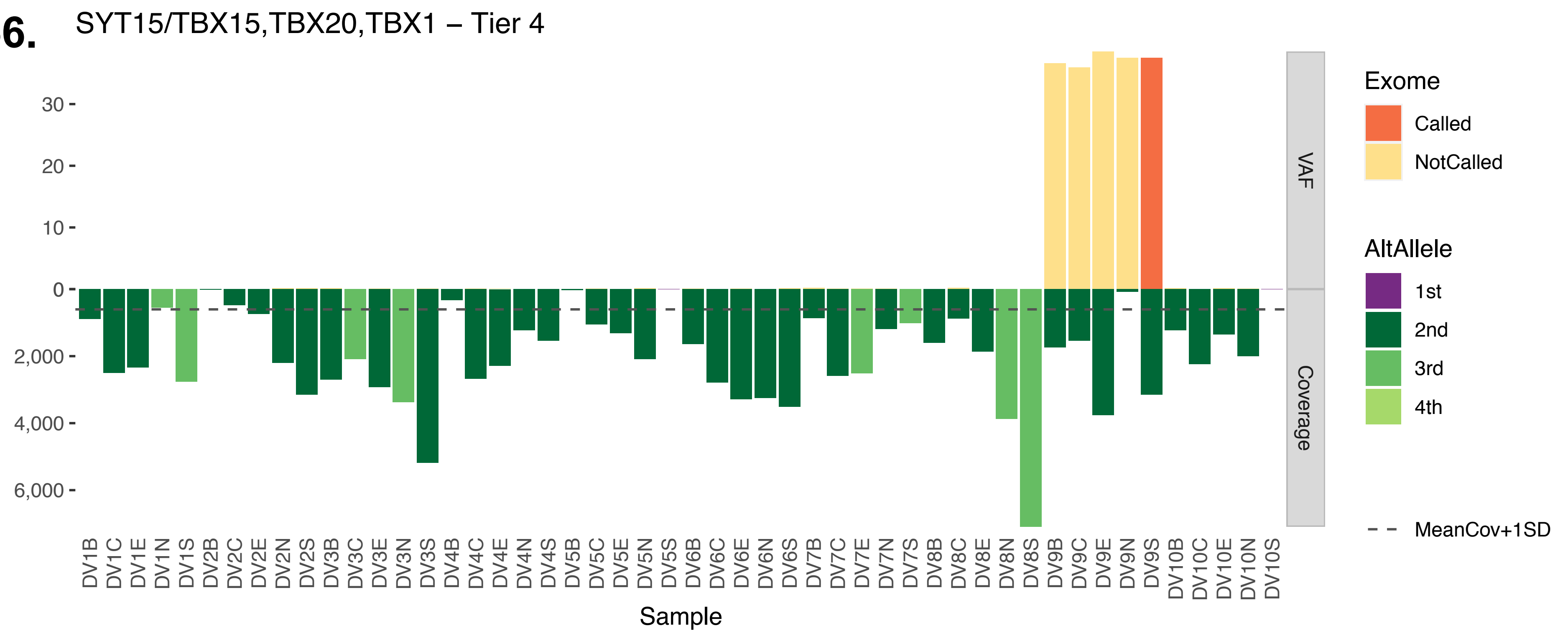
SYT15/TBX15,TBX20,TBX1 – Tier 4
30
20
10
0
2,000
4,000
6,000
VAF
Coverage
Exome
Called
NotCalled
AltAllele
1st
2nd
3rd
4th
MeanCov+1SD
DV1B DV1C DV1E DV1N DV1S DV2B DV2C DV2E DV2N DV2S DV3B DV3C DV3E DV3N DV3S DV4B DV4C DV4E DV4N DV4S DV5B DV5C DV5E DV5N DV5S DV6B DV6C DV6E DV6N DV6S DV7B DV7C DV7E DV7N DV7S DV8B DV8C DV8E DV8N DV8S DV9B DV9C DV9E DV9N DV9S DV10B DV10C DV10E DV10N DV10S
Sample

57. TRPM5 – Tier 4

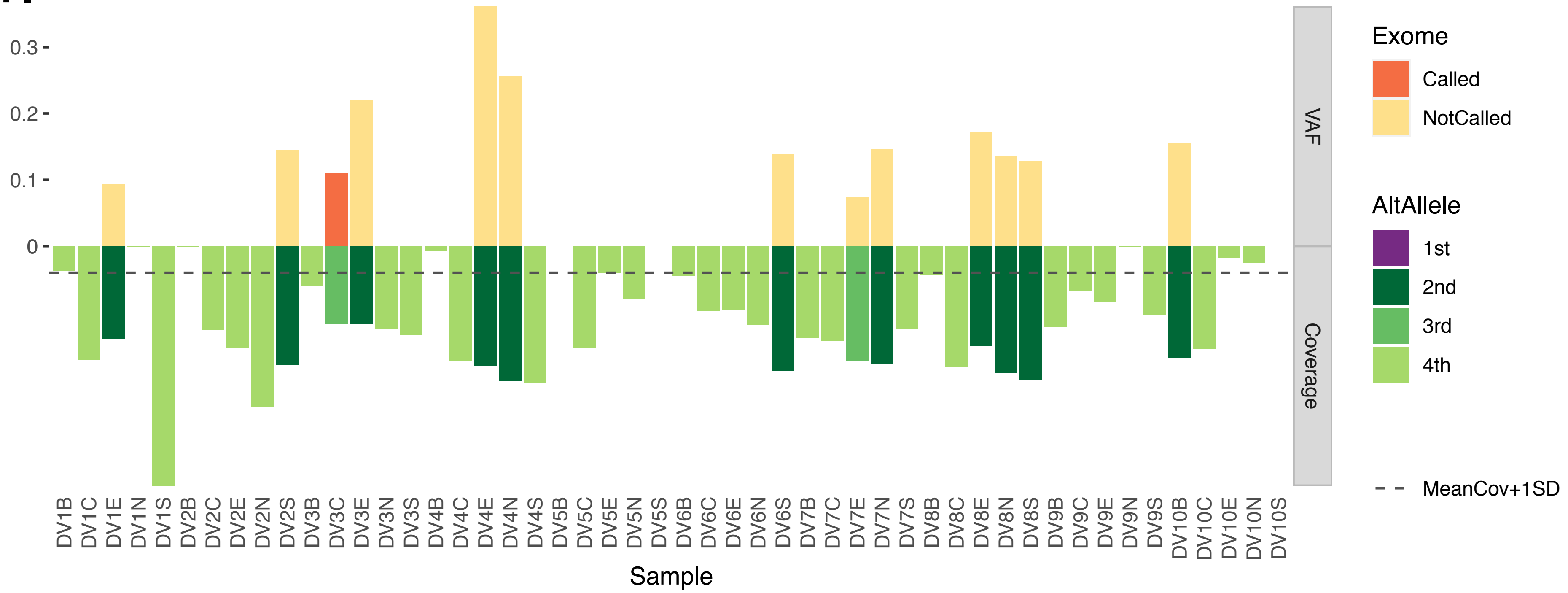

Supplement: Supplementary file 3 [file DataSheet1.PDF]
